# Expanded Access to Fluoroformamidines via a Modular Synthetic Pathway

**DOI:** 10.1021/acs.orglett.4c00131

**Published:** 2024-02-07

**Authors:** James A. Vogel, Kirya F. Miller, Eunjeong Shin, Jenna M. Krussman, Patrick R. Melvin

**Affiliations:** † Department of Chemistry, Bryn Mawr College, Bryn Mawr, Pennsylvania 19010, United States

## Abstract

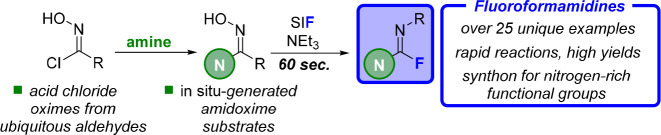

Fluoroformamidines are an underutilized and understudied functional group despite combining two of the most highly prized elements in drug design: nitrogen and fluorine. We report a practical and modular synthesis of fluoroformamidines via the rearrangement of *in situ*-generated amidoximes. High yields in just 60 s at room temperature highlight the efficiency of this protocol. Furthermore, fluoroformamidines proved to be useful intermediates in the synthesis of diverse ureas and carbamimidates.

Nitrogen-containing functional groups are widely recognized as significant contributors in the drug design realm.^1,2^ In fact, nearly half of the 40 most common functional groups found in bioactive molecules contain at least one nitrogen atom, with three of those functional groups (urea: 5.4%; guanidine: 2.6%; amidine: 1.5%) positioning multiple nitrogens in close proximity.^3^ Similarly, fluorine has recently grown in prominence due to an abundance of positive influences its incorporation can have on drug candidates.^4−7^ As of 2023, roughly 20% of pharmaceuticals and 50% of agrochemicals contain at least one fluorine atom, with these numbers continuing to increase each year.^8^ Despite the prevalence of both nitrogen and fluorine in small molecule drug candidates, these two highly utilized elements are rarely combined in the same functional group.

Fluoroformamidines (Figure 1A) represent one such moiety that brings together multiple nitrogens with fluorine. However, this functional group is critically understudied, with only a singular report focusing on fluoroformamidines in the last 30 years.^9^ In contrast, carbamoyl fluorides, the oxygen analogues of fluoroformamidines, have recently garnered far more attention. While carbamoyl fluorides were shown to be competent protease and esterase inhibitors in the 1960s,^10^ it was not until the past 5 years that their applications have been greatly expanded,^11−13^ with this renewed interest largely attributed to the development of improved carbamoyl fluoride syntheses.^14−16^ Fluoroformamidines present similar application opportunities with the added benefit of greater tunability via the imidoyl nitrogen substituent. Furthermore, this motif could serve as a synthetic precursor to various nitrogen-rich functional groups such as ureas and carbamimidates.

**Figure 1:**
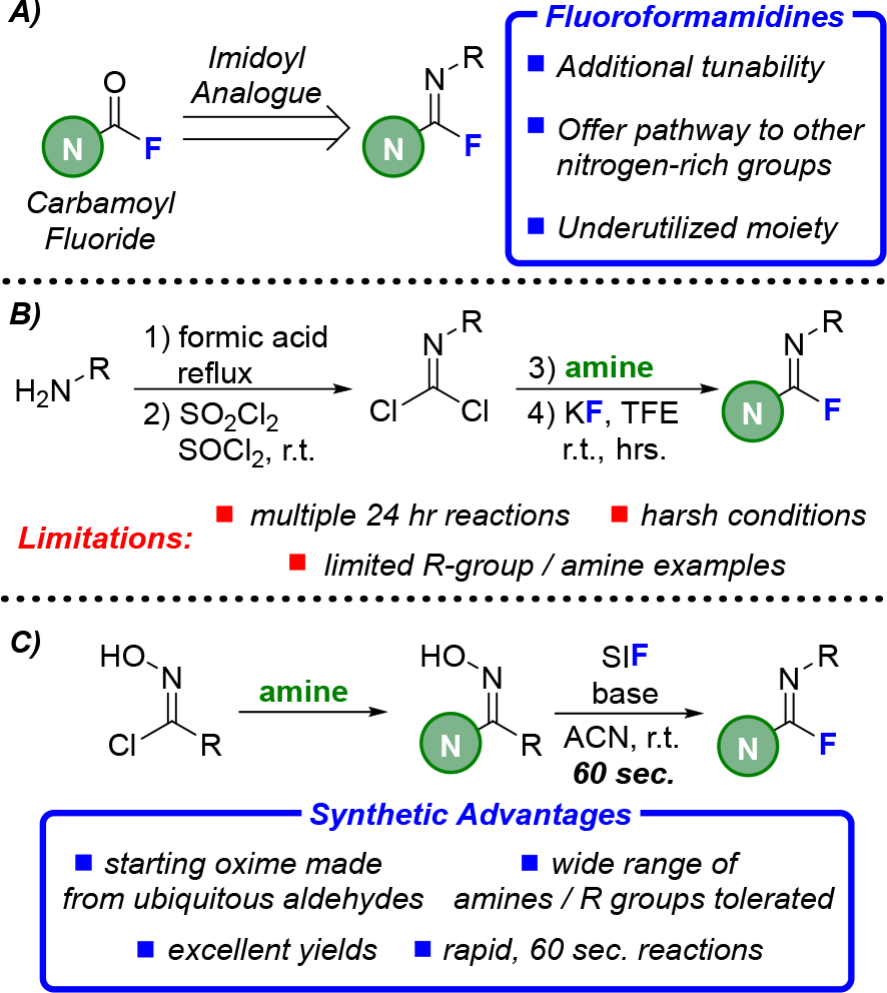
(A) Benefits of fluoroformamidines; (B) Previous synthesis of fluoroformamidines; (C) Proposed pathway and synthetic advantages.

The lack of attention given to fluoroformamidines is, in large part, due to a dearth of synthetic techniques that furnish this specific combination of nitrogen and fluorine. The only established procedure requires a tedious, multistep synthesis which utilizes harsh reagents and conditions throughout (Figure 1B).^9^ First, anilines are combined with formic acid at reflux temperatures to generate formanilides, which are then converted to carbonimidoyl dichlorides using sulfuryl chloride and thionyl chloride. Addition of an amine nucleophile produces the chloroformamidine; a halogen exchange furnishes the final fluoroformamidine product, but this requires several hours under anhydrous conditions.^9^ An alternative route would be the use of acid chloride oximes to generate amidoximes *in situ*, followed by a rearrangement facilitated by a sulfur(VI)–fluoride reagent (Figure 1C, see Supporting Information (SI) for further mechanism discussion). Notably, acid chloride oximes can be generated in a convenient and inexpensive two-step process from commercially available aldehydes, thereby offering a cheaper and more tunable synthesis.

Recently, our group demonstrated the propensity of sulfone iminium fluoride (SIF, see Scheme 1) reagents to carry out a similar transformation of ketoximes to imidoyl fluorides, requiring just 60 s at room temperature to reach quantitative yields.^17^ We envisioned that this same methodology could be transferred to *in situ*-generated amidoximes, forming the desired fluoroformamidine products quickly and efficiently (Figure 1C). In addition to isolating the novel fluoroformamidines, we endeavored to discern their stability and reactivity through the introduction of various oxygen nucleophiles, creating one-pot methodologies for the synthesis of urea and carbamimidate derivatives.

**Scheme 1:**
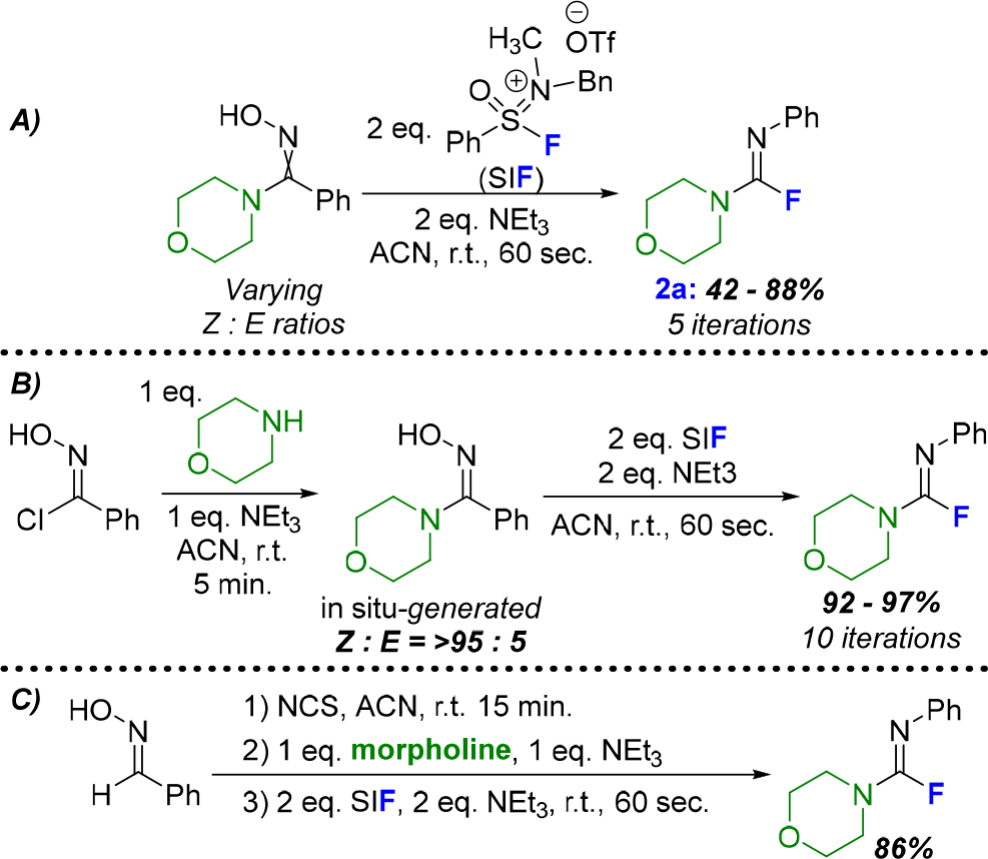
(A) Initial Success for the Rearrangement of Amidoxime to Fluoroformamidine, **2a**; (B) Revised Method Generating Amidoxime *in Situ* To Combat Reproducibility Issues; (C) One-Pot Synthesis of **2a** Starting from Aldoxime

To probe the potential of this transformation, our initial investigations made use of isolated 4-morpholinylphenylmethanone oxime as the model substrate under conditions that proved ideal for the previous ketoxime transformations: 2 equiv of triethylamine (NEt_3_) and SIF in acetonitrile at room temperature for 60 s.^17^ Gratifyingly, these conditions produced a 76% yield of the rearranged fluoroformamidine product (**2a**) by ^19^F NMR, demonstrating that the underlying principles of this synthetic pathway were sound. However, the yield of **2a** varied greatly between batches of isolated amidoxime substrate (42–88%, Scheme 1A), leading to investigations on reproducibility. Ultimately, these issues result from fluctuating ratios of amidoxime isomers; in the rearrangement mechanism, solely the Z stereoisomer is capable of participating; therefore, only those batches of amidoxime with a high Z:E ratio can produce significant quantities of fluoroformamidine. It was noted that freshly synthesized amidoxime typically led to higher yields of fluorinated product, which correlates with the observation that the Z stereoisomer slowly isomerizes to the inactive E version over time, with older batches (>1 week) producing significantly less **2a** by ^19^F NMR (see SI).

Seeking to combat these reproducibility issues, we looked to generate the amidoxime substrate *in situ* from the corresponding phenyl acid chloride oxime (**1a**), which demonstrates little to no isomerism issues. Combining **1a** with NEt_3_ begets an immediate transformation to the corresponding phenyl nitrile oxide, which rapidly forms the requisite *Z*-amidoxime upon addition of 1 equiv of morpholine. The retention of the Z configuration is attributed to a possible hydrogen bonding interaction between the hydroxyl group and the incoming nitrogen. Subsequent introduction of SIF forms **2a** in nearly quantitative yield (96%, Scheme 1B); importantly, this product yield is highly reproducible, with 10 separate iterations producing yields between 92% and 97%. Furthermore, the specific order of the addition of the reagents is inconsequential to the outcome, demonstrating the robust nature of this methodology. We also investigated other common S(VI)–F reagents to determine if the highly reactive nature of the SIF reagent was required for this transformation. Replacing the SIF reagent with SulfoxFluor or PyFluor led to no conversion to fluoroformamidine, even when reaction times were extended to 24 h or when previously established literature conditions were employed.^18,19^

Finally, to expand the practicality of this pathway, a one-pot, multistep process was developed that started from benzaldehyde oxime (Scheme 1C). A reaction with *N*-chlorosuccinimide (NCS) in acetonitrile fully converts the aldoxime to the acid chloride oxime in just 15 min at room temperature; this is followed by the application of the conditions described above to produce **2a** in 70% yield (see SI for more details). While we ultimately chose to extend our investigation of this methodology using the acid chloride oximes as our starting point, the fact that this chemistry can be performed using aldoximes as the substrate, a molecule class that requires only one step from commercially available aldehydes, is worth noting.

With optimal and reproducible conditions determined, we aimed to elaborate on both tunable components of the fluoroformamidine products: the migrating R group and the nitrogen additive. Beginning with the former, a variety of acid chloride oximes were synthesized, stemming from inexpensive and abundant aldehydes (Figure 2, top). Using morpholine as the nitrogen component, a range of phenyl-derived R groups (**2a**–**2l**) were well-tolerated in this methodology. Several examples with electron-withdrawing (**2c**–**2f**) and -donating (**2g**, **2m**) *para*-substituents are shown in Figure 2, with isolated yields ranging from 71% to 90%. Unsurprisingly, *meta* substitution does not impact the formation of the fluoroformamidine (**2h**) while substrates with one *ortho* substituent are also well-tolerated (**2i**–**2k**). An acid chloride oxime stemming from 2,6-dimethylbenzaldehyde could be used in this methodology (**2l**), albeit with decreased conversion. Heterocyclic entities were also employed, with a 3-pyridyl motif and both the 2- and 3-position of thiophene derivatives migrating to the nitrogen to form novel fluoroformamidine products (**2m**–**2o**). Lastly, both alkenyl (**2p**) and alkyl (**2o**) groups led to fluoroformamidines, although the use of a migrating sp^3^-hybridized carbon diminished the yield of the fluorinated product compared to sp^2^ analogues.

**Figure 2:**
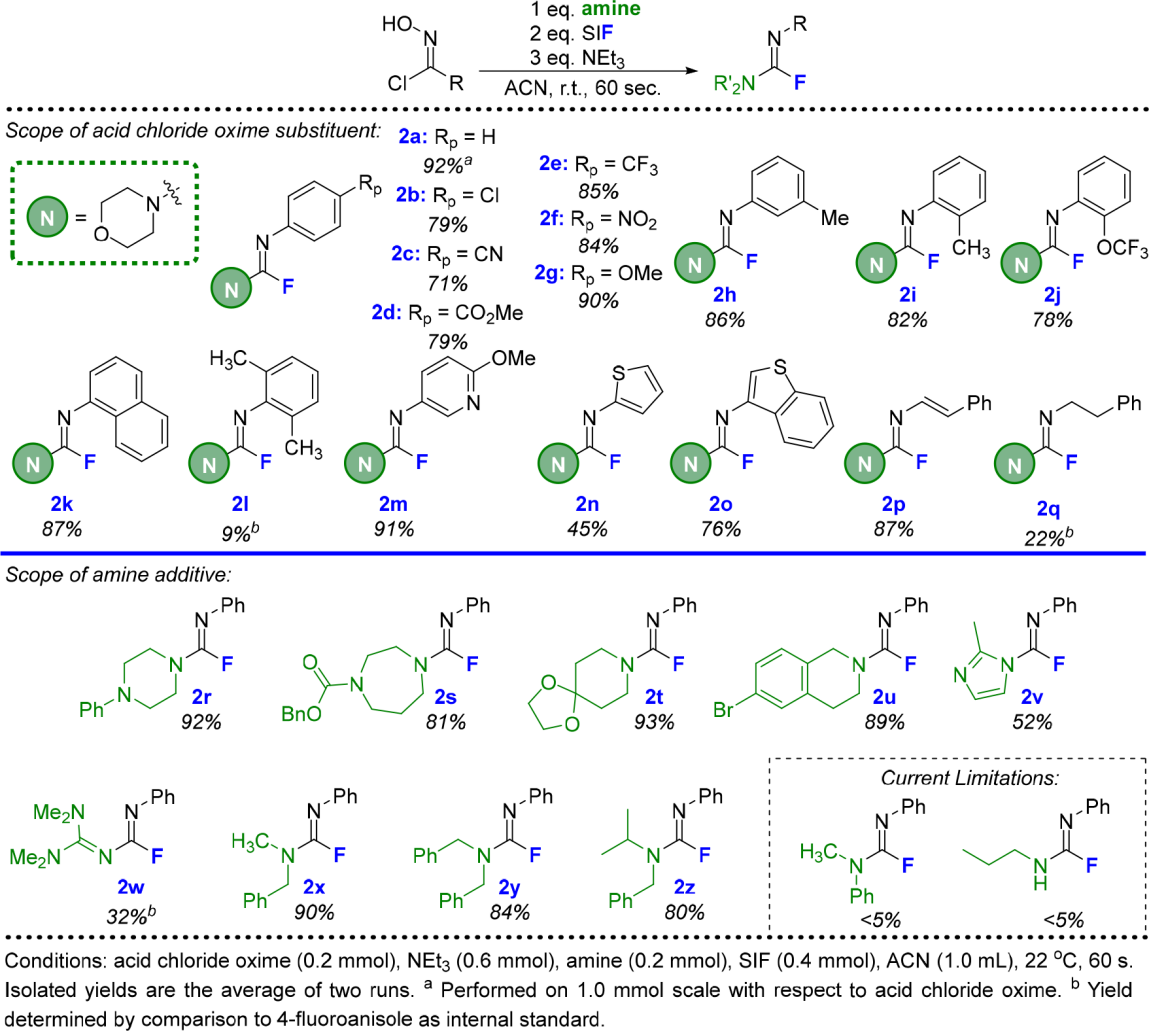
Substrate scope for the conversion of acid chloride oximes to fluoroformamidines.

Moving beyond morpholine, the nitrogen component could also be varied in combination with phenyl acid chloride oxime, NEt_3_, and SIF (**2r**–**2z**). A range of cyclic, secondary amines were highly effective, with substituted derivatives of piperazine (**2r**), homopiperazine (**2s**), and piperidine (**2t** and **2u**) providing fluoroformamidines in good to excellent isolated yields. The less nucleophilic 2-methylimidazole was also effective (**2v**), although a noticeable decrease in the isolated yield was attributed to poor conversion of the acid chloride oxime to the requisite amidoxime *in situ*. Another nitrogen-rich functional group, guanidine, could be combined with the fluoroformamidine motif using 1,1,3,3-tetramethylguanidine as the amine component (**2w**). Acyclic, secondary amines were also effective partners in this methodology, even with a sterically encumbered isopropylbenzylamine (**2z**). Current limitations center around two classes of amine nucleophiles: aniline derivatives and primary amines. In both cases, less than 5% of the fluoroformamidine product was observed in the crude ^19^F NMR. This can be attributed to the poor conversion of the acid chloride to the requisite amidoxime.

In the process of isolating more than 20 unique fluoroformamidine products, it became clear that these motifs are significantly more stable than their imidoyl fluoride counterparts. Fluoroformamidines show no signs of decomposition following standard workup procedures, including silica gel chromatography or aqueous washes. Furthermore, these compounds could be stored at room temperature on the benchtop for several months without any sign of degradation.

We next demonstrated the ability of fluoroformamidines to act as intermediates in the synthesis of other common nitrogen-rich functional groups: ureas and carbamimidates (Figure 3). It was also a central focus to generate these functional groups in a one-pot process thereby crafting protocols that directly convert acid chloride oximes to these valuable motifs. Beginning with urea derivatives, fluoroformamidine **2a** could be synthesized using the conditions described above and then converted to urea **3a** through the addition of water. Full conversion to the urea product required between 2 and 6 h at room temperature. Various acid chloride oxime R groups (**3a**–**3d**) and amines (**3e**–**3g**) could be incorporated into the urea derivatives, with good to excellent isolated yields in each case.

**Figure 3:**
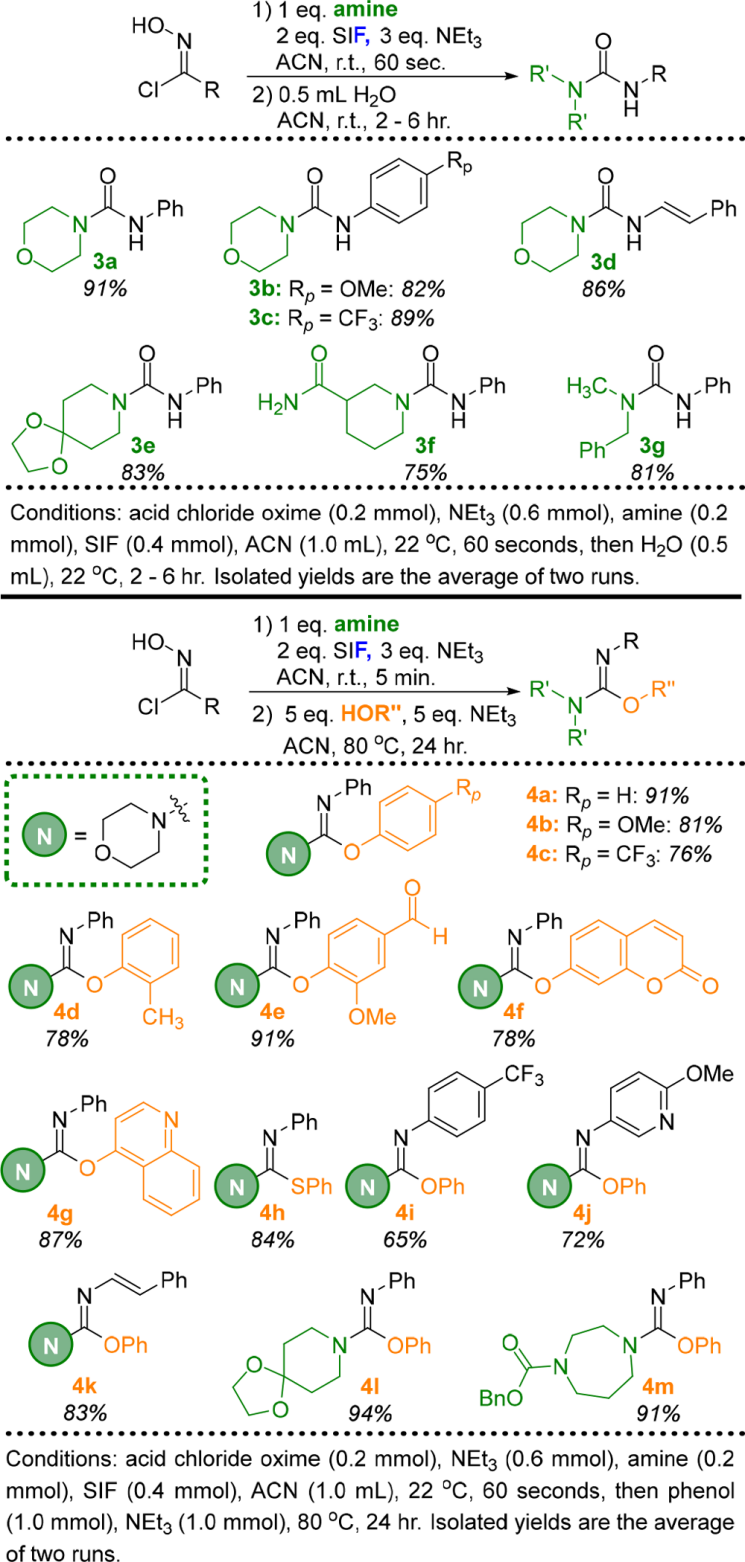
One-pot transformations of acid chloride oximes to ureas and carbamimidates via fluoroformamidines.

We next investigated the reactions of fluoroformamidines with phenols to produce carbamimidates. Several equivalents of phenol and NEt_3_ were required to achieve a 91% isolated yield of carbamimidate product **4a**, in addition to elevated temperatures (80 °C) and longer reaction times (24 h). With this one-pot methodology, we systematically varied each of the three components that constitute the carbamimidate product: phenol nucleophile, R group from the acid chloride oxime, and the amine component. Various phenols were well tolerated, with electron-donating (**4b**) and -withdrawing (**4c**) *para* substituents producing nearly identical yields. Similarly, *ortho* substitution on the phenol component (**4d** and **4e**) did not impact reactivity. Umbelliferone (**4f**) and 4-quinolinol (**4g**) also proved to be excellent candidates for this chemistry, while the 84% isolated yield of **4h** demonstrates that thiocarbamimidates are possible when thiophenol is employed. The R group of the acid chloride oxime could be altered to include a *para* substituent (**4i**) and a heterocyclic moiety (**4j**) in addition to an alkenyl substituent (**4k**). Finally, the amine component was expanded beyond morpholine to include additional functionality such as acetal (**4l**) and carbamate groups (**4m**).

Fluoroformamidines represent an underutilized, understudied functional group that combines multiple elements that hold significant relevance to drug design. The development of improved, modular syntheses will assist in bringing this interesting functionality to the fore. Using a highly reactive sulfur(VI)–fluoride reagent, we have successfully generated a novel protocol that employs the rearrangement of amidoximes toward the synthesis of more than 25 unique fluoroformamidines. This methodology has a multitude of benefits: (a) the requisite amidoximes are generated *in situ* from easily accessible acid chloride oximes; (b) a high tolerance for both the R group on the oxime and the amine additive leads to a plethora of possible combinations; (c) rapid and efficient reactivity produces excellent isolated yields of fluoroformamidines in just minutes at room temperature. Additionally, this protocol is conducive to further manipulations, providing an outlet for other nitrogen-rich moieties such as ureas and carbamimidates with future directions aiming to expand into the modular synthesis of guanidines. Overall, this methodology provides a more direct and efficient approach to fluoroformamidines, aimed at expanding the influence of this intriguing functional group.

## Data Availability

The data underlying this study are available in the published article and its Supporting Information.

## Supporting Information Available

Experimental procedures, characterization data, and NMR spectra (PDF)
