# Supplementary material for: Expanded Access to Fluoroformamidines via a Modular Synthetic Pathway
Source: Org Lett. 2024 Feb 7;26(6):1277–81. doi: 10.1021/acs.orglett.4c00131 (PMC10877594; doi:10.1021/acs.orglett.4c00131)
Supplement: Supplementary file 1 — ol4c00131_si_001.pdf [file ol4c00131_si_001.pdf]

Supporting Information for:

Expanded Access to Fluoroformamidines via a Modular Synthetic Pathway

James A. Vogel[‡], Kirya F. Miller[‡], Eunjeong Shin[‡], Jenna Krussman[‡], Patrick R. Melvin*[‡]

[‡] Department of Chemistry, Bryn Mawr College, Bryn Mawr, Pennsylvania 19010

Corresponding Author Email: prmelvin@brynmawr.edu

Table of Contents:

1. General Information – S3
2. Methods and Materials – S3
3. Synthesis of sulfone iminium fluoride reagent – S4
4. General procedures for the synthesis of aldoximes, acid chloride oximes and amidoximes – S5
5. Representation of proposed rearrangement mechanism – S8
6. Optimization and reproducibility experiments – S9
7. Synthesis of fluoroformamidine from aldoxime substrate – S14
8. Substrate scope for the formation of fluoroformamidines from acid chloride oximes – S15
9. Conversion of acid chloride oximes to urea derivatives via fluoroformamidines – S29
10. Conversion of acid chloride oximes to carbamimidate derivatives via fluoroformamidines – S33
11. NMR spectra of isolated products – S40
12. References – S112

1. General Information

NMR spectra were obtained on a Bruker 400 MHz (400.52 MHz for ^{1}H; 376.87 MHz for ^{19}F; 100.71 MHz for ^{13}C). ^{1}H and ^{13}C chemical shifts are reported in parts per million (ppm) relative the residual solvent peak ($CDCl_3$: ^{1}H: δ = 7.26 ppm, ^{13}C: δ = 77.16 ppm. ^{19}F NMR spectra are referenced based on the internal standard 4-fluoroanisole. ^{1}H and ^{19}F multiplicities are reported as follows: singlet (s), broad singlet (br), doublet (d), triplet (t), quartet (q), pentet (pent) multiplet (m), sextet (sext), septet (sept), doublet of doublets (dd), doublet of triplets (dt), triplet of doublets (td), doublet of doublet of doublets (ddd), doublet of doublet of triplets (ddt). Coupling constants (*J*) are reported in Hz. HRMS data reported for new compounds was done using ESI-TOF.

2. Methods and Materials

All reactions reported herein were performed without the exclusion of moisture or air. No dry solvents were used in any reactions for the synthesis of the sulfone iminium fluoride reagent or in the rearrangement reactions which employ the SIF.

Commercial reagents and solvents were used as received unless otherwise noted. Thiophenol, sulfuryl chloride, acetic acid, benzylamine, methyl trifluoromethanesulfonate, benzaldehyde, 4-chlorobenzaldehyde, 4-cyanobenzaldehyde, 4-trifluoromethylbenzaldehyde, 4-nitrobenzaldehyde, 4-methoxybenzaldehyde, 2-methylbenzaldehyde, 1-naphthylcarboxaldehyde, 2,6-dimethylbenzaldehyde, 1-phenylpiperizine, 1,4-dioxa-8-azaspiro[4.5]decane, 6-bromo-1,2,3,4-tetrahydroisoquinoline, 2-methylimidazole, *N*-methylbenzylamine, *N*-isopropylbenzylamine, phenol, 4-methoxyphenol, 4-trifluoromethylphenol, vanillin were purchased from Sigma Aldrich. 1,4-diazepane-1-carboxylate, 1,1,3,3-tetramethylguanidine, dibenzylamine, 2-methylphenol, umbelliferone, 4-quinolinol were purchased from Acros Organics. $CDCl_3$ was purchased from Cambridge Isotope Laboratories and used as is. Solvents were purchased from Pharmco and used as is.

3. Synthesis of sulfone iminium fluoride reagent

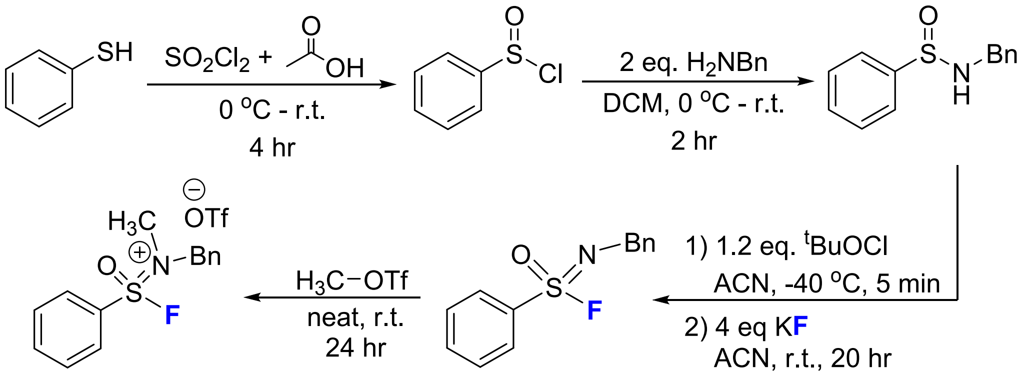

The following synthesis is modified from *Org. Lett.* **2022**, *24*, 5962.[1]

To a 100 mL round bottom flask, thiophenol (10.25 mL, 100 mmol) and acetic acid (5.75 mL, 100 mmol) were added with a stir bar and placed in an ice bath. Sulfuryl chloride (17.85 ml, 220 mmol) was added dropwise to the reaction at 0 °C. Vigorous bubbling was observed as well as a color change to orange-red. After the addition was complete, the reaction was stirred at room temperature for 4 hours, during which time bubbling continued. After 4 hours, acetyl chloride and excess sulfuryl chloride were removed under reduced pressure in a bath no higher than 10 °C. The product was isolated as a bright orange liquid and used without further purification.

To a 1000 mL round bottom flask, sulfinyl chloride (16.0 g, 0.10 mol) was added with a stir bar. DCM (400 mL) was added and the flask was placed in an ice bath. Once cooled, benzylamine (21.4 g, 0.20 mol) dissolved in DCM (100 mL) was added slowly to the reaction. An immediate white precipitate appeared along with a change to a colorless solution. The reaction was then stirred at room temperature for 2 hours. At this time, the reaction was filtered through a pad of celite followed by washing with H_2O (300 mL X 2). The organic layer was dried over Na_2SO_4, filtered and the solvent was removed under reduced pressure to yield a white solid (21.7 g, 94% yield). The sulfinamide product was used in subsequent reactions without any further purification.

To a 250 mL round bottom flask, *N*-benzyl benzenesulfinamide (6.94 g, 30 mmol) was added with acetonitrile (100 mL). The flask was then set in a dry ice / acetonitrile bath (-40 °C) and allowed to cool for 5 minutes. *Tert*-butyl hypochlorite (3.89 g, 36 mmol, 1.2 equivalents) was dissolved in acetonitrile (10 mL) and added to the reaction flask slowly over the course of 10 minutes. The reaction was allowed to slowly warm to room temperature at which time potassium fluoride (6.96 g, 120 mmol, 4 equivalents) was added to the reaction. The reaction was then stirred for 20 hours at room temperature. At this time, the reaction was filtered through a pad of celite and the solvent was removed under reduced pressure. The crude sulfonimidoyl fluoride product was purified by silica gel column chromatography using hexane / ethyl acetate (0% → 20% gradient, 40 g silica gel column) as eluent. Following purification, the *N*-benzyl benzenesulfonimidoyl fluoride was isolated as a pale yellow liquid (5.38 g, 72%).

In a well-ventilated fume hood under normal atmospheric conditions, *N*-benzyl benzenesulfonimidoyl fluoride (3.74 g, 15.0 mmol) was added with a flea stir bar to a 20 mL scintillation vial. Methyl trifluoromethanesulfonate (1.80 mL, 16.5 mmol. 1.1 equivalents) was added to the reaction at room temperature. The reaction was then stirred for 24 hours at room temperature. After this time, the crude reaction mixture was washed with hexane (10 mL, 5 times) in order to remove excess methyl trifluoromethanesulfonate. The sulfone iminium fluoride product (5.89 g, 98%) was then used without further purification.

4. General procedures for the synthesis of aldoximes, acid chloride oximes and amidoximes

General procedure for the synthesis of aldoximes:

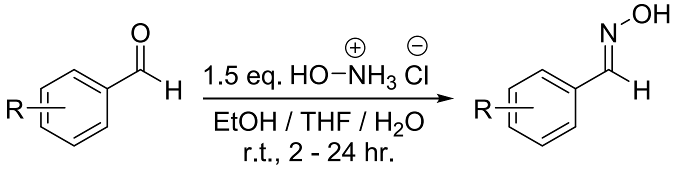

To a 250 mL round bottom flask, hydroxylamine hydrochloride (2.08 g, 30.0 mmol) was added along with a magnetic stir bar and subsequently dissolved in ethanol (30 mL) and water (5 mL). Aldehyde (20.0 mmol) was dissolved in THF (15 mL) and added to the reaction mixture at room temperature[1]. The reaction was monitored by GC/MS and the organic solvents were removed under reduced pressure when the starting material had been completely consumed (between 2 – 24 hours depending on aldehyde choice). The crude mixture was then extracted into dichloromethane and washed with water (3 X 100 mL). The organic layer was dried over Na_2SO_4, filtered and the product was isolated following the removal of the solvent under reduced pressure. The aldoxime product was used without any further purification.

[1]Aldehydes with *ortho* substitution were performed at 60 ℃ for 24 hours.

The following aldoxime products were synthesized using the above protocol:

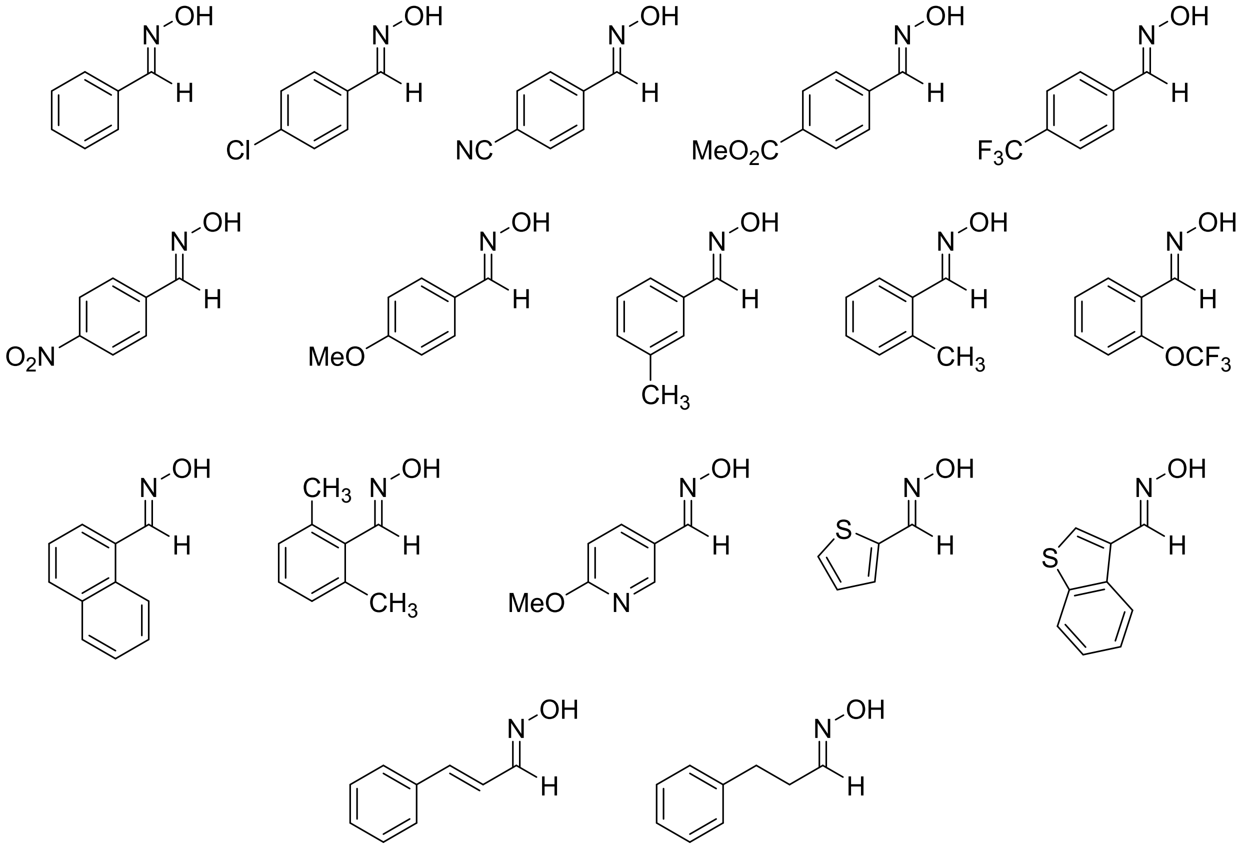

General procedure for the synthesis of acid chloride oximes:

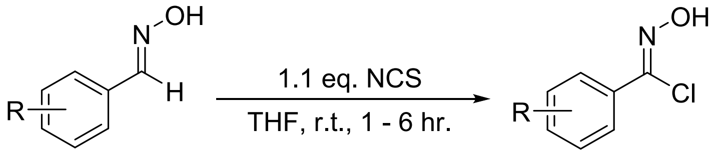

Aldoxime (10.0 mmol) was added to a 100 mL round bottom flask and dissolved in THF (20 mL). *N*-chlorosuccinimide (1.47 g, 11.0 mmol) was dissolved in THF (10 mL) and subsequently added to the reaction flask at room temperature. The reaction progress was monitored by GC / MS and the solvent was removed under reduced pressure following the consumption of the aldoxime starting material. The crude product mixture was dissolved in DCM (75 mL) and washed with H_2O (3 X 100 mL). The organic layer was dried over Na_2SO_4, filtered and the solvent was removed under reduced pressure. The acid chloride oxime products were purified by silica gel chromatography (24 g column) using hexane / ethyl acetate (0 → 50% gradient) as the eluent.

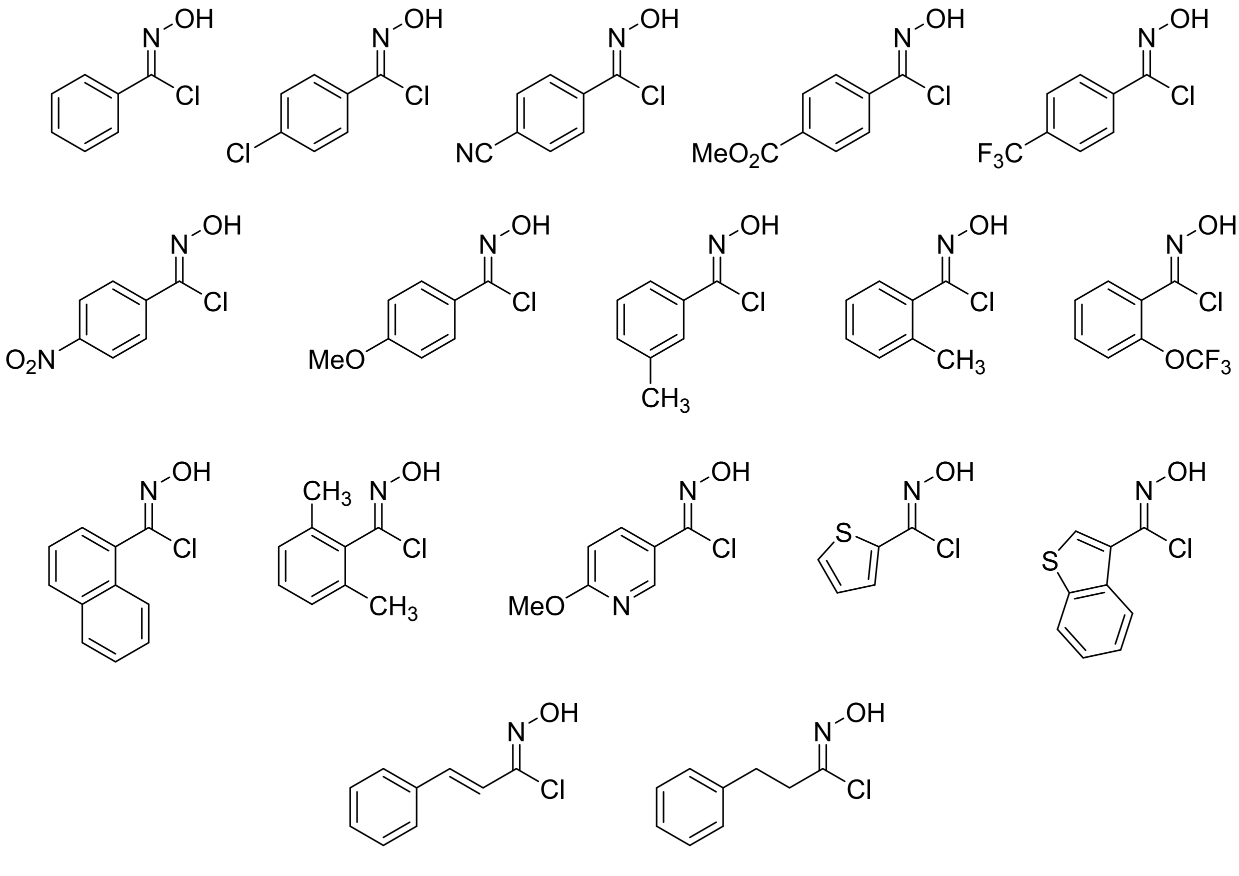

Synthesis of model amidoxime substrate, 4-morpholinylphenylmethanone oxime:

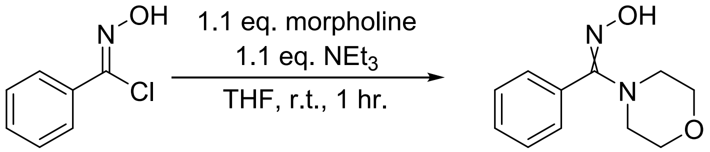

To a 100 mL round bottom flask, phenyl acid chloride oxime (0.778 g, 5.0 mmol) was added along with THF (10 mL). Morpholine (0.480 g, 5.5 mmol) and NEt_3 (0.555 g, 5.5 mmol) were combined in THF (10 mL) and then added to the reaction flask at room temperature. The reaction was allowed to stir for 1 hour and then the solvent was removed under reduced pressure. The crude reaction mixture was dissolved in DCM and washed with water (3 X 50 mL). The organic layer was dried over Na_2SO_4, filtered and the solvent was removed under reduced pressure. The amidoxime final product was used without further purification.

Notes on varying Z / E stereoisomer ratios

Freshly synthesized 4-morpholinylphenylmethanone oxime displayed E:Z isomerism ratios of 90:10 – 80:20. Upon leaving the isolated amidoxime for 1 week at room temperature on the bench-top, the E:Z isomerism ratios would shift to 60:40 – 50:50. The hydrogens labelled as A below were used to determine the approximate ratio of E:Z isomers with the corresponding chemical shift of those hydrogens also presented. ^1H NMR spectrum of freshly synthesized amidoxime is presented below for clarification (0.86 : 0.16 ratio).

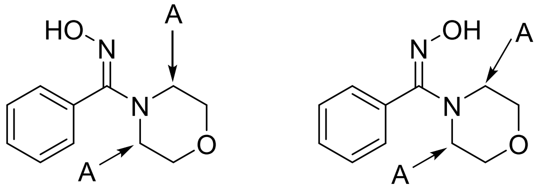

E Isomer
A: 3.40 ppm

Z Isomer
A: 3.03 ppm

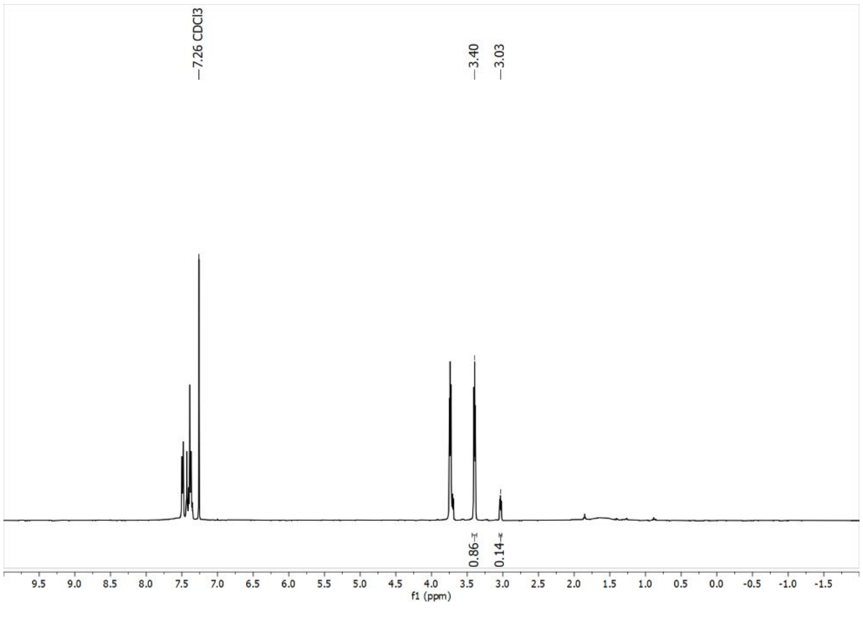

5. Representation of proposed rearrangement mechanism

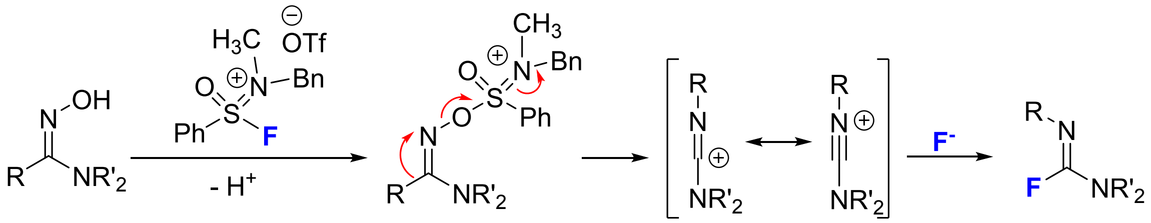

The proposed rearrangement begins through the substitution of the fluoride of the SIF rearrangement for the hydroxyl group of the amidoxime substrate. This generates the necessary leaving group out of the oxygen component. The R group trans to the leaving group migrates to the nitrogen as the SIF-generated leaving group departs. This leaves a resonance stabilized intermediate which can then be attacked by the remaining fluoride equivalent (from the initial SIF reagent) to produce the final fluoroformamidine.

6. Optimization and reproducibility experiments

a. Initial experiments using isolated amidoxime substrate

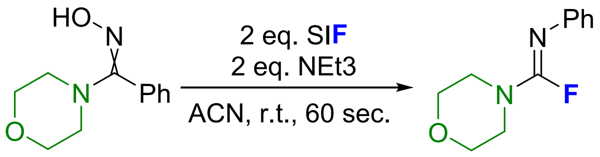

To an 8 mL vial equipped with a flea stir bar, 4-morpholinylphenylmethanone oxime (41.2 mg, 0.20 mmol) was added along with acetonitrile (0.5 mL). NEt_3 (20.0 mg, 0.40 mmol) dissolved in acetonitrile (0.25 mL) was added at room temperature followed by SIF (160 mg, 0.40 mmol) dissolved in acetonitrile (0.25 mL). The reaction was stirred for 60 seconds at room temperature at which time 4-fluoroanisole (0.2 mmol, 1.0 eq.) was added as an internal standard. The yield was determined by comparison of the product fluorine signal to that of the internal standard. A delay time of 10 seconds was used to ensure proper relaxation of all fluorine signals for integration purposes.

This process was repeated with various batches of isolated amidoxime, with the results of 5 iterations presented in Table S1 below.

The ^{19}F NMR spectra for iteration #2 is presented below the table as an example.

Table S1. Yields of 5 different iterations for the rearrangement of isolated amidoxime to fluoroformamidine

Iteration	Yield
1	76%
2	64%
3	44%
4	88%
5	74%

^{19}F NMR spectrum of crude reaction mixture from iteration #2:

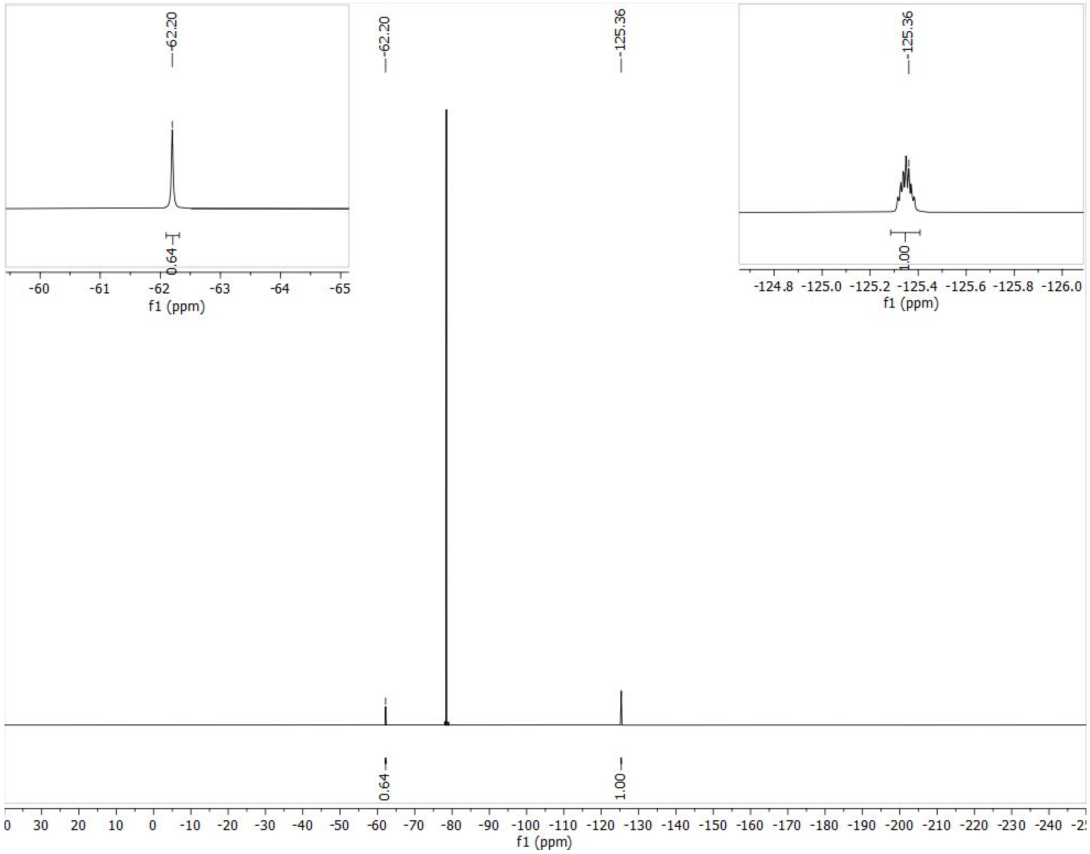

b. Generating amidoxime in situ *followed by rearrangement*

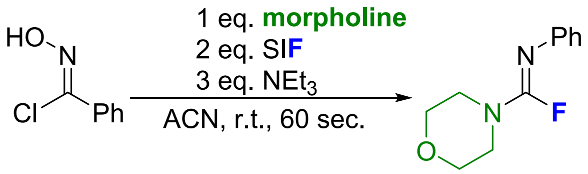

To an 8 mL vial equipped with a flea stir bar, phenyl acid chloride oxime (31.1 mg, 0.20 mmol) and NEt_3 (61.0 mg, 0.60 mmol) were added along with acetonitrile (0.5 mL). Morpholine (17.4 mg, 0.20 mmol) dissolved in acetonitrile (0.25 mL) was added at room temperature and stirred for 30 seconds before SIF (160 mg, 0.40 mmol) dissolved in acetonitrile (0.25 mL) completed the additions. Following the 60 seconds of reaction, 4-fluoroanisole (0.2 mmol, 1.0 eq.) was added as an internal standard. The yield was determined by comparison of the product fluorine signal to that of the internal standard. The recorded ^{19}F NMR spectrum is below which displays the yield presented in the manuscript (96%). A delay time of 10 seconds was used to ensure proper relaxation of all fluorine signals for integration purposes.

This process was repeated 10 times using various batches of phenyl acid chloride oxime. The results of those 10 iterations are reported below in Table S2.

Table S1. Yields of 10 different iterations for the formation of fluoroformamidine **2a** via an *in situ*-generation of amidoxime followed by rearrangement.

Iteration	Yield
1	96%
2	97%
3	92%
4	92%
5	94%
6	96%
7	97%
8	96%
9	92%
10	94%

^{19}F NMR spectrum of crude reaction mixture from iteration #1:

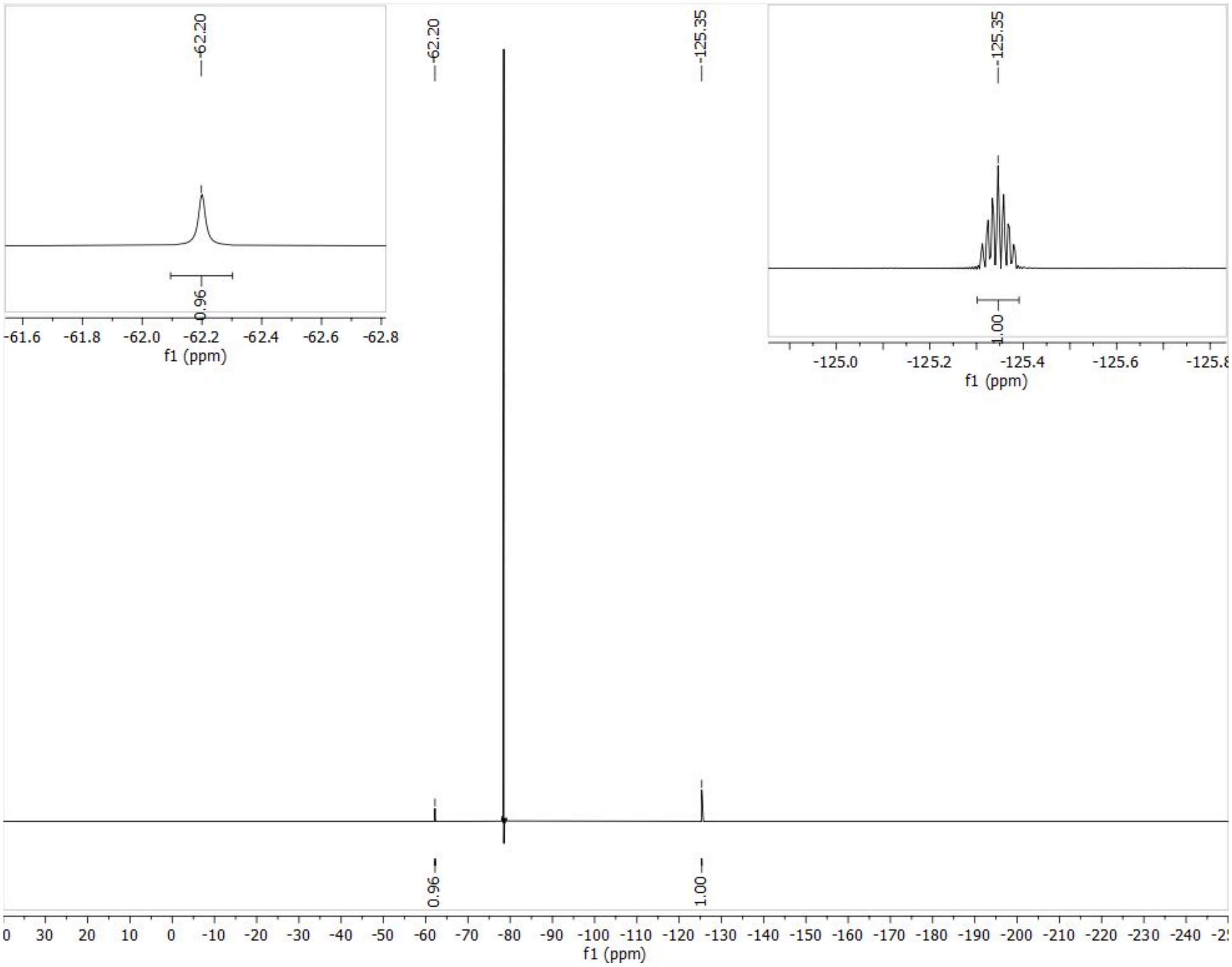

c. Replacing SIF reagent for other sulfur(VI) – fluoride reagents

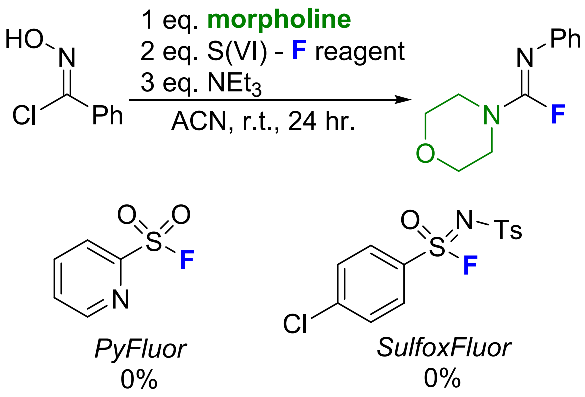

To an 8 mL vial equipped with a flea stir bar, phenyl acid chloride oxime (31.1 mg, 0.20 mmol) and NEt_3 (61.0 mg, 0.60 mmol) were added along with acetonitrile (0.5 mL). Morpholine (17.4 mg, 0.20 mmol) dissolved in acetonitrile (0.25 mL) was added at room temperature and stirred for 30 seconds before the sulfur(VI)-fluoride reagent (0.40 mmol) dissolved in acetonitrile (0.25 mL) completed the additions. Following the 24 hours of reaction time, 4-fluoroanisole (0.2 mmol, 1.0 eq.) was added as an internal standard. The yield (0%) was determined by comparing the product fluorine signal to that of the internal standard.

7. Synthesis of fluoroformamidine from aldoxime substrate

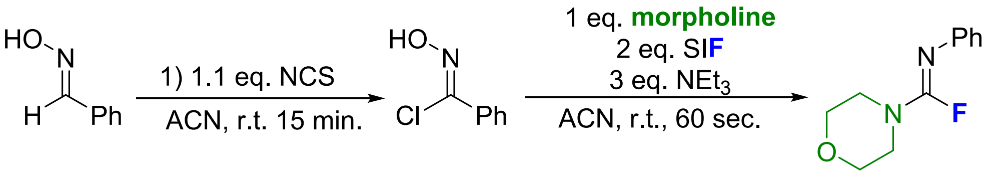

To an 8 mL vial equipped with a flea stir bar, benzaldehyde oxime (24.2 mg, 0.20 mmol) was added along with acetonitrile (0.3 mL). *N*-chlorosuccinimide (29.4 mg, 0.22 mmol) was added to the vial and the reaction was allowed to stir for 15 minutes at room temperature. Subsequently, NEt_3 (61.0 mg, 0.60 mmol) followed by morpholine (17.4 mg, 0.20 mmol) dissolved in acetonitrile (0.4 mL) was added at room temperature and stirred for 30 seconds. Finally, SIF (160 mg, 0.40 mmol) dissolved in acetonitrile (0.3 mL) was added and the reaction was stirred for 60 seconds at room temperature. Following the completion of the reaction, 4-fluoroanisole (25.2 mg, 0.20 mmol) was added as an internal standard and the ^{19}F NMR spectrum of the crude reaction mixture was recorded. Comparison of the product ^{19}F peak (-62.2 ppm) to that of the internal standard (-125.3 ppm) provided a yield of 70%. A delay time of 10 seconds was used to ensure proper relaxation of all fluorine signals for integration purposes.

^{19}F NMR spectrum of crude reaction mixture:

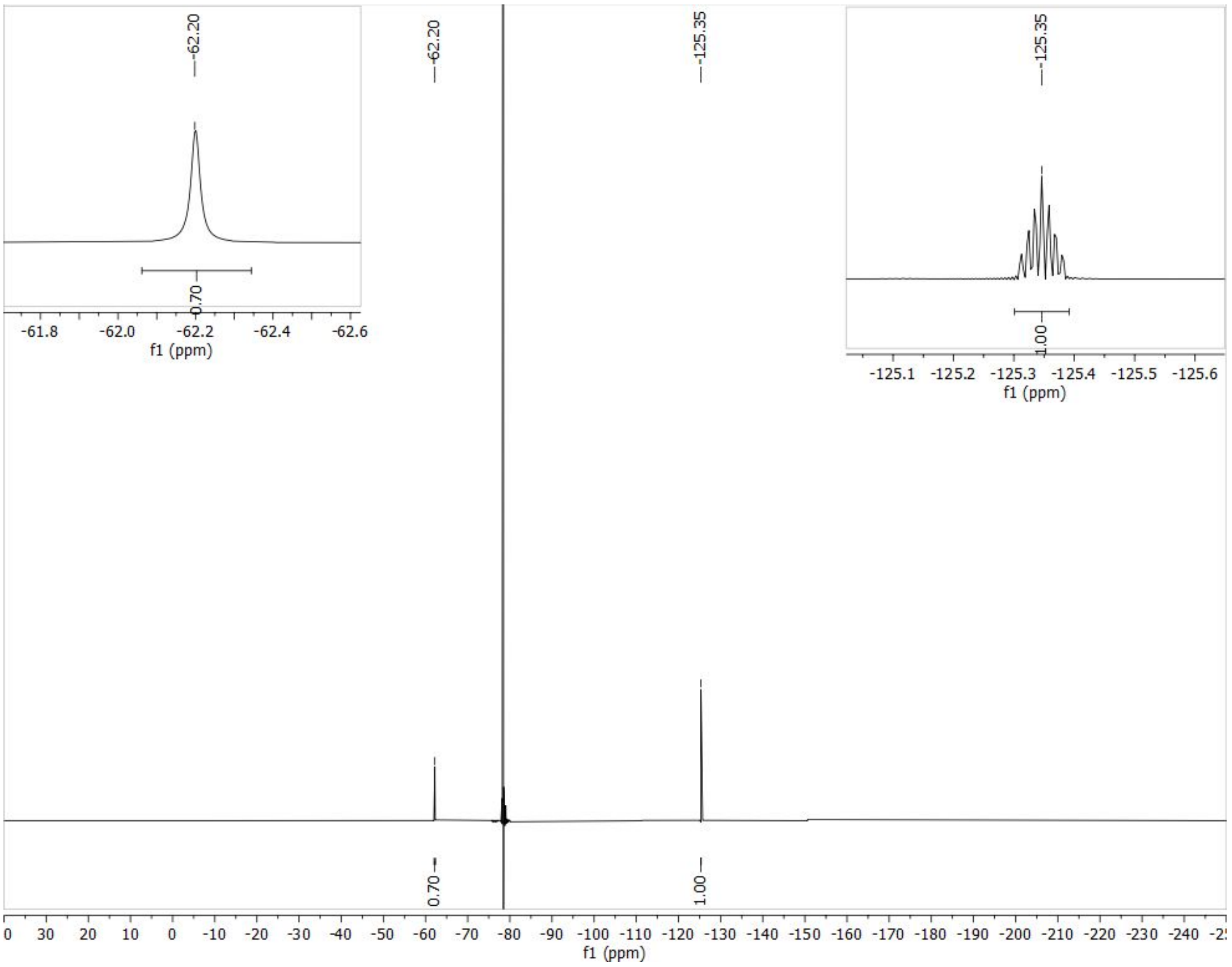

8. Substrate scope for the formation of fluoroformamidines from acid chloride oximes

General procedure for acid chloride oxime R group scope:

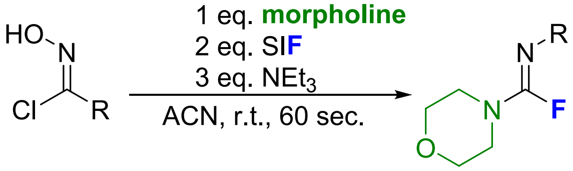

To an 8 mL vial equipped with a flea stir bar, acid chloride oxime (0.20 mmol) and NEt_3 (61.0 mg, 0.60 mmol) were added along with acetonitrile (0.5 mL). Morpholine (17.4 mg, 0.20 mmol) dissolved in acetonitrile (0.25 mL) was added at room temperature and stirred for 30 seconds before SIF (160 mg, 0.40 mmol) dissolved in acetonitrile (0.25 mL) completed the additions. The reaction was stirred for 60 seconds at room temperature at which time the acetonitrile was removed under reduced pressure. The crude reaction mixture was then purified via silica gel chromatography (12 g silica gel column), using DCM and ethyl acetate as the eluent (0% → 40% gradient).

(Z)-N-phenylmorpholine-4-carbimidoyl fluoride (2a)

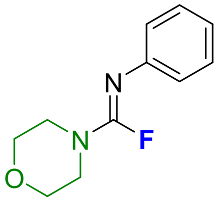

The reaction was performed using a scaled-up version of the general procedure described above with phenyl acid chloride oxime (155.6 mg, 1.0 mmol). Following silica gel chromatography (12 g silica gel column, 0% → 40% gradient using DCM and ethyl acetate), product **2a** was isolated as a white solid (194.0 mg, 93%). The isolated yield reported in the manuscript is the average of two runs (93% and 91% yield). The spectroscopic data were consistent with those previously published in the literature.[2]

^{1}H NMR (400 MHz, $CDCl_3$): 7.27 (t, J = 8.1 Hz, 2H), 6.99 – 7.05 (m, 3H), 3.76 (t, J = 5.0 Hz, 4H), 3.48 (t, J = 5.1 Hz, 4H) ppm.

^{13}C NMR (101 MHz, $CDCl_3$): 144.6 (d, $J_{C\text{-}F}$ = 311.4 Hz), 144.5 (d, $J_{C\text{-}F}$ = 10.3 Hz), 128.8, 123.4, 123.3, 77.5, 77.2, 76.8, 66.3, 45.3, 45.3, 45.3 ppm.

^{19}F NMR (376 MHz, $CDCl_3$): -61.6 (s) ppm.

(Z)-N-(4-chlorophenyl)morpholine-4-carbimidoyl fluoride (2b)

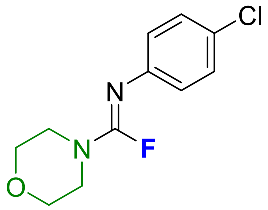

The reaction was performed using the general procedure described above with (4-chlorophenyl) acid chloride oxime (38.0 mg, 0.2 mmol). Following silica gel chromatography (12 g silica gel column, 0% → 40% gradient using DCM and ethyl acetate), product **2b** was isolated as a clear oil (39.3 mg, 81%). The isolated yield reported in the manuscript is the average of two runs (81% and 77% yield).

^{1}H NMR (400 MHz, $CDCl_3$): 7.21 (d, J = 8.8 Hz, 2H), 6.93 (d, J = 7.0 Hz, 2H), 3.75 (t, J = 5.1 Hz, 4H), 3.48 (t, J, = 5.0 Hz, 4H) ppm.

^{13}C NMR (101 MHz, $CDCl_3$): 144.6 (d, $J_{C\text{-}F}$ = 312.0 Hz), 143.1 (d, $J_{C\text{-}F}$ = 10.2 Hz), 128.8, 128.4, 124.8, 124.7, 66.3, 45.2 ppm.

^{19}F NMR (376 MHz, $CDCl_3$): -61.4 (s) ppm.

HRMS (ESI) m/z: [M + H]+ Calcd for $C_{11}H_{13}ClFN_2O$: 243.0700; Found 243.0705

(Z)-N-(4-cyanophenyl)morpholine-4-carbimidoyl fluoride (2c)

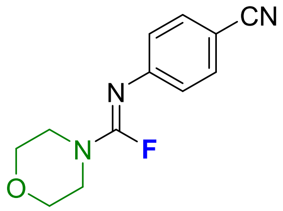

The reaction was performed using the general procedure described above with (4-cyanophenyl) acid chloride oxime (36.1 mg, 0.2 mmol). Following silica gel chromatography (12 g silica gel column, 0% → 40% gradient using DCM and ethyl acetate), product **2c** was isolated as a clear oil (34.5 mg, 74%). The isolated yield reported in the manuscript is the average of two runs (74% and 69% yield).

^{1}H NMR (400 MHz, $CDCl_3$): 7.52 (d, J = 8.5 Hz, 2H), 7.05 (d, J = 7.4 Hz, 2H), 3.76 (t, J = 5.1 Hz, 4H), 3.51 (t, J, = 5.0 Hz, 4H) ppm.

^{13}C NMR (101 MHz, $CDCl_3$): 149.1 (d, $J_{C\text{-}F}$ = 10.1 Hz), 145.1 (d, $J_{C\text{-}F}$ = 312.1 Hz), 133.0, 124.3, 119.6, 106.2, 66.2, 45.2 ppm.

^{19}F NMR (376 MHz, $CDCl_3$): -61.0 (s) ppm.

HRMS (ESI) m/z: [M + H]+ Calcd for $C_{12}H_{13}FN_3O$: 234.1042; Found 234.1040

methyl (Z)-4-((fluoro(morpholino)methylene)amino)benzoate (2d)

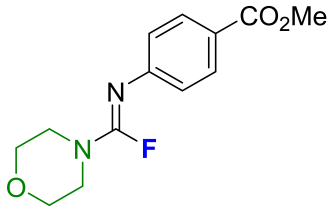

The reaction was performed using the general procedure described above with (4-(methylester)phenyl) acid chloride oxime (42.7 mg, 0.2 mmol). Following silica gel chromatography (12 g silica gel column, 0% → 40% gradient using DCM and ethyl acetate), product **2d** was isolated as a white solid (42.6 mg, 80%). The isolated yield reported in the manuscript is the average of two runs (80% and 78% yield).

^{1}H NMR (400 MHz, $CDCl_3$): 7.93 (d, *J* = 8.7 Hz, 2H), 7.03 (d, *J* = 8.7 Hz, 2H), 3.88 (s, 3H), 3.76 (t, *J* = 5.1 Hz, 4H), 3.51 (t, *J* = 5.0 Hz, 4H) ppm.

^{13}C NMR (101 MHz, $CDCl_3$): 167.2, 149.22 (d, $J_{C\text{-}F}$ = 10.1 Hz), 144.7 (d, $J_{C\text{-}F}$ = 311.9 Hz), 130.6, 124.9, 123.4, 66.3, 52.0, 45.2 ppm.

^{19}F NMR (376 MHz, $CDCl_3$): -61.0 (s) ppm.

HRMS (ESI) m/z: [M + H]+ Calcd for $C_{13}H_{16}FN_2O_3$: 267.1145; Found 267.1140

Melting point: 107 – 109 °C

(Z)-N-(4-(trifluoromethyl)phenyl)morpholine-4-carbimidoyl fluoride (2e)

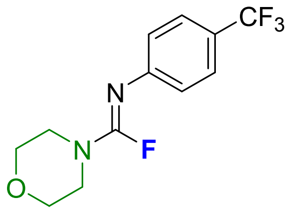

The reaction was performed using the general procedure described above with (4-(trifluoromethyl)phenyl) acid chloride oxime (44.7 mg, 0.2 mmol). Following silica gel chromatography (12 g silica gel column, 0% → 40% gradient using DCM and ethyl acetate), product **2e** was isolated as a clear oil (47.0 mg, 85%). The isolated yield reported in the manuscript is the average of two runs (85% and 85% yield).

^{1}H NMR (400 MHz, $CDCl_3$): 7.50 (d, *J* = 8.1 Hz, 2H), 7.07 (d, *J* = 8.2 Hz, 2H), 3.77 (t, *J* = 5.1 Hz, 4H), 3.51 (t, *J* = 5.1 Hz, 4H) ppm.

^{13}C NMR (101 MHz, $CDCl_3$): 148.0 (d, $J_{C\text{-}F}$ = 10.2 Hz), 144.8 (d, $J_{C\text{-}F}$ = 311.7 Hz), 129.3, 126.0 (q), 125.3, 125.0, 123.6, 66.3, 45.2 ppm.

^{19}F NMR (376 MHz, $CDCl_3$): -61.3 (s, 1F), -61.8 (s, 3F) ppm.

HRMS (ESI) m/z: [M + H]+ Calcd for $C_{12}H_{13}F_4N_2O$: 277.0964; Found 277.0963

(Z)-N-(4-nitrophenyl)morpholine-4-carbimidoyl fluoride (2f)

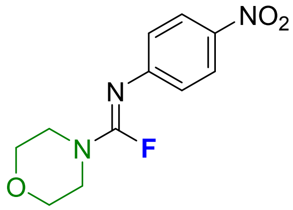

The reaction was performed using the general procedure described above with (4-nitrophenyl) acid chloride oxime (40.1 mg, 0.2 mmol). Following silica gel chromatography (12 g silica gel column, 0% → 40% gradient using DCM and ethyl acetate), product **2f** was isolated as a clear oil (43.0 mg, 85%). The isolated yield reported in the manuscript is the average of two runs (85% and 84% yield).

^{1}H NMR (400 MHz, $CDCl_3$): 8.13 (d, J = 7.9 Hz, 2H), 7.08 (d, J = 7.8 Hz, 2H), 3.78 (t, J = 5.1 Hz, 4H), 3.54 (t, J = 5.1 Hz, 4H) ppm.

^{13}C NMR (101 MHz, $CDCl_3$): 151.4 (d, $J_{C\text{-}F}$ = 10.1 Hz), 145.2 (d, $J_{C\text{-}F}$ = 312.0 Hz), 143.34, 124.8, 124.0, 66.2, 45.2 ppm.

^{19}F NMR (376 MHz, $CDCl_3$): -60.7 (s) ppm.

HRMS (ESI) m/z: [M + H]+ Calcd for $C_{11}H_{13}FN_3O_3$: 254.0941; Found 254.0941

(Z)-N-(4-methoxyphenyl)morpholine-4-carbimidoyl fluoride (2g)

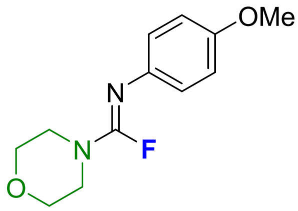

The reaction was performed using the general procedure described above with (4-methoxyphenyl) acid chloride oxime (37.1 mg, 0.2 mmol). Following silica gel chromatography (12 g silica gel column, 0% → 40% gradient using DCM and ethyl acetate), product **2g** was isolated as a clear oil (44.3 mg, 93%). The isolated yield reported in the manuscript is the average of two runs (93% and 87% yield).

^{1}H NMR (400 MHz, $CDCl_3$): 6.94 (d, J = 8.2 Hz, 2H), 6.83 (d, J = 8.0 Hz, 2H), 3.78 (s, 3H), 3.75 (t, J = 5.0 Hz, 4H), 3.46 (t, J = 5.1 Hz, 4H) ppm.

^{13}C NMR (101 MHz, $CDCl_3$): 155.8, 144.3 (d, $J_{C\text{-}F}$ = 311.7 Hz), 137.5 (d, $J_{C\text{-}F}$ = 10.4 Hz), 124.3, 114.1, 66.3, 55.6, 45.4 ppm.

^{19}F NMR (376 MHz, $CDCl_3$): -61.7 (s) ppm.

HRMS (ESI) m/z: [M + H]+ Calcd for $C_{12}H_{16}FN_2O_2$: 239.1196; Found 239.1201

(Z)-N-(*m*-tolyl)morpholine-4-carbimidoyl fluoride (2h)

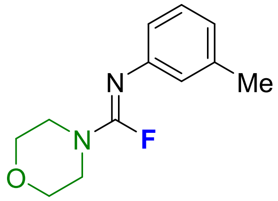

The reaction was performed using the general procedure described above with (3-methylphenyl) acid chloride oxime (34.0 mg, 0.2 mmol). Following silica gel chromatography (12 g silica gel column, 0% → 40% gradient using DCM and ethyl acetate), product **2h** was isolated as a clear oil (39.1 mg, 88%). The isolated yield reported in the manuscript is the average of two runs (88% and 85% yield).

^{1}H NMR (400 MHz, $CDCl_3$): 7.15 (t, *J* = Hz, 1H), 6.79 – 6.87 (m, 3H), 3.76 (t, *J* = 5.0 Hz, 4H), 3.47 (t, *J* = 5.1 Hz, 4H), 2.32 (s, 3H) ppm.

^{13}C NMR (101 MHz, $CDCl_3$): 144.5 (d, $J_{C\text{-}F}$ = 311.8 Hz), 144.4 (d, $J_{C\text{-}F}$ = 10.2 Hz), 142.8, 138.6, 128.6, 124.1, 120.3, 66.3, 45.3, 21.5 ppm.

^{19}F NMR (376 MHz, $CDCl_3$): -61.4 (s) ppm.

HRMS (ESI) m/z: [M + H]+ Calcd for $C_{12}H_{16}FN_2O$: 223.1247; Found 223.1246

(Z)-N-(*o*-tolyl)morpholine-4-carbimidoyl fluoride (2i)

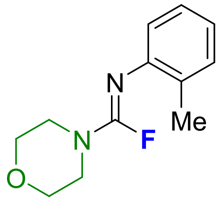

The reaction was performed using the general procedure described above with (2-methylphenyl) acid chloride oxime (34.0 mg, 0.2 mmol). Following silica gel chromatography (12 g silica gel column, 0% → 40% gradient using DCM and ethyl acetate), product **2i** was isolated as a clear oil (36.9 mg, 83%). The isolated yield reported in the manuscript is the average of two runs (83% and 80% yield).

^{1}H NMR (400 MHz, $CDCl_3$): 7.09 – 7.16 (m, 2H), 6.90 – 6.98 (m, 2H), 3.77 (t, *J* = 5.0 Hz, 4H), 3.49 (t, *J* = 5.1 Hz, 4H), 2.20 (s, 3H) ppm.

^{13}C NMR (101 MHz, $CDCl_3$): 143.7 (d, $J_{C\text{-}F}$ = 312.0 Hz), 143.34 (d, $J_{C\text{-}F}$ = 10.2 Hz), 130.9, 130.2, 126.2, 123.3, 122.6, 66.3, 45.5, 18.3 ppm.

^{19}F NMR (376 MHz, $CDCl_3$): -59.8 (s) ppm.

HRMS (ESI) m/z: [M + H]+ Calcd for $C_{12}H_{16}FN_2O$: 223.1247; Found 223.1246

(Z)-N-(2-(trifluoromethoxy)phenyl)morpholine-4-carbimidoyl fluoride (2j)

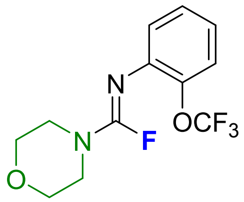

The reaction was performed using the general procedure described above with (2-(trifluoromethoxy)phenyl) acid chloride oxime (47.9 mg, 0.2 mmol). Following silica gel chromatography (12 g silica gel column, 0% → 40% gradient using DCM and ethyl acetate), product **2j** was isolated as a clear oil (46.8 mg, 80%). The isolated yield reported in the manuscript is the average of two runs (80% and 77% yield).

^{1}H NMR (400 MHz, $CDCl_3$): 7.17 – 7.22 (m, 2H), 7.02 – 7.06 (m, 2H), 3.76 (t, J = 5.0 Hz, 4H), 3.52 (t, J = 5.1 Hz, 4H) ppm.

^{13}C NMR (101 MHz, $CDCl_3$): 143.2 (d, $J_{C\text{-}F}$ = 311.8 Hz), 141.8 (d, $J_{C\text{-}F}$ = 10.1 Hz), 138.1, 127.3, 125.1, 123.8, 122.1, 66.3, 45.4 ppm.

^{19}F NMR (376 MHz, $CDCl_3$): -57.6 (s, 3F), -60.0 (s, 1F) ppm.

HRMS (ESI) m/z: [M + H]+ Calcd for $C_{12}H_{13}F_4N_2O_2$: 293.0913; Found 293.0920

(Z)-N-(naphthalen-1-yl)morpholine-4-carbimidoyl fluoride (2k)

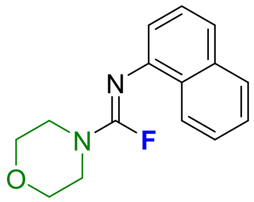

The reaction was performed using the general procedure described above with (1-naphthyl) acid chloride oxime (41.1 mg, 0.2 mmol). Following silica gel chromatography (12 g silica gel column, 0% → 40% gradient using DCM and ethyl acetate), product **2k** was isolated as a white solid (45.0 mg, 87%). The isolated yield reported in the manuscript is the average of two runs (87% and 87% yield).

^{1}H NMR (400 MHz, $CDCl_3$): 8.09 – 8.12 (m, 1H), 7.80 – 7.83 (m, 1H), 7.55 (d, J = 8.3 Hz, 1H), 7.44 – 7.48 (m, 2H), 7.39 (t, J = 7.3 Hz, 1H), 7.09 (d, J = 5.7 Hz, 1H), 3.82 (t, J = 5.2 Hz, 4H), 3.61 (t, J = 5.1 Hz, 4H) ppm.
^{13}C NMR (101 MHz, $CDCl_3$): 145.0 (d, $J_{C\text{-}F}$ = 311.6 Hz), 141.1 (d, $J_{C\text{-}F}$ = 10.2 Hz), 134.7, 129.4, 128.3, 126.3, 126.2, 125.7, 124.2, 123.6, 118.3, 118.3, 66.7, 45.8 ppm.

^{19}F NMR (376 MHz, $CDCl_3$): -60.6 (s) ppm.

HRMS (ESI) m/z: [M + H]+ Calcd for $C_{15}H_{16}FN_2O$: 259.1246; Found 259.1247

Melting point: 64 – 66 ℃

(Z)-N-(2,6-dimethylphenyl)morpholine-4-carbimidoyl fluoride (2l)

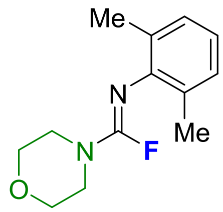

The reaction was performed using an adjusted version of the general procedure described above with (2,6-dimethylphenyl) acid chloride oxime (36.7 mg, 0.2 mmol). Following the 60 seconds of reaction, 4-fluoroanisole (0.2 mmol, 1.0 eq.) was added as an internal standard. The yield was determined by comparison of the product fluorine signal to that of the internal standard. The ^{19}F NMR yield reported in the manuscript is the average of two runs (9% and 9% yield).

^{19}F NMR (376 MHz, $CDCl_3$): -55.7 (s) ppm.

(Z)-N-(6-methoxypyridin-3-yl)morpholine-4-carbimidoyl fluoride (2m)

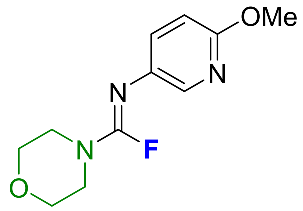

The reaction was performed using the general procedure described above with (6-methoxy-3-pyridyl) acid chloride oxime (37.3 mg, 0.2 mmol). Following silica gel chromatography (12 g silica gel column, 0% → 40% gradient using DCM and ethyl acetate), product **2m** was isolated as a clear oil (44.1 mg, 92%). The isolated yield reported in the manuscript is the average of two runs (92% and 90% yield).

^{1}H NMR (400 MHz, $CDCl_3$): 7.88 (s, 1H), 7.29 (dd, J = 4.7, 1.4 Hz, 1H), 6.64 (d, J = 9.4 Hz, 1H), 3.90 (s, 3H), 3.75 (t, J = 5.1 Hz, 4H), 3.48 (t, J = 5.1 Hz, 4H) ppm.

^{13}C NMR (101 MHz, $CDCl_3$): 177.3, 160.4, 145.1 (d, $J_{C\text{-}F}$ = 311.7 Hz), 141.0, 134.9 (d, $J_{C\text{-}F}$ = 10.0 Hz), 134.4, 110.4, 66.3, 53.6, 45.3 ppm.

^{19}F NMR (376 MHz, $CDCl_3$): -60.8 (s) ppm.

HRMS (ESI) m/z: [M + H]+ Calcd for $C_{11}H_{15}FN_3O_2$: 240.1148; Found 240.1152

(Z)-N-(thiophen-2-yl)morpholine-4-carbimidoyl fluoride (2n)

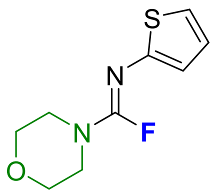

The reaction was performed using the general procedure described above with (2-thiophenyl) acid chloride oxime (32.3 mg, 0.2 mmol). Following silica gel chromatography (12 g silica gel column, 0% → 40% gradient using DCM and ethyl acetate), product **2n** was isolated as a clear oil (20.6 mg, 48%). The isolated yield reported in the manuscript is the average of two runs (48% and 43% yield).

^{1}H NMR (400 MHz, $CDCl_3$): 6.80 – 6.86 (m, 2H), 6.65 (dd, J = 2.0, 1.5 Hz, 1H), 3.75 (t, J = 5.1 Hz, 4H), 3.50 (t, J = 5.1 Hz, 4H) ppm.

^{13}C NMR (101 MHz, $CDCl_3$): 146.8 (d, J_{C-F} = 10.4 Hz), 144.8 (d, J_{C-F} = 311.8 Hz), 125.4, 118.8, 118.3, 66.3, 45.3 ppm.

^{19}F NMR (376 MHz, $CDCl_3$): -58.8 (s) ppm.

HRMS (ESI) m/z: [M + H]+ Calcd for $C_9H_{12}FN_2OS$: 215.0654; Found 215.0649

(Z)-N-(benzo[b]thiophen-3-yl)morpholine-4-carbimidoyl fluoride (2o)

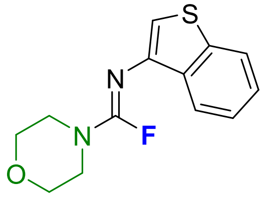

The reaction was performed using the general procedure described above with (3-benzothiophene) acid chloride oxime (42.3 mg, 0.2 mmol). Following silica gel chromatography (12 g silica gel column, 0% → 40% gradient using DCM and ethyl acetate), product **2o** was isolated as a clear oil (40.7 mg, 77%). The isolated yield reported in the manuscript is the average of two runs (77% and 75% yield).

^{1}H NMR (400 MHz, $CDCl_3$): 7.83 (d, J = 7.3 Hz, 1H), 7.80 (d, J = 7.2 Hz, 1H), 7.32 – 7.39 (m, 2H), 6.94 (d, J = 2.6 Hz, 1H), 3.80 (t, J = 5.2 Hz, 4H), 3.58 (t, J = 5.1 Hz, 4H) ppm.

^{13}C NMR (101 MHz, $CDCl_3$): 146.2 (d, J_{C-F} = 312.1 Hz), 138.1, 137.0, 136.3 (d, J_{C-F} = 10.3 Hz), 124.6, 123.8, 122.7, 122.0, 111.5, 66.3, 45.4 ppm.

^{19}F NMR (376 MHz, $CDCl_3$): -56.7 (s) ppm.

HRMS (ESI) m/z: [M + H]+ Calcd for $C_{13}H_{14}FN_2OS$: 265.0811; Found 265.0806

(Z)-N-((E)-styryl)morpholine-4-carbimidoyl fluoride (2p)

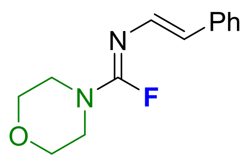

The reaction was performed using the general procedure described above with (transcinnamaldehyde) acid chloride oxime (36.3 mg, 0.2 mmol). Following silica gel chromatography (12 g silica gel column, 0% → 40% gradient using DCM and ethyl acetate), product **2p** was isolated as a clear oil (41.7 mg, 89%). The isolated yield reported in the manuscript is the average of two runs (89% and 85% yield).

^{1}H NMR (400 MHz, $CDCl_3$): 7.43 (d, J = 13.6 Hz, 1H), 7.25 – 7.35 (m, 5H), 7.15 (t, J = 8.7 Hz, 1H), 6.35 (d, J = 7.6 Hz, 1H), 3.73 (t, J = 5.2 Hz, 4H), 3.48 (t, J = 5.1 Hz, 4H) ppm.

^{13}C NMR (101 MHz, $CDCl_3$): 146.8 (d, $J_{C\text{-}F}$ = 312.3 Hz), 137.6, 129.8 (d, $J_{C\text{-}F}$ = 10.2 Hz), 128.6, 126.5, 125.9, 122.3, 66.3, 45.2 ppm.

^{19}F NMR (376 MHz, $CDCl_3$): -63.0 (s) ppm.

HRMS (ESI) m/z: [M + H]+ Calcd for $C_{13}H_{16}FN_2O$: 235.1246; Found 235.1250

(Z)-N-phenethylmorpholine-4-carbimidoyl fluoride (2q)

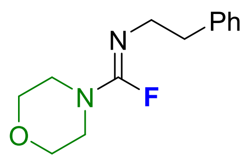

The reaction was performed using an adjusted version of the general procedure described above with (2-phenylethyl) acid chloride oxime (36.7 mg, 0.2 mmol). Following the 60 seconds of reaction, 4-fluoroanisole (0.2 mmol, 1.0 eq.) was added as an internal standard. The yield was determined by comparison of the product fluorine signal to that of the internal standard. The ^{19}F NMR yield reported in the manuscript is the average of two runs (23% and 20% yield).

^{19}F NMR (376 MHz, $CDCl_3$): -61.2 (s) ppm.

General procedure for amine scope:

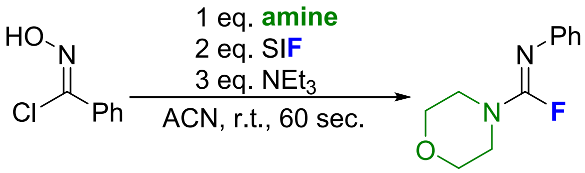

To an 8 mL vial equipped with a flea stir bar, phenyl acid chloride oxime (31.1 mg, 0.20 mmol) and NEt_3 (61.0 mg, 0.60 mmol) were added along with acetonitrile (0.5 mL). Amine (0.20 mmol) dissolved in acetonitrile (0.25 mL) was added at room temperature and stirred for 30 seconds before SIF (160 mg, 0.40 mmol) dissolved in acetonitrile (0.25 mL) completed the additions. The reaction was stirred for 60 seconds at room temperature at which time the acetonitrile was removed under reduced pressure. The crude reaction mixture was then purified via silica gel chromatography (12 g silica gel column), using DCM and ethyl acetate as the eluent (0% → 40% gradient) or hexane and ethyl acetate (0% → 100% gradient).

(Z)-N,4-diphenylpiperazine-1-carbimidoyl fluoride (2r)

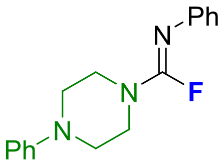

The reaction was performed using the general procedure described above with 1-phenylpiperizine (32.4 mg, 0.2 mmol). Following silica gel chromatography (12 g silica gel column, 0% → 40% gradient using DCM and ethyl acetate), product **2r** was isolated as a colorless oil (52.1 mg, 92%). The isolated yield reported in the manuscript is the average of two runs (92% and 92% yield).

^{1}H NMR (400 MHz, $CDCl_3$): 7.25 – 7.33 (m, 4H), 6.91 – 7.06 (m, 6H), 3.66 (t, J = 5.2 Hz, 4H), 3.25 (t, J = 5.4 Hz, 4H) ppm.

^{13}C NMR (101 MHz, $CDCl_3$): 147.9 (d, $J_{C\text{-}F}$ = 313.4 Hz), 146.0, 142.9 (d, $J_{C\text{-}F}$ = 10.5 Hz), 129.4, 128.8, 123.5, 123.2, 120.8, 117.0, 49.2, 45.1 ppm.

^{19}F NMR (376 MHz, $CDCl_3$): -61.0 (s) ppm.

HRMS (ESI) m/z: [M + H]+ Calcd for $C_{17}H_{19}FN_3$: 284.1563; Found 284.1560

benzyl (Z)-4-(fluoro(phenylimino)methyl)-1,4-diazepane-1-carboxylate (2s)

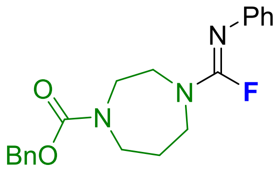

The reaction was performed using the general procedure described above with benzyl 1,4-diazepane-1-carboxylate (46.9 mg, 0.2 mmol). Following silica gel chromatography (12 g silica gel column, 0% → 40%

gradient using DCM and ethyl acetate), product **2s** was isolated as a colorless oil (59.7 mg, 84%). The isolated yield reported in the manuscript is the average of two runs (84% and 79% yield).

^{1}H NMR (400 MHz, CDCl$_3$): 7.32 – 7.38 (m, 5H), 7.25 (t, J = 6.7 Hz, 2H), 6.96 – 7.04 (m, 3H), 5.17 (d, J = 6.8 Hz, 2H), 3.48 – 3.68 (m, 8H), 1.96 (sept, J = 6.4 Hz, 2H) ppm.

^{13}C NMR (101 MHz, CDCl$_3$): 156.0 (d, J_{C-F} = 25.3 Hz), 144.1 (d, J_{C-F} = 311.7 Hz), 144.7 (d, J_{C-F} = 10.7 Hz), 136.8, 128.8, 128.7, 128.2, 128.1, 128.0, 123.6, 123.0, 67.4, 49.3, 48.3, 47.2, 46.6, 26.9 ppm.

^{19}F NMR (376 MHz, CDCl$_3$): -61.1 (s) ppm.

HRMS (ESI) m/z: [M + H]+ Calcd for $C_{20}H_{23}FN_3O_2$: 356.1774; Found 356.1780

(Z)-N-phenyl-1,4-dioxa-8-azaspiro[4.5]decane-8-carbimidoyl fluoride (2t)

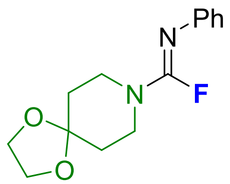

The reaction was performed using the general procedure described above with 1,4-dioxa-8-azaspiro[4.5]decane (28.6 mg, 0.2 mmol). Following silica gel chromatography (12 g silica gel column, 0% → 40% gradient using hexane and ethyl acetate), product **2t** was isolated as a colorless oil (49.7 mg, 94%). The isolated yield reported in the manuscript is the average of two runs (94% and 90% yield).

^{1}H NMR (400 MHz, CDCl$_3$): 7.26 (t, J = 8.1 Hz, 2H), 6.99 – 7.02 (m, 3H), 4.00 (s, 4H), 3.60 (t, J = 5.5 Hz, 4H), 1.78 (t, J = 5.6 Hz, 4H) ppm.

^{13}C NMR (101 MHz, CDCl$_3$): 144.4 (d, J_{C-F} = 312.0 Hz), 144.9 (d, J_{C-F} = 10.4 Hz), 128.8, 123.5, 123.0, 106.9, 64.6, 43.5, 34.5 ppm.

^{19}F NMR (376 MHz, CDCl$_3$): -61.6 (s) ppm.

HRMS (ESI) m/z: [M + H]+ Calcd for $C_{14}H_{18}FN_2O_2$: 265.1352; Found 265.1357

(Z)-6-bromo-N-phenyl-3,4-dihydroisoquinoline-2(1H)-carbimidoyl fluoride (2u)

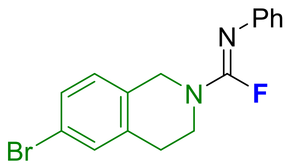

The reaction was performed using the general procedure described above with 6-bromo-1,2,3,4-tetrahydroisoquinoline (42.4 mg, 0.2 mmol). Following silica gel chromatography (12 g silica gel column, 0% → 40% gradient using hexane and ethyl acetate), product **2u** was isolated as a colorless oil (60.0 mg, 90%). The isolated yield reported in the manuscript is the average of two runs (90% and 88% yield).

^{1}H NMR (400 MHz, CDCl$_3$): 7.33 – 7.36 (m, 2H), 7.29, 7.29 (m, 2H), 7.00 – 7.05 (m, 4H), 4.61 (s, 2H), 3.72 (t, J = 5.6 Hz, 2H), 2.93 (t, J = 5.5 Hz, 2H) ppm.

^{13}C NMR (101 MHz, CDCl$_3$): 144.3 (d, $J_{C\text{-}F}$ = 312.0 Hz), 136.5, 131.7, 129.8, 128.8, 128.1, 123.5, 123.2, 120.5, 46.6, 42.2, 28.5 ppm.

^{19}F NMR (376 MHz, CDCl$_3$): -60.4 (s) ppm.

HRMS (ESI) m/z: [M + H]+ Calcd for $C_{16}H_{15}BrFN_2$: 333.0402; Found 333.0400

(Z)-2-methyl-N-phenyl-1H-imidazole-1-carbimidoyl fluoride (2v)

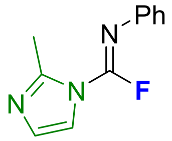

The reaction was performed using the general procedure described above with 2-methylimidazole (16.4 mg, 0.2 mmol). Following silica gel chromatography (12 g silica gel column, 0% → 40% gradient using DCM and ethyl acetate), product **2v** was isolated as a colorless oil (22.3 mg, 55%). The isolated yield reported in the manuscript is the average of two runs (55% and 49% yield).

^{1}H NMR (400 MHz, CDCl$_3$): 7.36 – 7.50 (m, 3H), 7.18 – 7.24 (m, 2H), 7.01 (d, J = 9.8 Hz, 1H), 6.96 (d, J = 3.8 Hz, 1H), 2.72 (s, 3H) ppm.

^{13}C NMR (101 MHz, CDCl$_3$): 144.5 (d, $J_{C\text{-}F}$ = 311.0 Hz), 140.9 (d, $J_{C\text{-}F}$ = 10.6 Hz), 129.2, 127.5, 125.8, 123.4, 120.9, 118.0, 17.6 ppm.

^{19}F NMR (376 MHz, CDCl$_3$): -45.3 (s) ppm.

HRMS (ESI) m/z: [M + H]+ Calcd for $C_{11}H_{11}FN_3$: 204.0937; Found 204.0942

(Z)-N-(bis(dimethylamino)methylene)-N'-phenylcarbamimidic fluoride (2w)

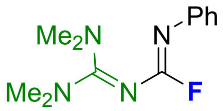

The reaction was performed using an adjusted version of the general procedure described above with 1,1,3,3-tetramethylguanidine (23.0 mg, 0.2 mmol). Following the 60 seconds of reaction, 4-fluoroanisole (0.2 mmol, 1.0 eq.) was added as an internal standard. The yield was determined by comparison of the product fluorine signal to that of the internal standard. The ^{19}F NMR yield reported in the manuscript is the average of two runs (33% and 30% yield).

^{19}F NMR (376 MHz, CDCl$_3$): -61.1 (s) ppm.

(Z)-N-benzyl-N-methyl-N'-phenylcarbamimidic fluoride (2x)

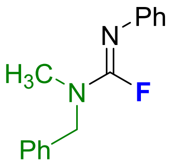

The reaction was performed using the general procedure described above with *N*-methylbenzylamine (24.2 mg, 0.2 mmol). Following silica gel chromatography (12 g silica gel column, 0% → 40% gradient using hexane and ethyl acetate), product **2x** was isolated as a colorless oil (44.6 mg, 92%). The isolated yield reported in the manuscript is the average of two runs (92% and 89% yield).

^{1}H NMR (400 MHz, CDCl$_3$): 7.27 – 7.40 (m, 7H), 7.01 – 7.05 (m, 3H), 4.54 (s, 2H), 2.97 (s, 3H) ppm.

^{13}C NMR (101 MHz, CDCl$_3$): 145.5 (d, $J_{C\text{-}F}$ = 312.0 Hz), 145.0 (d, $J_{C\text{-}F}$ = 10.6 Hz), 136.9, 128.9, 128.8, 127.9, 127.8, 123.6, 122.9, 53.4, 35.1 ppm.

^{19}F NMR (376 MHz, CDCl$_3$): -60.5 (s) ppm.

HRMS (ESI) m/z: [M + H]+ Calcd for $C_{15}H_{16}FN_2$: 243.1297; Found 243.1291

(Z)-N,N-dibenzyl-N'-phenylcarbamimidic fluoride (2y)

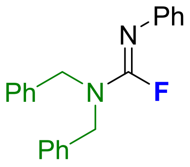

The reaction was performed using the general procedure described above with dibenzylamine (39.5 mg, 0.2 mmol). Following silica gel chromatography (12 g silica gel column, 0% → 40% gradient using hexane and ethyl acetate), product **2y** was isolated as a white solid (54.1 mg, 85%). The isolated yield reported in the manuscript is the average of two runs (85% and 83% yield).

^{1}H NMR (400 MHz, CDCl$_3$): 7.25 – 7.39 (m 13H), 7.01 – 7.08 (m, 3H), 4.49 (s, 4H) ppm.

^{13}C NMR (101 MHz, CDCl$_3$): 145.5 (d, $J_{C\text{-}F}$ = 311.9 Hz), 145.0 (d, $J_{C\text{-}F}$ = 10.6 Hz), 136.9, 128.9, 128.8, 128.1, 127.8, 123.7, 123.1, 50.2 ppm.

^{19}F NMR (376 MHz, CDCl$_3$): -60.4 (s) ppm.

HRMS (ESI) m/z: [M + H]+ Calcd for $C_{21}H_{20}FN_2$: 319.1610; Found 319.1603

Melting point: 43 – 46 °C

(Z)-N-benzyl-N-isopropyl-N'-phenylcarbamimidic fluoride (2z)

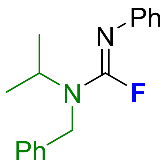

The reaction was performed using the general procedure described above with *N*-isopropylbenzylamine (29.8 mg, 0.2 mmol). Following silica gel chromatography (12 g silica gel column, 0% → 40% gradient using hexane and ethyl acetate), product **2z** was isolated as a colorless oil (43.2 mg, 80%). The isolated yield reported in the manuscript is the average of two runs (80% and 79% yield).

^{1}H NMR (400 MHz, CDCl$_3$): 7.32 – 7.36 (m, 4H), 7.23 – 7.28 (m, 3H), 6.98 – 7.02 (m, 3H), 4.50 (s, 2H), 4.22 (sept, J = 8.6 Hz, 1H), 1.23 (d, J = 5.5 Hz, 6H) ppm.

^{13}C NMR (101 MHz, CDCl$_3$): 145.4 (d, $J_{C\text{-}F}$ = 311.7 Hz), 145.1 (d, $J_{C\text{-}F}$ = 10.9 Hz), 139.1, 128.7, 128.6, 127.2, 127.2, 123.6, 122.8, 49.8, 47.7, 20.7 ppm.

^{19}F NMR (376 MHz, CDCl$_3$): -60.2 (s) ppm.

HRMS (ESI) m/z: [M + H]+ Calcd for $C_{17}H_{20}FN_2$: 271.1610; Found 271.1605

9. Conversion of acid chloride oximes to urea derivatives via fluoroformamidines

General procedure:

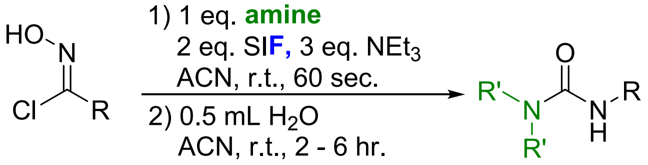

To an 8 mL vial equipped with a flea stir bar, acid chloride oxime (0.20 mmol) and NEt_3 (61.0 mg, 0.60 mmol) were added along with acetonitrile (0.5 mL). Amine (0.20 mmol) dissolved in acetonitrile (0.25 mL) was added at room temperature and stirred for 30 seconds before SIF (160 mg, 0.40 mmol) dissolved in acetonitrile (0.25 mL) completed the additions. The reaction was stirred for 60 seconds at room temperature at which time water (0.5 mL) was added and the reaction was allowed to stir for 2 – 6 hours, tracked by the disappearance of the fluoroformamidine intermediate by ^{19}F NMR spectroscopy. Following the completion of the reaction, the acetonitrile was removed under reduced pressure and the crude mixture was then extracted into DCM and washed with water (3 X 5 mL). The organic layer was dried over Na_2SO_4, filtered and the solvent was removed. The crude product was then purified by silica gel chromatography (12 gram silica gel column) using DCM / ethyl acetate as the eluent (0 → 40% gradient).

N-phenylmorpholine-4-carboxamide (3a)

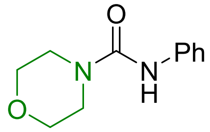

The reaction was performed using the general procedure above with phenyl acid chloride oxime (31.1 mg, 0.2 mmol) and morpholine (17.4 mg, 0.2 mmol) over 2 hours. Following silica gel chromatography (12 g silica gel column, 0% → 40% gradient using DCM and ethyl acetate), product **3a** was isolated as a white solid (38.0 mg, 93%). The isolated yield reported in the manuscript is the average of two runs (93% and 90% yield). The spectroscopic data were consistent with those previously published in the literature.[3]

^{1}H NMR (400 MHz, $CDCl_3$): 7.33 (d, J = 8.7 Hz, 2H), 7.28 (t, J = 9.0 Hz, 2H), 7.05 (t, J = 5.7 Hz, 1H), 6.43 (br, 1H), 3.72 (t, J = 5.2 Hz, 4H), 3.47 (t, J = 5.1 Hz, 4H) ppm.

^{13}C NMR (101 MHz, $CDCl_3$): 155.3, 138.8, 129.1, 123.5, 120.2, 66.6, 44.4 ppm.

N-(4-methoxyphenyl)morpholine-4-carboxamide (3b)

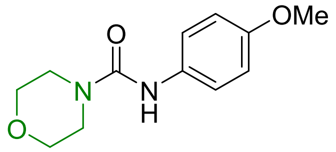

The reaction was performed using the general procedure above with (4-methoxyphenyl) acid chloride oxime (37.1 mg, 0.2 mmol) and morpholine (17.4 mg, 0.2 mmol) over 4 hours. Following silica gel chromatography (12 g silica gel column, 0% → 40% gradient using DCM and ethyl acetate), product **3b**

was isolated as a white solid (40.2 mg, 85%). The isolated yield reported in the manuscript is the average of two runs (85% and 80% yield). The spectroscopic data were consistent with those previously published in the literature.[3]

^{1}H NMR (400 MHz, CDCl$_3$): 7.22 (d, J = 6.6 Hz, 2H), 6.84 (d, J = 6.5 Hz, 2H), 6.32 (br, 1H), 3.78 (s, 3H), 3.71 (t, J = 5.1 Hz, 4H), 3.44 (t, J = 5.0 Hz, 4H) ppm.

^{13}C NMR (101 MHz, CDCl$_3$): 156.2, 155.8, 131.8, 122.7, 114.3, 66.6, 55.6, 44.4 ppm.

N-(4-(trifluoromethyl)phenyl)morpholine-4-carboxamide (3c)

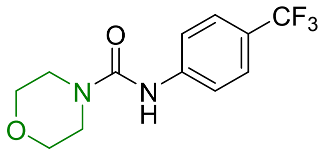

The reaction was performed using the general procedure above with (4-trifluoromethylphenyl) acid chloride oxime (44.7 mg, 0.2 mmol) and morpholine (17.4 mg, 0.2 mmol) over 2 hours. Following silica gel chromatography (12 g silica gel column, 0% → 40% gradient using DCM and ethyl acetate), product **3c** was isolated as a colorless oil (49.3 mg, 90%). The isolated yield reported in the manuscript is the average of two runs (90% and 88% yield). The spectroscopic data were consistent with those previously published in the literature.[4]

^{1}H NMR (400 MHz, CDCl$_3$): 7.51 (d, J = 8.8 Hz, 2H), 7.47 (d, J = 8.7 Hz, 2H), 6.66 (br, 1H), 3.72 (t, J = 5.2 Hz, 4H), 3.48 (t, J = 5.1 Hz, 4H) ppm.

^{13}C NMR (101 MHz, CDCl$_3$): 154.6, 142.1, 126.3 (q), 125.7, 124.9, 119.4, 66.5, 44.4 ppm.

^{19}F NMR (376 MHz, CDCl$_3$): -61.9 (s) ppm.

(E)-N-styrylmorpholine-4-carboxamide (3d)

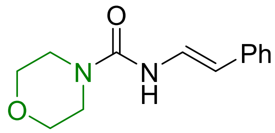

The reaction was performed using the general procedure above with (transcinnamaldehyde) acid chloride oxime (36.3 mg, 0.2 mmol) and morpholine (17.4 mg, 0.2 mmol) over 2 hours. Following silica gel chromatography (12 g silica gel column, 0% → 40% gradient using DCM and ethyl acetate), product **3d** was isolated as a colorless oil (40.0 mg, 86%). The isolated yield reported in the manuscript is the average of two runs (86% and 85% yield).

^{1}H NMR (400 MHz, CDCl$_3$): 7.49 (dd, J = 10.4, 4.1 Hz, 1H), 7.23 – 7.29 (m, 4H), 7.09 – 7.14 (m, 1H), 6.65 (d, J = 10.7 Hz, 1H), 5.96 (d, J = 7.5 Hz, 1H), 3.70 (t, J = 5.1 Hz, 4H), 3.44 (t, J = 5.0 Hz, 4H) ppm.

^{13}C NMR (101 MHz, CDCl$_3$): 154.1, 136.8, 128.8, 126.2, 125.3, 125.2, 109.8, 66.5, 44.2 ppm.

HRMS (ESI) m/z: [M + H]+ Calcd for $C_{13}H_{17}N_2O_2$: 233.1290; Found 233.1285

N-phenyl-1,4-dioxa-8-azaspiro[4.5]decane-8-carboxamide (3e)

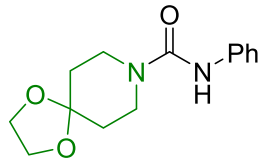

The reaction was performed using the general procedure above with phenyl acid chloride oxime (31.1 mg, 0.2 mmol) and 1,4-dioxa-8-azaspiro[4.5]decane (28.6 mg, 0.2 mmol) over 2 hours. Following silica gel chromatography (12 g silica gel column, 0% → 40% gradient using DCM and ethyl acetate), product **3e** was isolated as a colorless oil (44.6 mg, 85%). The isolated yield reported in the manuscript is the average of two runs (85% and 80% yield). The spectroscopic data were consistent with those previously published in the literature.[5]

^{1}H NMR (400 MHz, CDCl$_3$): 7.25 – 7.34 (m, 4H), 7.01 (t, J = 8.1 Hz, 1H), 6.50 (br, 1H), 3.98 (s, 4H), 3.57 (t, J = 5.3 Hz, 4H), 1.73 (t, J = 5.2 Hz, 4H) ppm.

^{13}C NMR (101 MHz, CDCl$_3$): 170.5, 154.9, 139.2, 129.7, 129.0, 128.6, 126.9, 123.2, 120.1, 107.0, 64.6, 42.6, 35.0 ppm.

N^1-phenylpiperidine-1,3-dicarboxamide (3f)

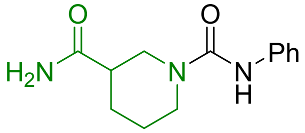

The reaction was performed using the general procedure above with phenyl acid chloride oxime (31.1 mg, 0.2 mmol) and piperidine-3-carboxamide (25.6 mg, 0.2 mmol) over 2 hours. Following silica gel chromatography (12 g silica gel column, 0% → 40% gradient using DCM and ethyl acetate), product **3f** was isolated as a colorless oil (38.1 mg, 77%). The isolated yield reported in the manuscript is the average of two runs (77% and 74% yield).

^{1}H NMR (400 MHz, CDCl$_3$): 7.27 – 7.35 (m, 4H), 7.05 (tt, J = 7.1, 1.7 Hz, 1H), 6.48 (br, 1H), 3.91 (dd, J = 9.9, 3.7 Hz, 1H), 3.47 – 3.56 (m, 2H), 3.33 – 3.39 (m, 1H), 2.78 (sept, J = 3.9 Hz, 1H), 2.03 – 2.09 (m, 1H), 1.82 – 1.95 (m, 2H), 1.61 – 1.66 (m, 1H) ppm.

^{13}C NMR (101 MHz, CDCl$_3$): 154.9, 138.7, 129.1, 123.7, 120.3, 46.4, 45.2, 28.0, 27.5, 23.4 ppm.

HRMS (ESI) m/z: [M + H]+ Calcd for $C_{13}H_{18}N_3O_2$: 248.1399; Found 248.1403

1-benzyl-1-methyl-3-phenylurea (3g)

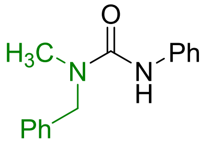

The reaction was performed using the general procedure above with phenyl acid chloride oxime (31.1 mg, 0.2 mmol) and *N*-methylbenzylamine (24.2 mg, 0.2 mmol) over 6 hours. Following silica gel chromatography (12 g silica gel column, 0% → 40% gradient using DCM and ethyl acetate), product **3g** was isolated as a white solid (39.4 mg, 82%). The isolated yield reported in the manuscript is the average of two runs (82% and 80% yield). The spectroscopic data were consistent with those previously published in the literature.[6]

^{1}H NMR (400 MHz, $CDCl_3$): 7.22 – 7.36 (m, 10H), 7.00 (t, *J* = 7.2 Hz, 1H), 6.39 (br, 1H), 4.56 (s, 2H), 3.00 (s, 3H) ppm.

^{13}C NMR (101 MHz, $CDCl_3$): 155.8, 139.2, 137.6, 129.0, 128.9, 127.7, 127.4, 123.2, 120.0, 52.5, 34.9 ppm.

10. Conversion of acid chloride oximes to carbamimidate derivatives via fluoroformamidines

General procedure:

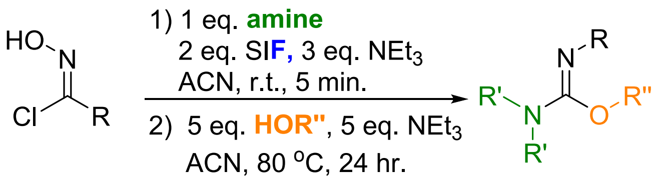

To an 8 mL vial equipped with a flea stir bar, acid chloride oxime (0.20 mmol) and NEt_3 (61.0 mg, 0.60 mmol) were added along with acetonitrile (0.5 mL). Amine (0.20 mmol) dissolved in acetonitrile (0.25 mL) was added at room temperature and stirred for 30 seconds before SIF (160 mg, 0.40 mmol) dissolved in acetonitrile (0.25 mL) completed the additions. The reaction was stirred for 60 seconds at room temperature at which time phenol (1.0 mmol) and NEt_3 (101 mg, 1.0 mmol) were added in acetonitrile (0.5 mL) and the reaction was allowed to stir for 24 hours at 80 ℃ using a hot plate + PIE-BLOCK. Following the completion of the reaction, the acetonitrile was removed under reduced pressure and the crude mixture was then purified by silica gel chromatography (12 gram silica gel column) using DCM / ethyl acetate as the eluent (0 → 100% gradient).

phenyl (Z)-N-phenylmorpholine-4-carbimidate (4a)

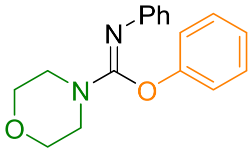

The reaction was performed using the general procedure above with phenyl acid chloride oxime (31.1 mg, 0.2 mmol), morpholine (17.4 mg, 0.2 mmol) and phenol (94.1 mg, 1.0 mmol). Following silica gel chromatography (12 g silica gel column, 0% → 100% gradient using DCM and ethyl acetate), product **4a** was isolated as a clear oil (51.9 mg, 92%). The isolated yield reported in the manuscript is the average of two runs (92% and 90% yield).

^{1}H NMR (400 MHz, $CDCl_3$): 7.16 (t, J = 8.8 Hz, 2H), 7.06 (t, J = 8.7 Hz, 2H), 6.93 (t, J = 7.3 Hz, 1H), 6.81 – 6.86 (m, 5H), 3.72 (t, J = 5.0 Hz, 4H), 3.53 (t, J = 5.1 Hz, 4H) ppm.

^{13}C NMR (101 MHz, $CDCl_3$): 154.45, 149.0, 146.7, 129.6, 128.5, 123.1, 122.9, 122.4, 116.6, 66.7, 45.8 ppm.

HRMS (ESI) m/z: [M + H]+ Calcd for $C_{17}H_{19}N_2O_2$: 283.1446; Found 283.1451

4-methoxyphenyl (Z)-N-phenylmorpholine-4-carbimidate (4b)

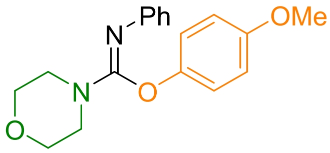

The reaction was performed using the general procedure above with phenyl acid chloride oxime (31.1 mg, 0.2 mmol), morpholine (17.4 mg, 0.2 mmol) and 4-methoxyphenol (124.1 mg, 1.0 mmol). Following silica gel chromatography (12 g silica gel column, 0% → 100% gradient using DCM and ethyl acetate), product **4b** was isolated as a clear oil (50.6 mg, 81%). The isolated yield reported in the manuscript is the average of two runs (81% and 80% yield).

^{1}H NMR (400 MHz, CDCl$_3$): 7.07 (t, *J* = 8.6 Hz, 2H), 6.80 – 6.85 (m, 3H) 6.75 (d, *J* = 9.0 Hz, 2H), 6.69 (d, *J* = 9.2 Hz, 2H), 3.71 (m, 7H), 3.52 (t, *J* = 5.2 Hz, 4H) ppm.

^{13}C NMR (101 MHz, CDCl$_3$): 155.3, 149.8, 148.4, 146.9, 128.5, 122.8, 122.3, 117.6, 114.6, 66.7, 55.7, 45.9 ppm. 313.1552

HRMS (ESI) m/z: [M + H]+ Calcd for $C_{18}H_{21}N_2O_3$: 313.1552; Found 313.1559

4-(trifluoromethyl)phenyl (Z)-N-phenylmorpholine-4-carbimidatecarbimidate (4c)

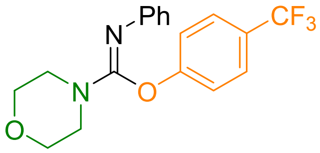

The reaction was performed using the general procedure above with phenyl acid chloride oxime (31.1 mg, 0.2 mmol), morpholine (17.4 mg, 0.2 mmol) and 4-trifluoromethylphenol (162.1 mg, 1.0 mmol). Following silica gel chromatography (12 g silica gel column, 0% → 100% gradient using DCM and ethyl acetate), product **4c** was isolated as a clear oil (54.6 mg, 78%). The isolated yield reported in the manuscript is the average of two runs (78% and 74% yield).

^{1}H NMR (400 MHz, CDCl$_3$): 7.42 (d, *J* = 9.1 Hz, 2H), 7.07 (t, *J* = 8.7 Hz, 2H), 6.94 (d, *J* = 9.0 Hz, 2H), 6.80 – 6.85 (m, 3H), 3.73 (t, *J* = 5.1 Hz, 4H), 3.53 (t, *J* = 5.0 Hz, 4H) ppm.

^{13}C NMR (101 MHz, CDCl$_3$): 156.7, 147.9, 146.2, 128.6, 127.1, 125.2, 122.8, 122.7, 119.9, 116.6, 66.6, 45.8 ppm.

^{19}F NMR (376 MHz, CDCl$_3$): -61.9 (s) ppm.

HRMS (ESI) m/z: [M + H]+ Calcd for $C_{18}H_{18}F_3N_2O_2$: 351.1320; Found 351.1313

***o*-tolyl (Z)-N-phenylmorpholine-4-carbimidate (4d)**

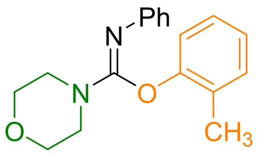

The reaction was performed using the general procedure above with phenyl acid chloride oxime (31.1 mg, 0.2 mmol), morpholine (17.4 mg, 0.2 mmol) and 2-methylphenol (108.1 mg, 1.0 mmol). Following silica gel chromatography (12 g silica gel column, 0% → 100% gradient using DCM and ethyl acetate), product

4d was isolated as a clear oil (47.4 mg, 80%). The isolated yield reported in the manuscript is the average of two runs (80% and 75% yield).

^{1}H NMR (400 MHz, CDCl$_3$): 6.77 – 7.06 (m, 9H), 3.73 (t, *J* = 5.2 Hz, 4H), 3.53 (t, *J* = 5.0 Hz, 4H), 2.08 (s, 3H) ppm.

^{13}C NMR (101 MHz, CDCl$_3$): 152.5, 149.3, 146.9, 1423.0, 131.2, 128.3, 126.7, 123.1, 122.7, 122.2, 115.3, 66.7, 45.8, 16.0 ppm.

HRMS (ESI) m/z: [M + H]+ Cald for $C_{18}H_{20}N_2O_2$: 296.1524; Found 296.1525

4-formyl-2-methoxyphenyl (Z)-N-phenylmorpholine-4-carbimidate (4e)

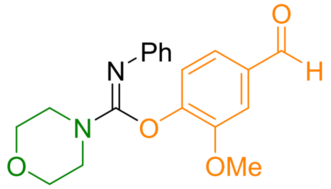

The reaction was performed using the general procedure above with phenyl acid chloride oxime (31.1 mg, 0.2 mmol), morpholine (17.4 mg, 0.2 mmol) and vanillin (152.1 mg, 1.0 mmol). Following silica gel chromatography (12 g silica gel column, 0% → 100% gradient using DCM and ethyl acetate), product **4e** was isolated as a clear oil (62.6 mg, 92%). The isolated yield reported in the manuscript is the average of two runs (92% and 90% yield).

^{1}H NMR (400 MHz, CDCl$_3$): 9.77 (s, 1H), 7.26 (d, *J* = 1.8 Hz, 1H), 7.18 (dd, *J* = 6.2, 1.9 Hz, 1H), 6.98 (t, *J* = 8.3 Hz, 2H), 6.89 (d, *J* = 8.2 Hz, 1H), 6.72 – 6.76 (m, 3H), 3.87 (s, 3H), 3.76 (t, *J* = 5.1 Hz, 4H), 3.55 (t, *J* = 5.1 Hz, 4H) ppm.

^{13}C NMR (101 MHz, CDCl$_3$): 190.9, 149.9, 148.6, 148.3, 146.5, 132.8, 128.3, 125.2, 122.6, 122.3, 119.1, 110.5, 66.6, 56.1, 46.1 ppm.

HRMS (ESI) m/z: [M + H]+ Calcd for $C_{19}H_{21}N_2O_4$: 341.1501; Found 341.1500

2-oxo-2*H*-chromen-7-yl (Z)-N-phenylmorpholine-4-carbimidate (4f)

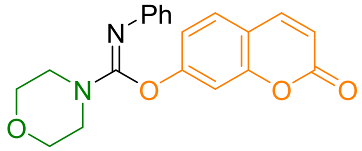

The reaction was performed using the general procedure above with phenyl acid chloride oxime (31.1 mg, 0.2 mmol), morpholine (17.4 mg, 0.2 mmol) and umbelliferone (162.1 mg, 1.0 mmol). Following silica gel chromatography (12 g silica gel column, 0% → 100% gradient using DCM and ethyl acetate), product **4f** was isolated as a clear oil (55.3 mg, 79%). The isolated yield reported in the manuscript is the average of two runs (79% and 77% yield).

^{1}H NMR (400 MHz, CDCl$_3$): 7.54 (d, *J* = 9.6 Hz, 1H), 7.28 (d, *J* = 9.5 Hz, 1H), 7.07 (t, *J* = 9.0 Hz, 2H), 6.77 – 6.85 (m 5H), 6.27 (d, *J* = 9.6 Hz, 1H), 3.74 (t, *J* = 5.1 Hz, 4H), 3.54 (t, *J* = 5.2 Hz, 4H) ppm.

^{13}C NMR (101 MHz, CDCl$_3$): 160.7, 156.9, 155.3, 147.4, 145.9, 143.0, 129.0, 128.6, 122.8, 122.8, 114.7, 114.4, 113.4, 104.7, 66.6, 45.9 ppm.

HRMS (ESI) m/z: [M + H]+ Calcd for $C_{20}H_{19}N_2O_4$: 351.1344; Found 351.1350

quinolin-4-yl (Z)-N-phenylmorpholine-4-carbimidate (4g)

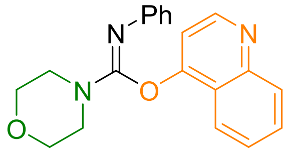

The reaction was performed using the general procedure above with phenyl acid chloride oxime (31.1 mg, 0.2 mmol), morpholine (17.4 mg, 0.2 mmol) and 4-quinolinol (145.2 mg, 1.0 mmol). Following silica gel chromatography (12 g silica gel column, 0% → 100% gradient using DCM and ethyl acetate), product **4g** was isolated as a clear oil (58.7 mg, 88%). The isolated yield reported in the manuscript is the average of two runs (88% and 87% yield).

^{1}H NMR (400 MHz, CDCl$_3$): 8.89 (dd, *J* = 2.5, 1.7 Hz, 1H), 8.03 (dd, *J* = 6.6, 1.7 Hz, 1H), 7.33 – 7.78 (m, 2H), 7.23 (t, *J* = 7.8 Hz, 1H), 7.09 (d, *J* = 6.5 Hz, 1H), 6.86 (t, *J* = 8.5 Hz, 2H), 6.71 (d, *J* = 8.6 Hz, 2H), 6.62 (t, *J* = 7.3 Hz, 1H), 3.77 (t, *J* = 5.3 Hz, 4H), 3.66 (t, *J* = 5.2 Hz, 4H) ppm.

^{13}C NMR (101 MHz, CDCl$_3$): 150.0, 149.6, 146.9, 139.6, 135.9, 129.3, 128.1, 126.2, 122.9, 122.7, 121.8, 121.7, 116.3, 66.7, 46.2 ppm.

HRMS (ESI) m/z: [M + H]+ Calcd for $C_{20}H_{20}N_3O_2$: 334.1555; Found 334.1559

phenyl (Z)-N-phenylmorpholine-4-carbimidothioate (4h)

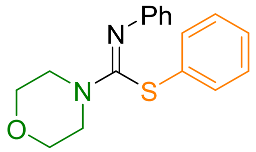

The reaction was performed using the general procedure above with phenyl acid chloride oxime (31.1 mg, 0.2 mmol), morpholine (17.4 mg, 0.2 mmol) and thiophenol (110.2 mg, 1.0 mmol). Following silica gel chromatography (12 g silica gel column, 0% → 100% gradient using DCM and ethyl acetate), product **4h** was isolated as a clear oil (50.1 mg, 84%). The isolated yield reported in the manuscript is the average of two runs (84% and 84% yield).

^{1}H NMR (400 MHz, CDCl$_3$): 7.17 – 7.23 (m, 7H), 6.98 (t, *J* = 7.5 Hz, 1H), 6.78 (d, *J* = 7.2 Hz, 2H), 3.60 (q, *J* = 5.0 Hz, 4H), 3.53 (q, *J* = 5.1 Hz, 4H) ppm.

^{13}C NMR (101 MHz, CDCl$_3$): 153.8, 150.1, 133.2, 131.2, 129.1, 128.6, 127.3, 122.8, 121.9, 66.5, 48.5 ppm.

HRMS (ESI) m/z: [M + H]+ Calcd for $C_{17}H_{18}N_2OS$: 298.1140; Found 298.1138

phenyl (Z)-N-(4-(trifluoromethyl)phenyl)morpholine-4-carbimidate (4i)

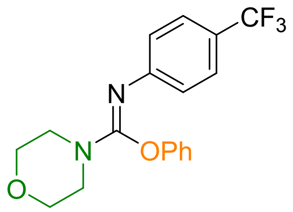

The reaction was performed using the general procedure above with (4-trifluoromethylphenyl) acid chloride oxime (44.7 mg, 0.2 mmol), morpholine (17.4 mg, 0.2 mmol) and phenol (94.1 mg, 1.0 mmol). Following silica gel chromatography (12 g silica gel column, 0% → 100% gradient using DCM and ethyl acetate), product **4i** was isolated as a clear oil (46.2 mg, 66%). The isolated yield reported in the manuscript is the average of two runs (66% and 63% yield).

^{1}H NMR (400 MHz, CDCl$_3$): 7.29 (d, J = 7.5 Hz, 2H), 7.17 (t, J = 8.1 Hz, 2H), 6.95 (t, J = 8.2 Hz, 1H), 6.89 (d, J = 8.0 Hz, 2H), 6.83 (d, J = 7.4 Hz, 2H), 3.73 (t, J = 5.1 Hz, 4H), 3.57 (t, J = 5.2 Hz, 4H) ppm.

^{13}C NMR (101 MHz, CDCl$_3$): 154.1, 150.3, 149.6, 129.7, 126.1 (q), 125.6, 124.2, 123.5, 123.0, 116.6, 66.6, 45.8 ppm.

^{19}F NMR (376 MHz, CDCl$_3$): -61.7 (s) ppm.

HRMS (ESI) m/z: [M + H]+ Calcd for $C_{18}H_{18}F_3N_2O_2$: 351.1320; Found 351.1313

phenyl (Z)-N-(6-methoxypyridin-3-yl)morpholine-4-carbimidate (4j)

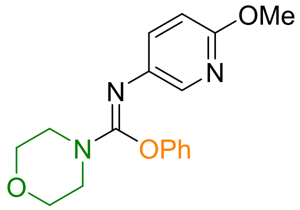

The reaction was performed using the general procedure above with (6-methoxy-3-pyridyl) acid chloride oxime (37.3 mg, 0.2 mmol), morpholine (17.4 mg, 0.2 mmol) and phenol (94.1 mg, 1.0 mmol). Following silica gel chromatography (12 g silica gel column, 0% → 100% gradient using DCM and ethyl acetate), product **4j** was isolated as a clear oil (47.0 mg, 75%). The isolated yield reported in the manuscript is the average of two runs (75% and 69% yield). Product **4j** was isolated as an 80:20 (Z:E) mixture of stereoisomers. The NMR information given below is for the major Z isomer.

^{1}H NMR (400 MHz, CDCl$_3$): 7.74 (d, J = 2.7 Hz, 1H), 7.12 – 7.20 (m, 3H), 6.95 (t, J = 7.5 Hz, 1H), 6.84 (d, J = 7.7 Hz, 2H), 6.47 (d, J = 8.7 Hz, 1H), 3.80 (s, 3H), 3.71 (t, J = 5.1 Hz, 4H), 3.53 (t, J = 5.0 Hz, 4H) ppm.

^{13}C NMR (101 MHz, CDCl$_3$): 177.5, 159.8, 154.1, 149.7, 140.4, 137.0, 133.9, 129.8, 123.3, 116.1, 110.0, 66.6, 53.4, 45.7 ppm.

HRMS (ESI) m/z: [M + H]+ Calcd for $C_{17}H_{20}N_3O_3$: 314.1504; Found 314.1510

phenyl (Z)-N-((E)-styryl)morpholine-4-carbimidate (4k)

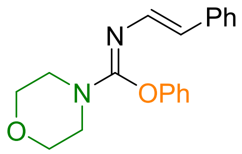

The reaction was performed using the general procedure above with (transcinnamaldehyde) acid chloride oxime (36.3 mg, 0.2 mmol), morpholine (17.4 mg, 0.2 mmol) and phenol (94.1 mg, 1.0 mmol). Following silica gel chromatography (12 g silica gel column, 0% → 100% gradient using DCM and ethyl acetate), product **4k** was isolated as a clear oil (51.2 mg, 83%). The isolated yield reported in the manuscript is the average of two runs (83% and 83% yield).

^{1}H NMR (400 MHz, CDCl$_3$): 7.32 – 7.37 (m, 3H), 7.19 (d, J = 6.2 Hz, 4H), 7.06 – 7.11 (m, 2H), 6.99 (d, J = 6.1 Hz, 2H), 6.37 (d, J = 10.8 Hz, 1H), 3.67 (t, J = 5.2 Hz, 4H), 3.53 (t, J = 5.3 Hz, 4H) ppm.

^{13}C NMR (101 MHz, CDCl$_3$): 155.0, 150.3, 138.0, 132.7, 130.3, 128.5, 126.1, 125.8, 123.4, 121.6, 115.5, 66.6, 45.6 ppm.

HRMS (ESI) m/z: [M + H]+ Calcd for $C_{19}H_{21}N_2O_2$: 309.1603; Found 309.1602

phenyl (Z)-N-phenyl-1,4-dioxa-8-azaspiro[4.5]decane-8-carbimidate (4l)

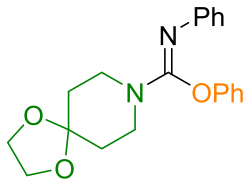

The reaction was performed using the general procedure above with phenyl acid chloride oxime (31.1 mg, 0.2 mmol), 1,4-dioxa-8-azaspiro[4.5]decane (28.6mg, 0.2 mmol) and phenol (94.1 mg, 1.0 mmol). Following silica gel chromatography (12 g silica gel column, 0% → 100% gradient using DCM and ethyl acetate), product **4l** was isolated as a clear oil (64.3 mg, 95%). The isolated yield reported in the manuscript is the average of two runs (95% and 93% yield).

^{1}H NMR (400 MHz, CDCl$_3$): 7.15 (t, J = 7.5 Hz, 2H), 7.06 (t, J = 7.4 Hz, 2H), 6.92 (t, J = 7.5 Hz, 1H), 6.79 – 6.86 (m, 5H), 3.98 (s, 4H), 3.64 (t, J = 5.2 Hz, 4H), 1.74 (t, J = 5.1 Hz, 4H) ppm.

^{13}C NMR (101 MHz, CDCl$_3$): 154.6, 148.9, 147.1, 129.5, 128.4, 123.0, 122.9, 122.1, 116.6, 107.2, 64.5, 43.7, 34.9 ppm.

HRMS (ESI) m/z: [M + H]+ Calcd for $C_{20}H_{23}N_2O_3$: 339.1708; Found 339.1710

benzyl (Z)-4-(phenoxy(phenylimino)methyl)-1,4-diazepane-1-carboxylate (4m)

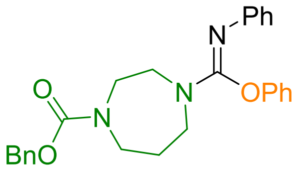

The reaction was performed using the general procedure above with phenyl acid chloride oxime (31.1 mg, 0.2 mmol), benzyl 1,4-diazepane-1-carboxylate (46.9 mg, 0.2 mmol) and phenol (94.1 mg, 1.0 mmol). Following silica gel chromatography (12 g silica gel column, 0% → 100% gradient using DCM and ethyl acetate), product **4m** was isolated as a clear oil (78.1 mg, 91%). The isolated yield reported in the manuscript is the average of two runs (91% and 90% yield).

^{1}H NMR (400 MHz, $CDCl_3$): 7.31 – 7.28 (m, 5H), 7.11 – 7.16 (m, 2H), 7.02 – 7.07 (m, 2H), 6.91 (t, *J* = 6.8 Hz, 1H), 6.77 – 6.84 (m, 5H), 5.18 (d, *J* = 11.8 Hz, 2H), 3.68 (s, 4H), 3.50 – 3.61 (m, 4H), 1.94 (sept, *J* = 6.2 Hz, 2H) ppm.

^{13}C NMR (101 MHz, $CDCl_3$): 156.0, 154.2, 148.4, 146.9, 136.8, 129.5, 128.6, 128.3, 128.1, 128.0, 127.9, 123.1, 122.9, 122.0, 116.3, 67.3, 49.7, 49.4, 48.4, 47.5, 46.6, 46.1, 27.2 ppm.

HRMS (ESI) m/z: [M + H]+ Calcd for $C_{26}H_{28}N_3O_3$: 430.2130; Found 430.2127

11. NMR spectra of isolated products

(Z)-N-phenylmorpholine-4-carbimidoyl fluoride (2a)

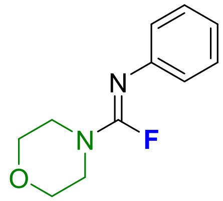

^{1}H NMR: 400 MHz in $CDCl_3$

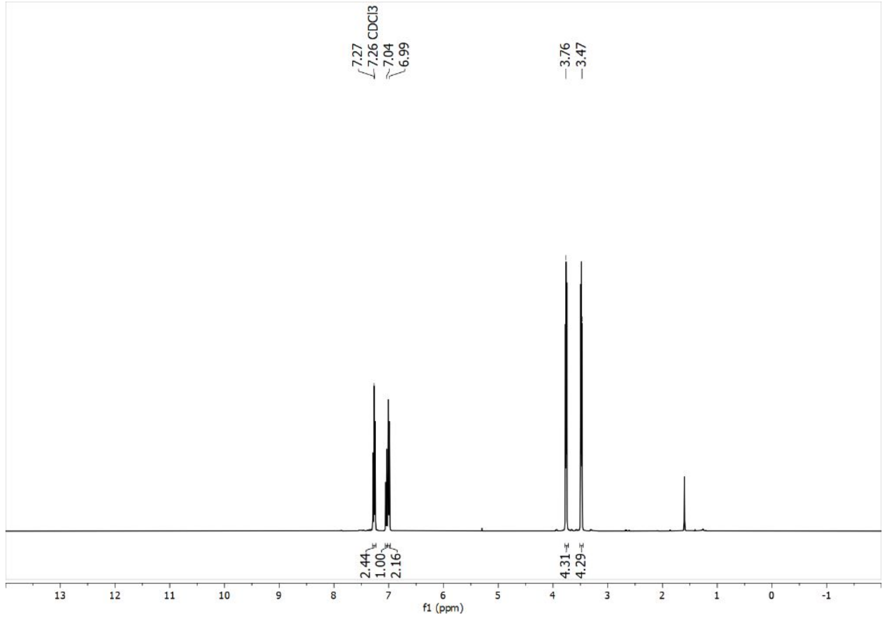

^{13}C NMR: 101 MHz in $CDCl_3$

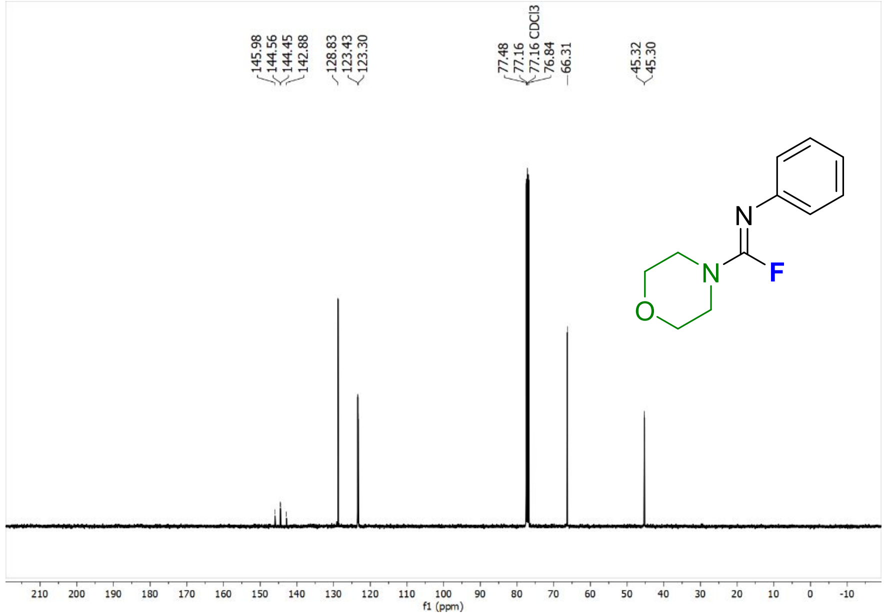

^{19}F NMR: 376 MHz in $CDCl_3$

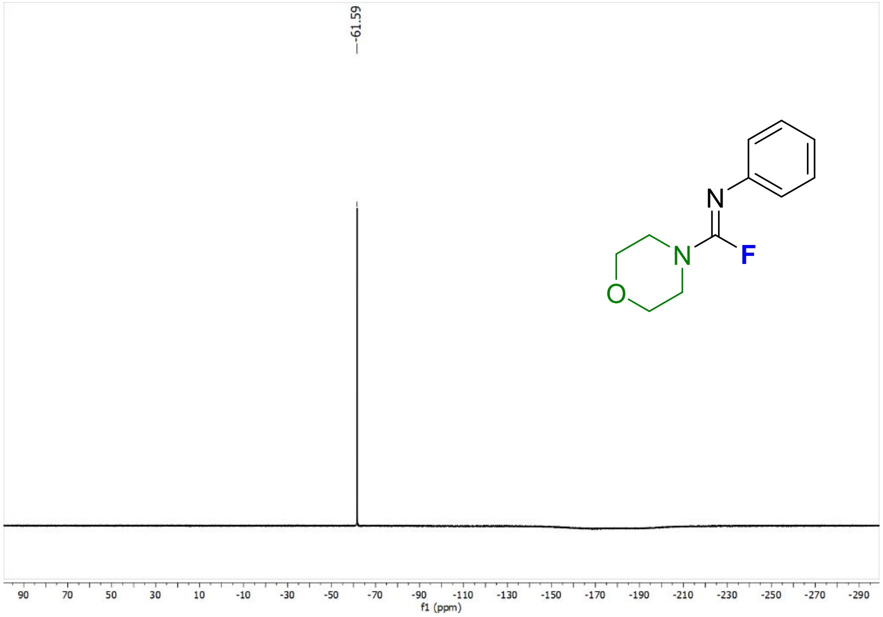

(Z)-N-(4-chlorophenyl)morpholine-4-carbimidoyl fluoride (2b)

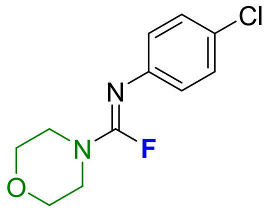

^{1}H NMR: 400 MHz in $CDCl_3$

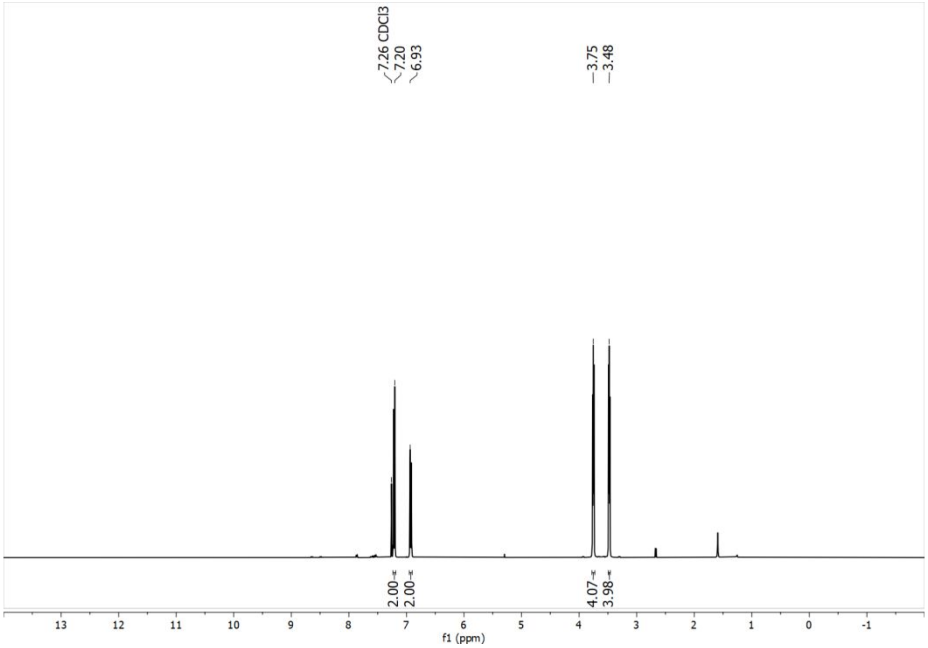

^{13}C NMR: 101 MHz in $CDCl_3$

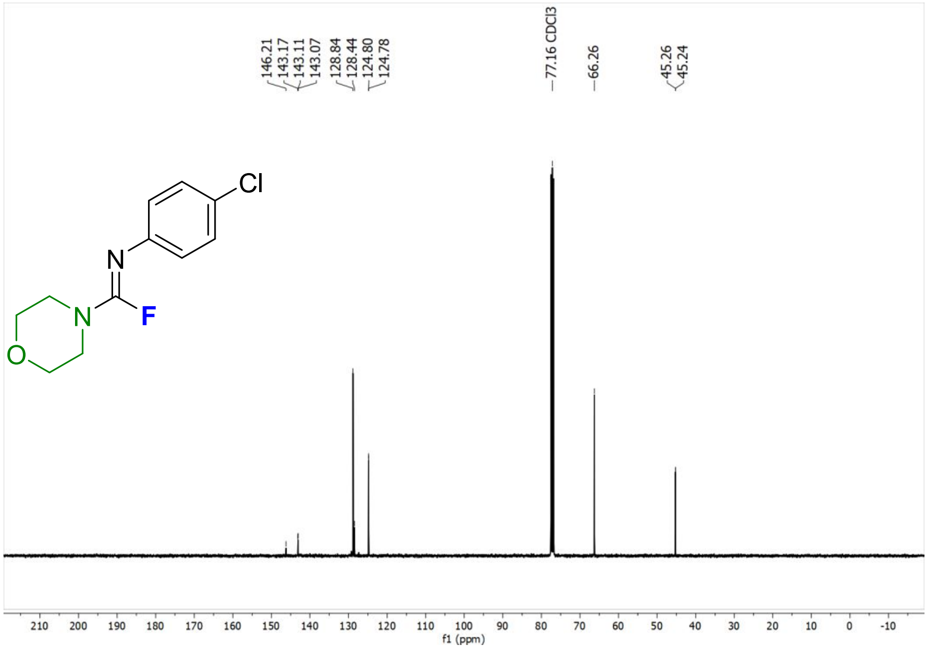

^{19}F NMR: 376 MHz in $CDCl_3$

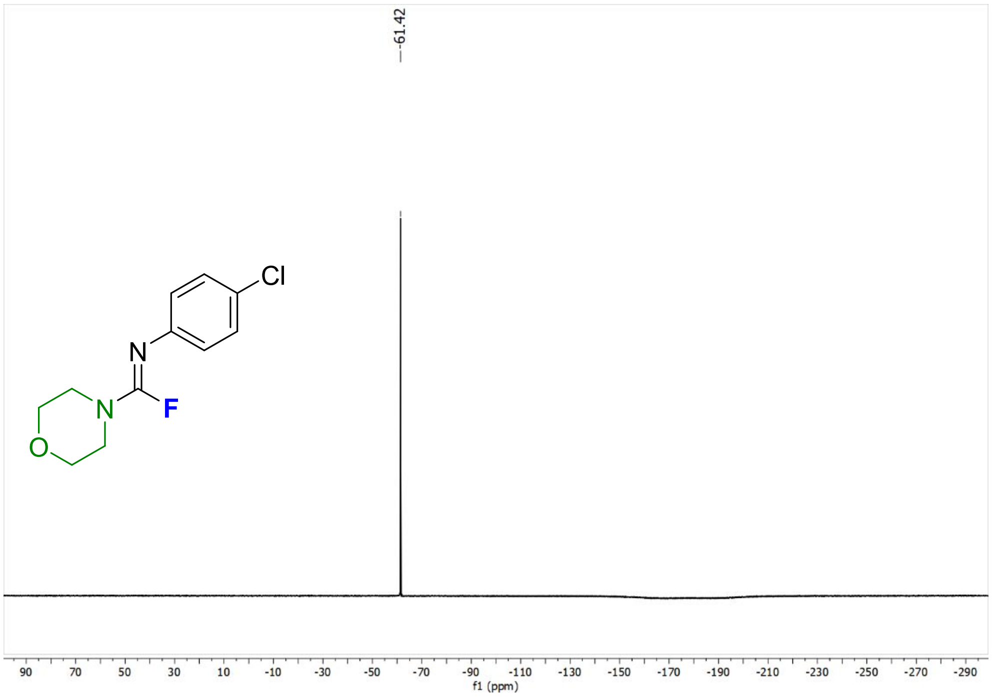

(Z)-N-(4-cyanophenyl)morpholine-4-carbimidoyl fluoride (2c)

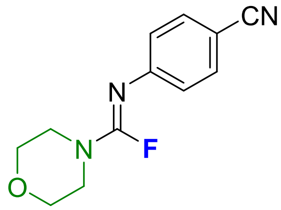

^{1}H NMR: 400 MHz in $CDCl_3$

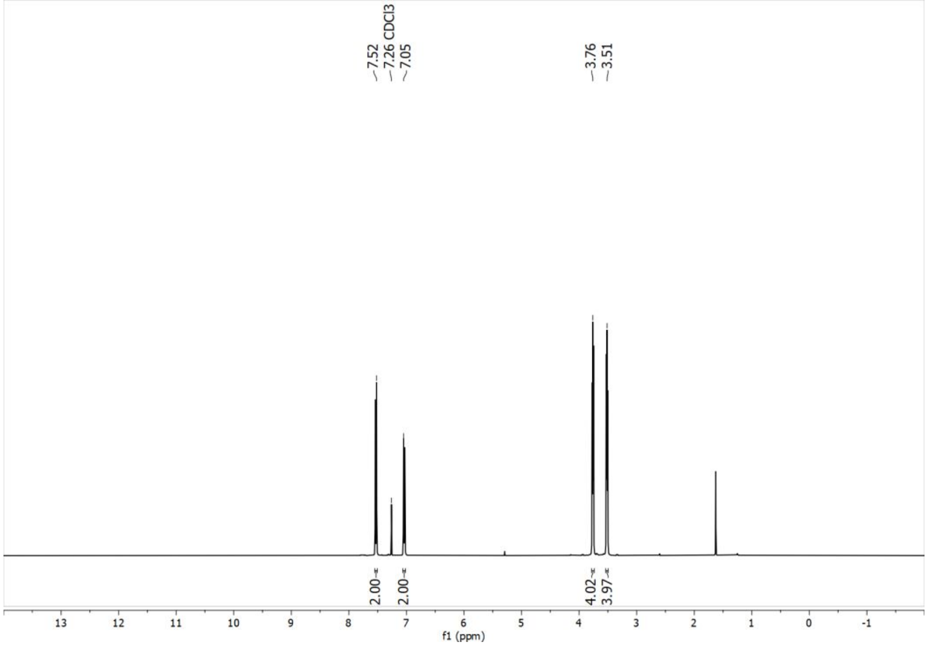

^{13}C NMR: 101 MHz in $CDCl_3$

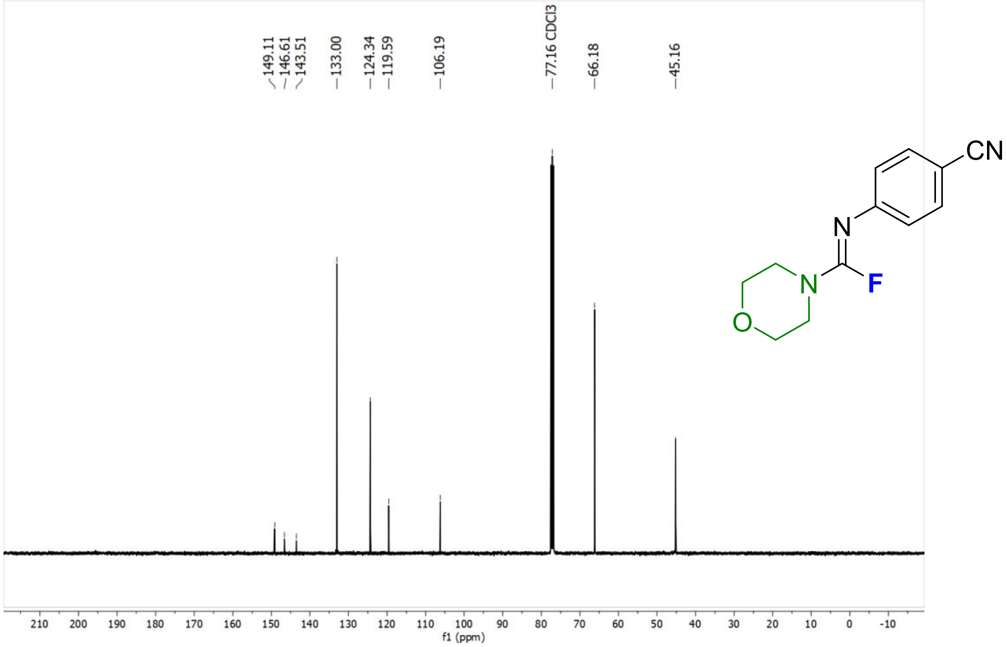

^{19}F NMR: 376 MHz in $CDCl_3$

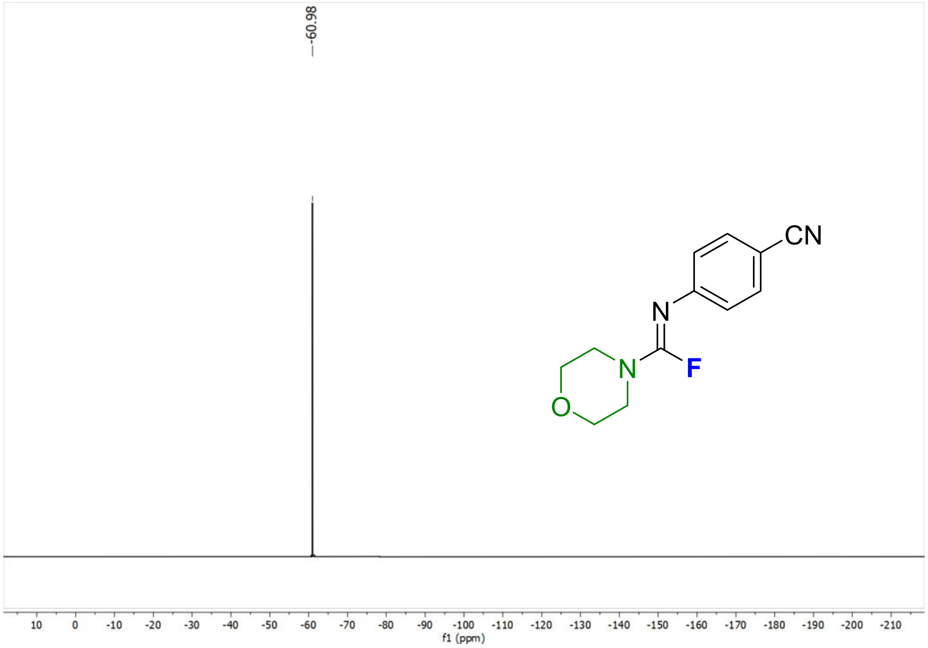

methyl (Z)-4-((fluoro(morpholino)methylene)amino)benzoate (2d)

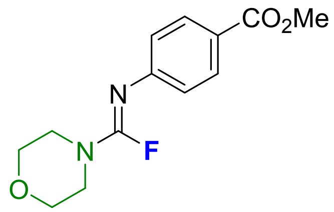

^{1}H NMR: 400 MHz in $CDCl_3$

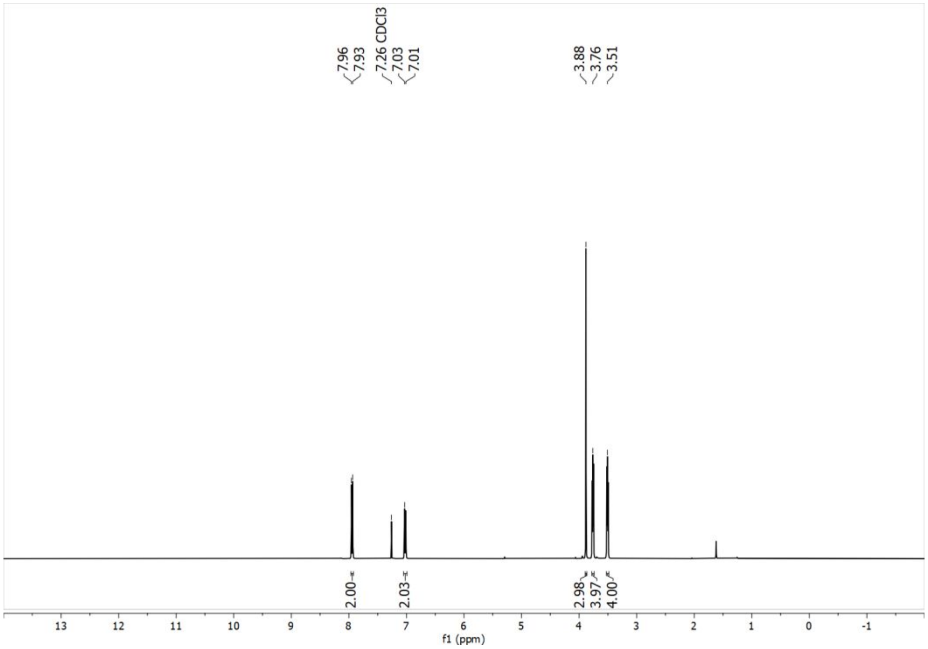

^{13}C NMR: 101 MHz in $CDCl_3$

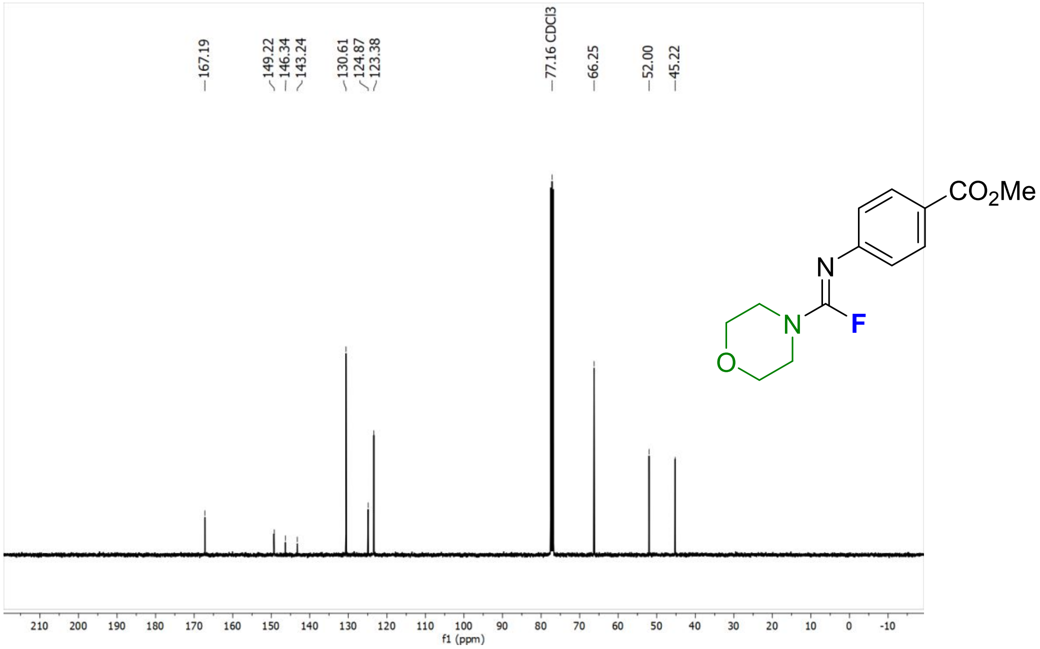

^{19}F NMR: 376 MHz in $CDCl_3$

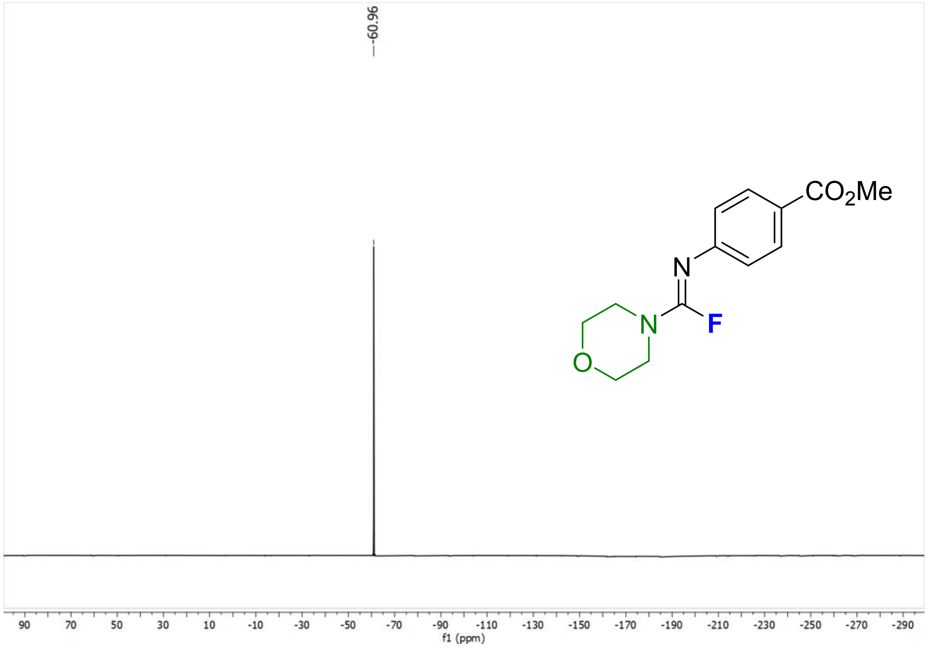

(Z)-N-(4-(trifluoromethyl)phenyl)morpholine-4-carbimidoyl fluoride (2e)

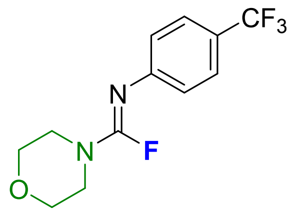

^{1}H NMR: 400 MHz in $CDCl_3$

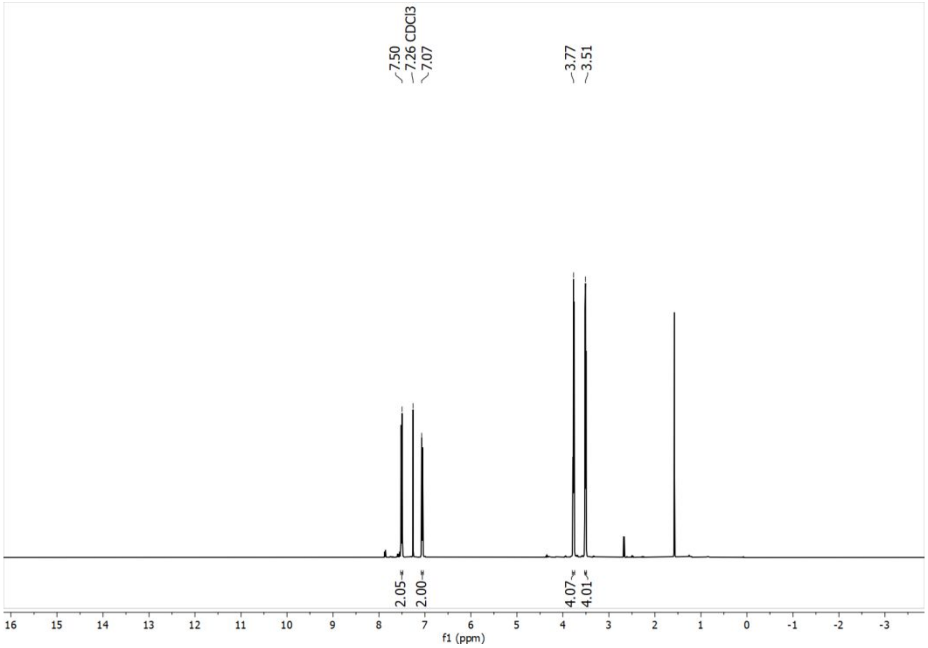

^{13}C NMR: 101 MHz in $CDCl_3$

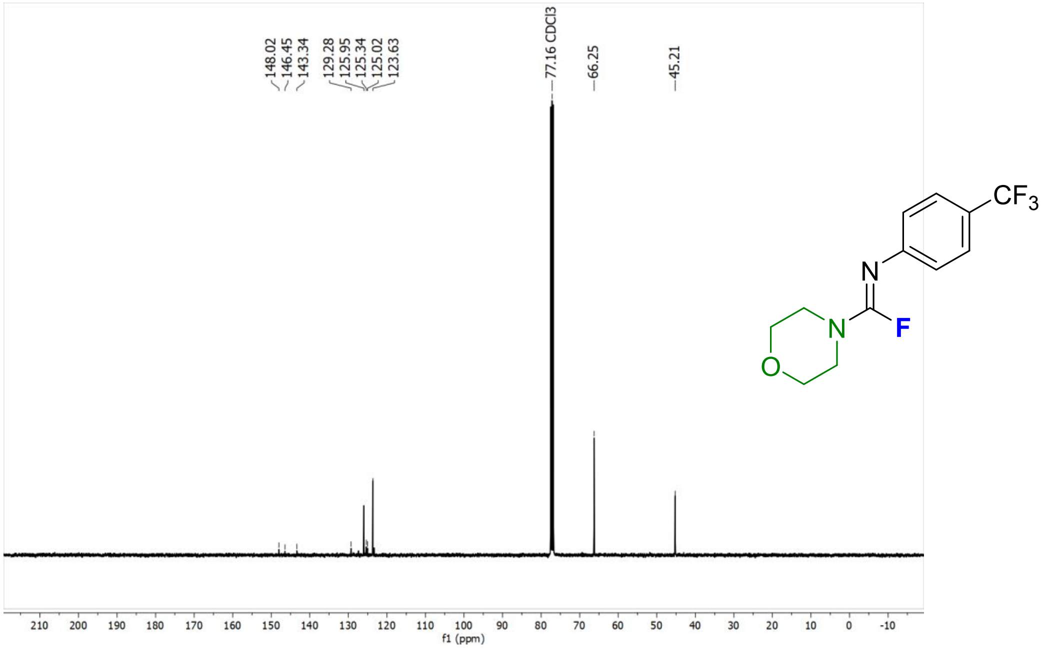

^{19}F NMR: 376 MHz in $CDCl_3$

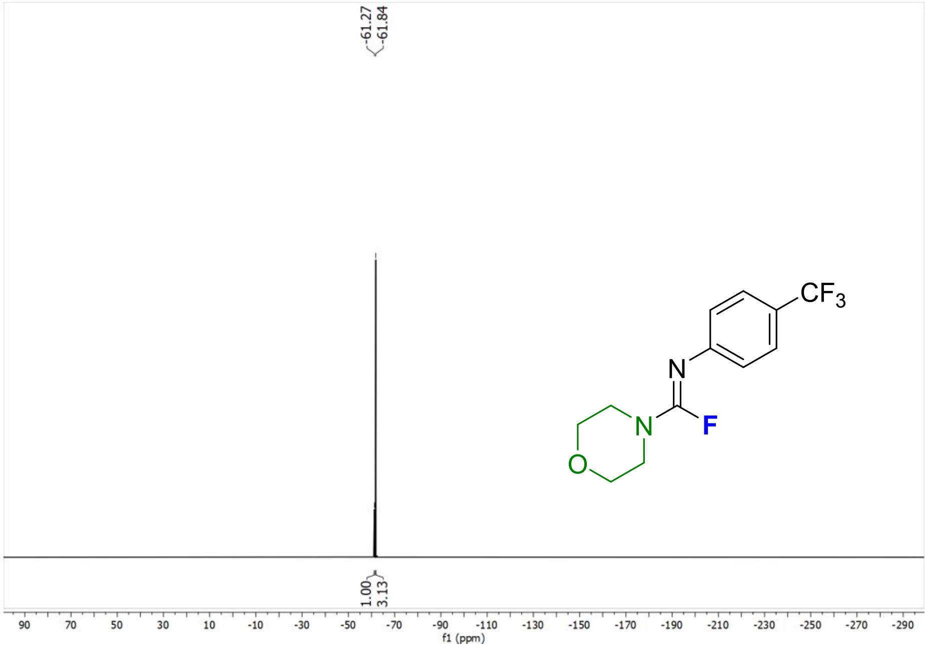

(Z)-N-(4-nitrophenyl)morpholine-4-carbimidoyl fluoride (2f)

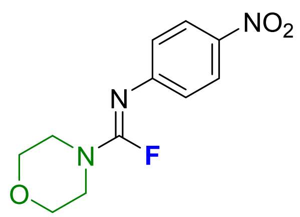

^{1}H NMR: 400 MHz in $CDCl_3$

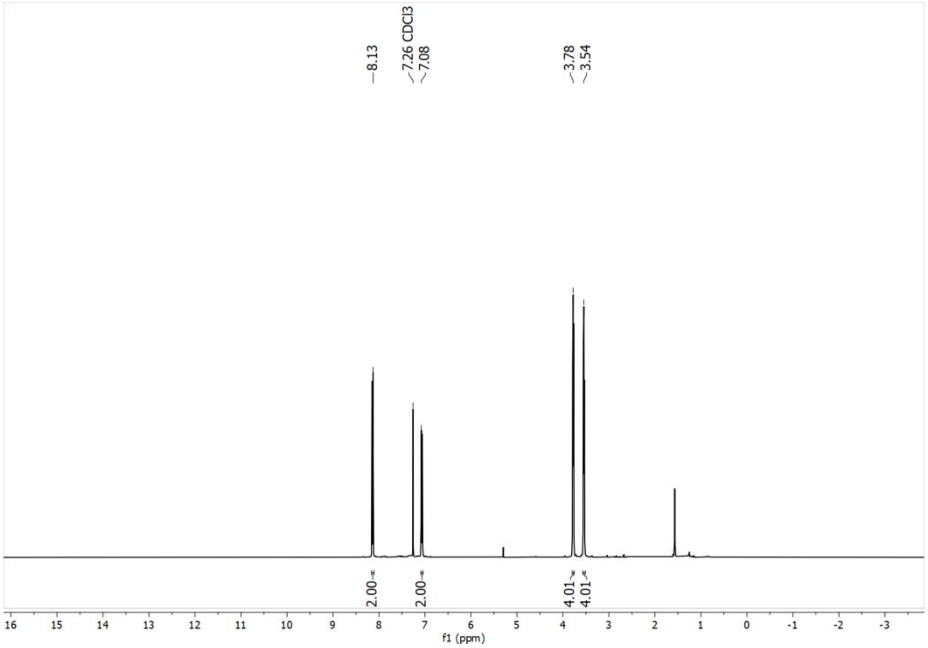

^{13}C NMR: 101 MHz in $CDCl_3$

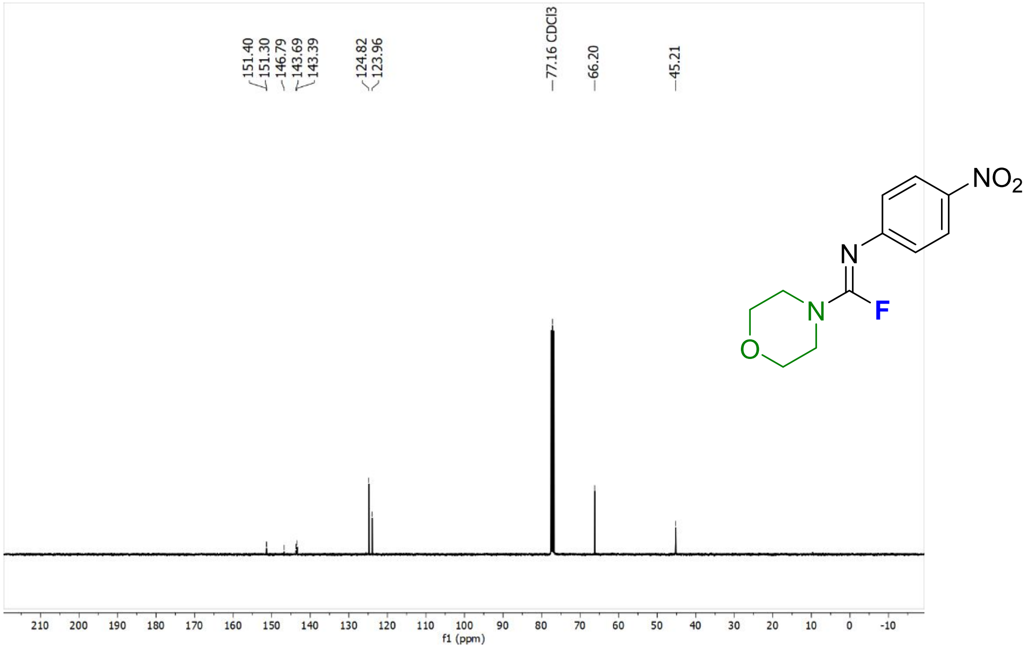

^{19}F NMR: 376 MHz in $CDCl_3$

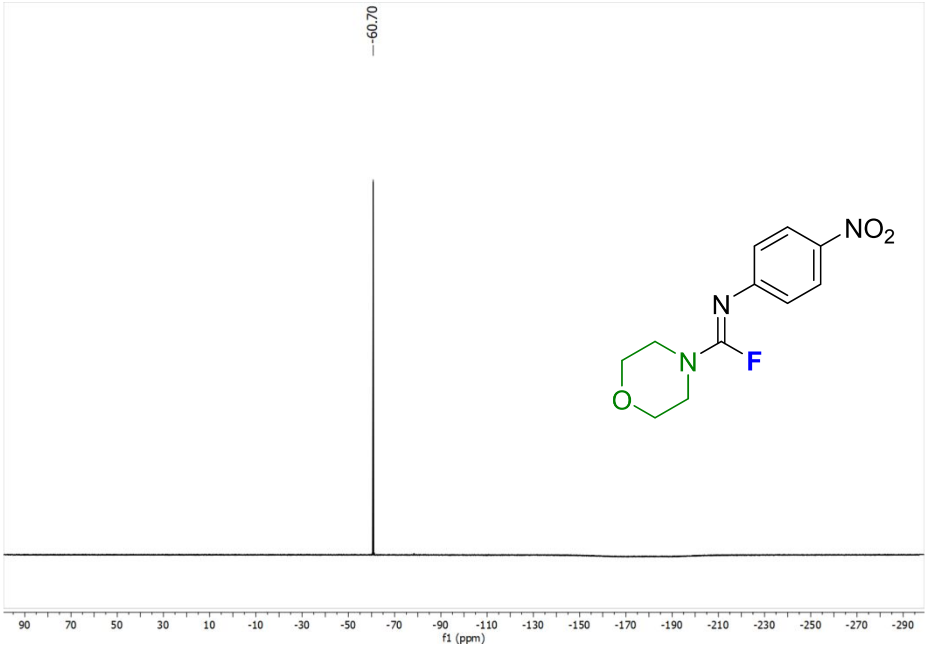

(Z)-N-(4-methoxyphenyl)morpholine-4-carbimidoyl fluoride (2g)

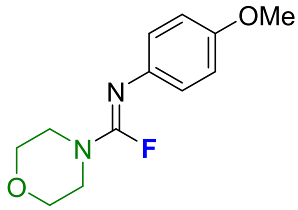

^{1}H NMR: 400 MHz in $CDCl_3$

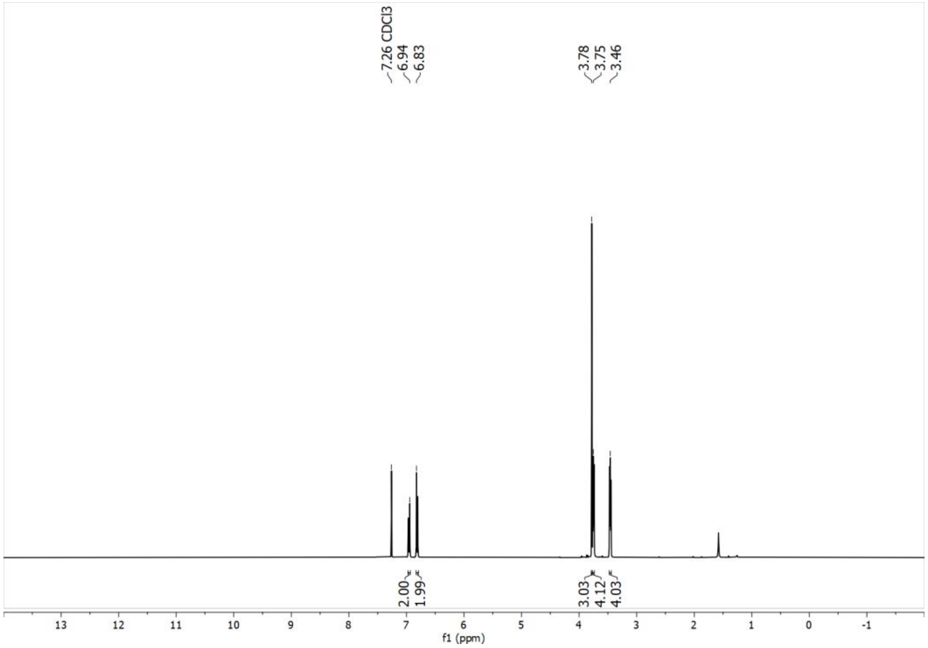

^{13}C NMR: 101 MHz in $CDCl_3$

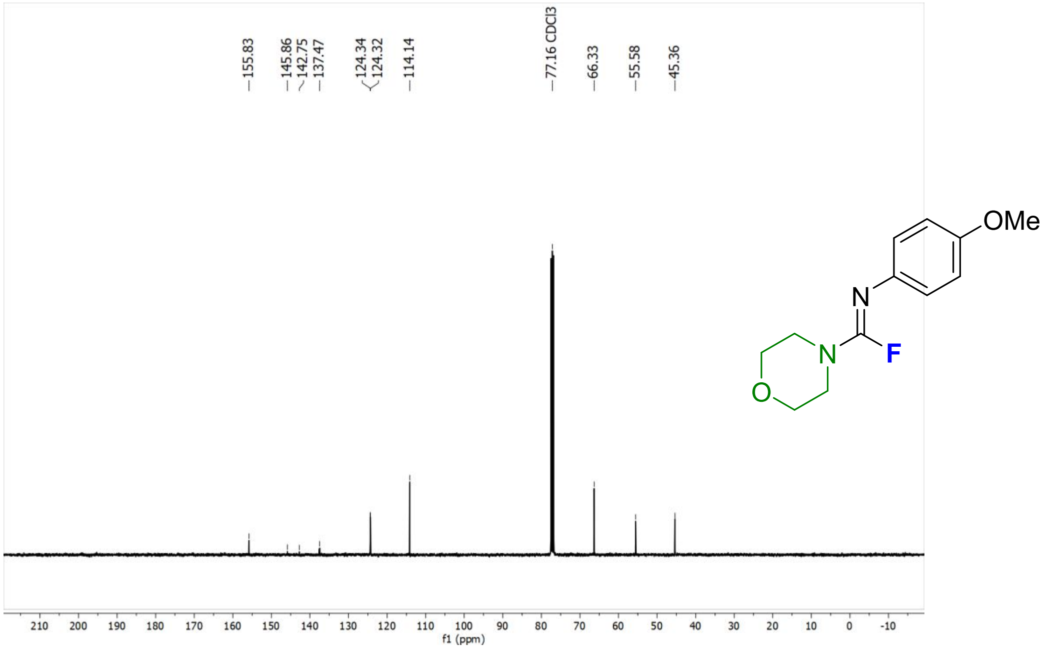

^{19}F NMR: 376 MHz in $CDCl_3$

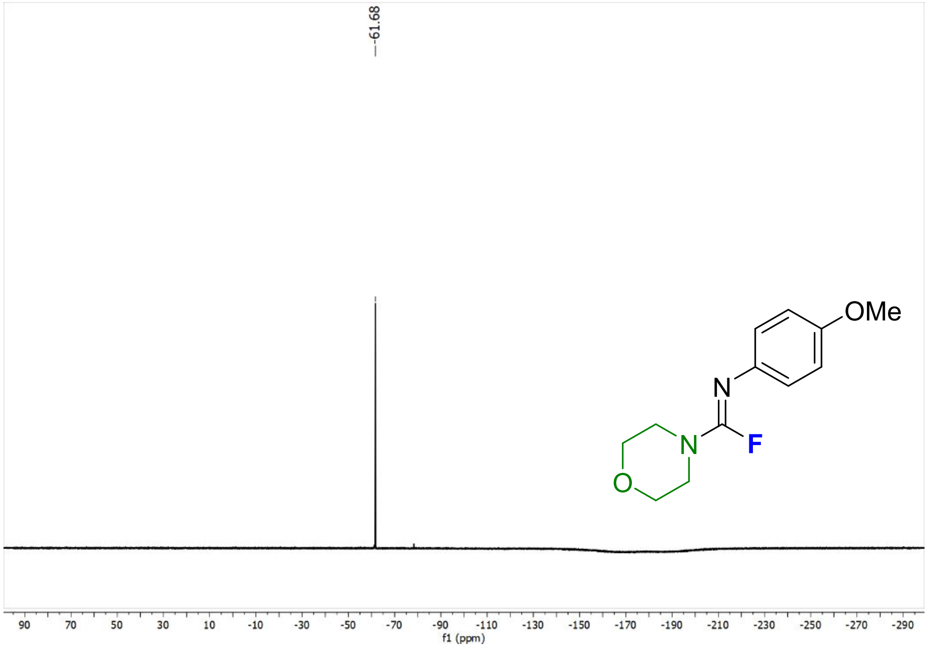

(Z)-N-(*m*-tolyl)morpholine-4-carbimidoyl fluoride (2h)

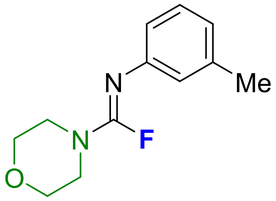

^{1}H NMR: 400 MHz in $CDCl_3$

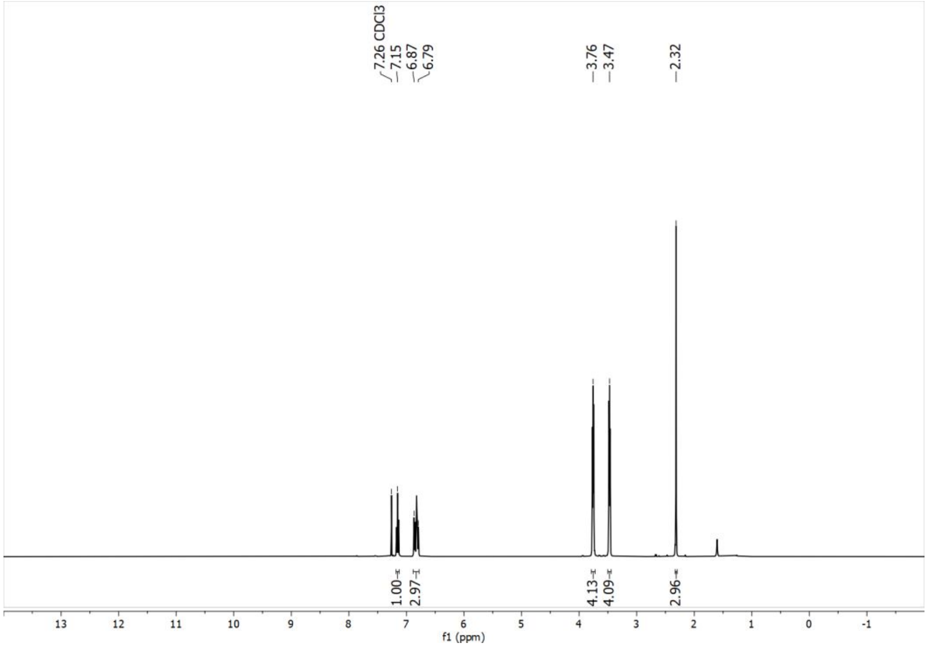

^{13}C NMR: 101 MHz in $CDCl_3$

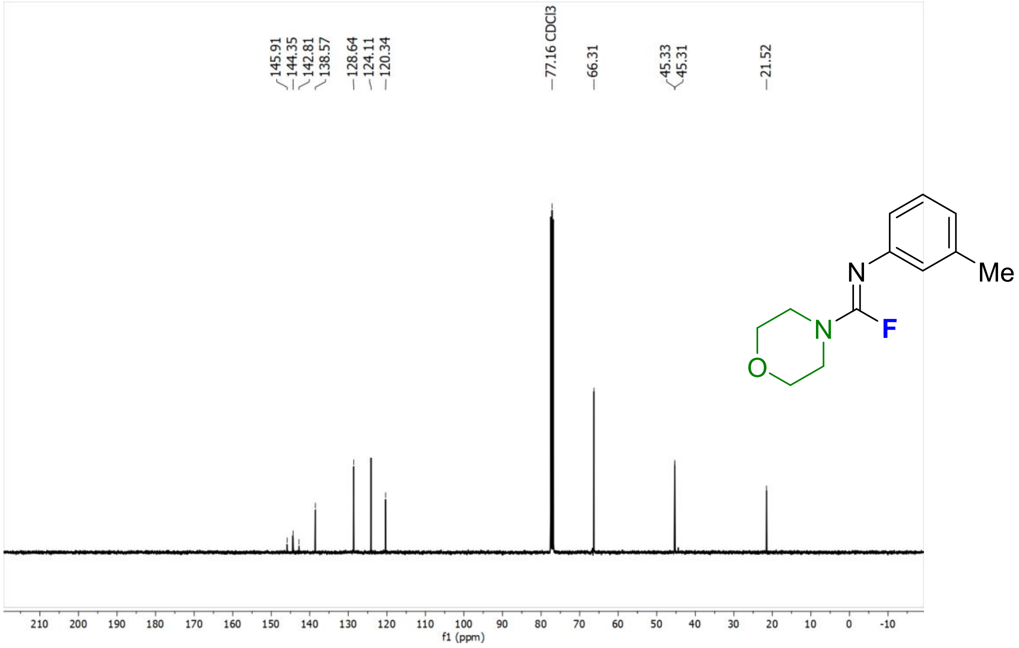

^{19}F NMR: 376 MHz in $CDCl_3$

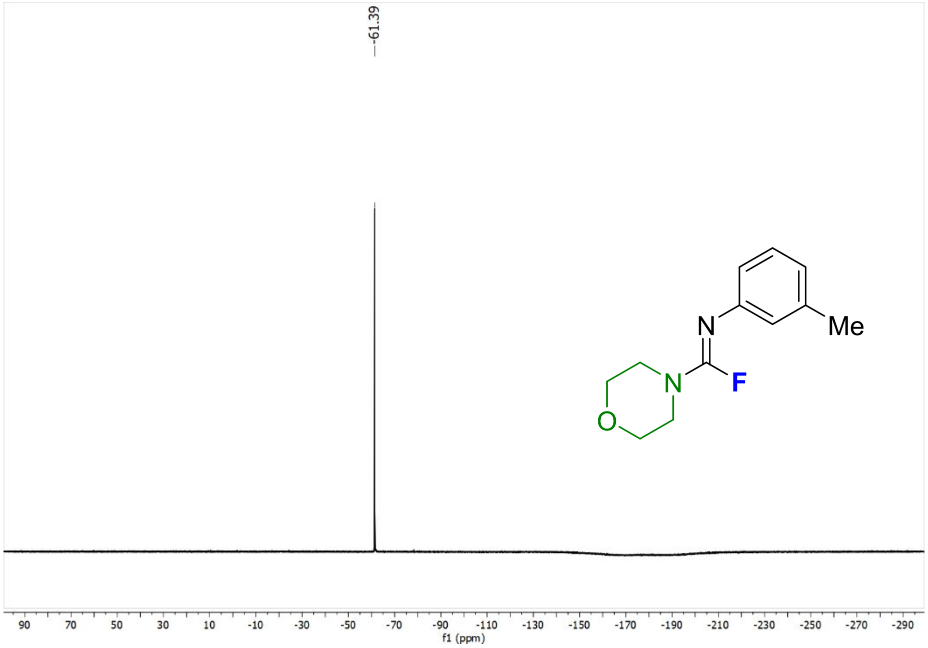

(Z)-N-(*o*-tolyl)morpholine-4-carbimidoyl fluoride (2i)

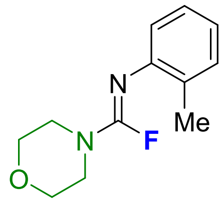

^{1}H NMR: 400 MHz in $CDCl_3$

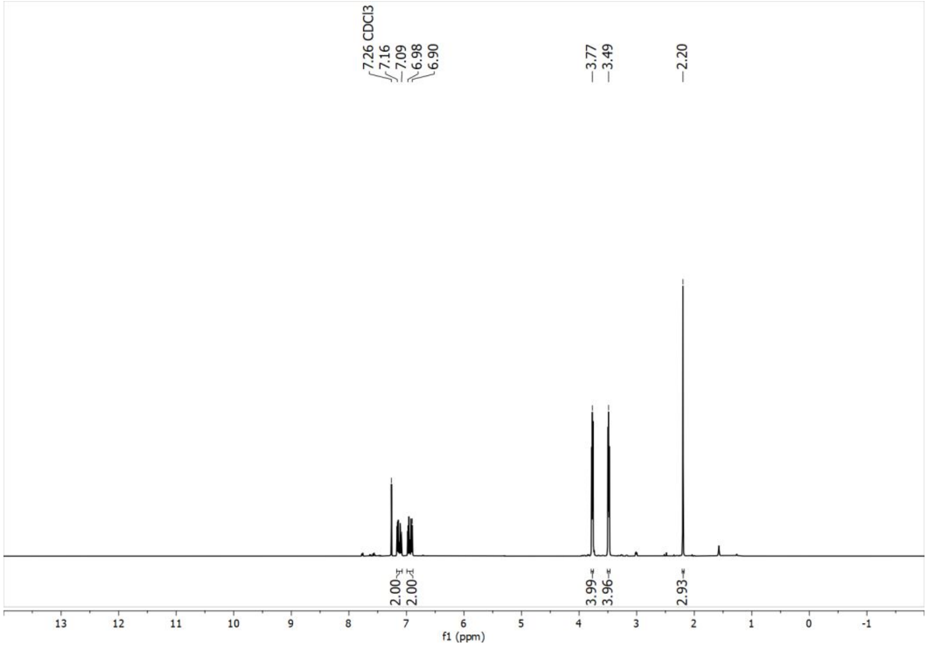

^{13}C NMR: 101 MHz in $CDCl_3$

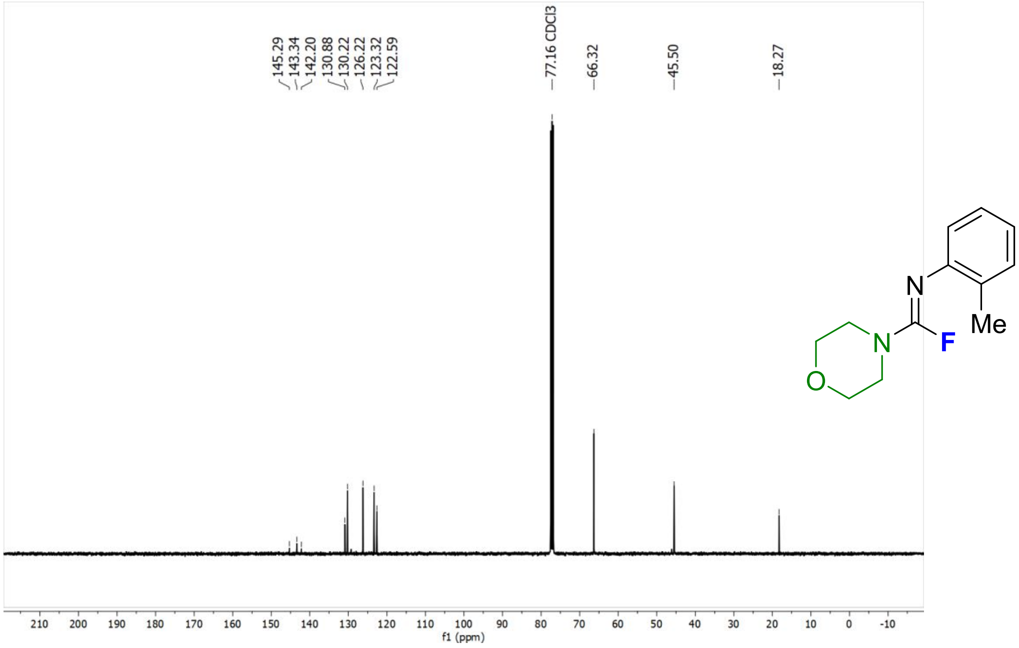

^{19}F NMR: 376 MHz in $CDCl_3$

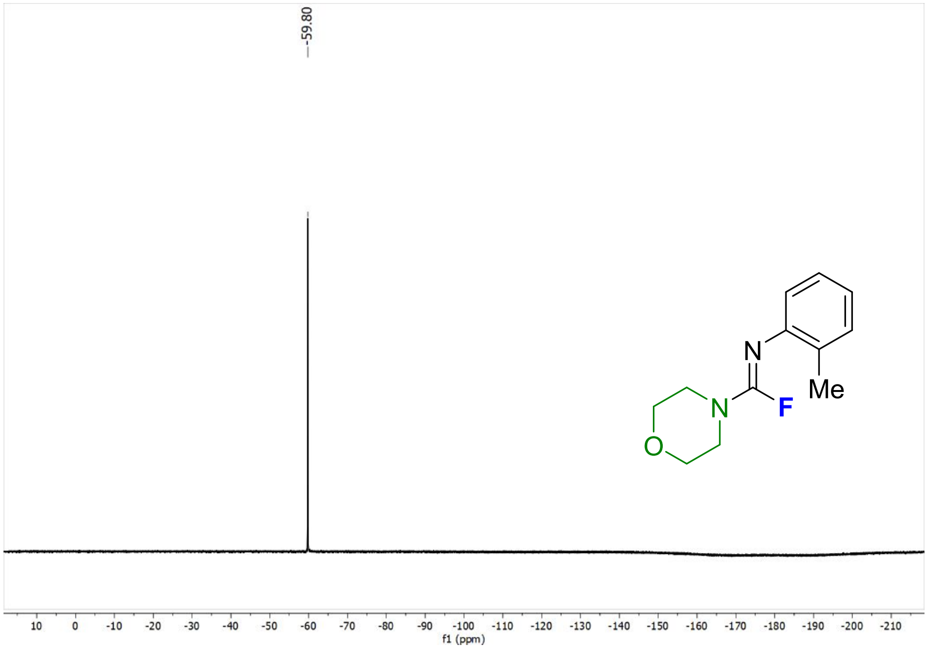

(Z)-N-(2-(trifluoromethoxy)phenyl)morpholine-4-carbimidoyl fluoride (2j)

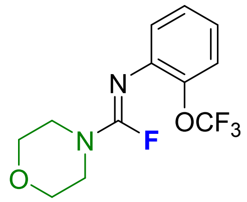

^{1}H NMR: 400 MHz in $CDCl_3$

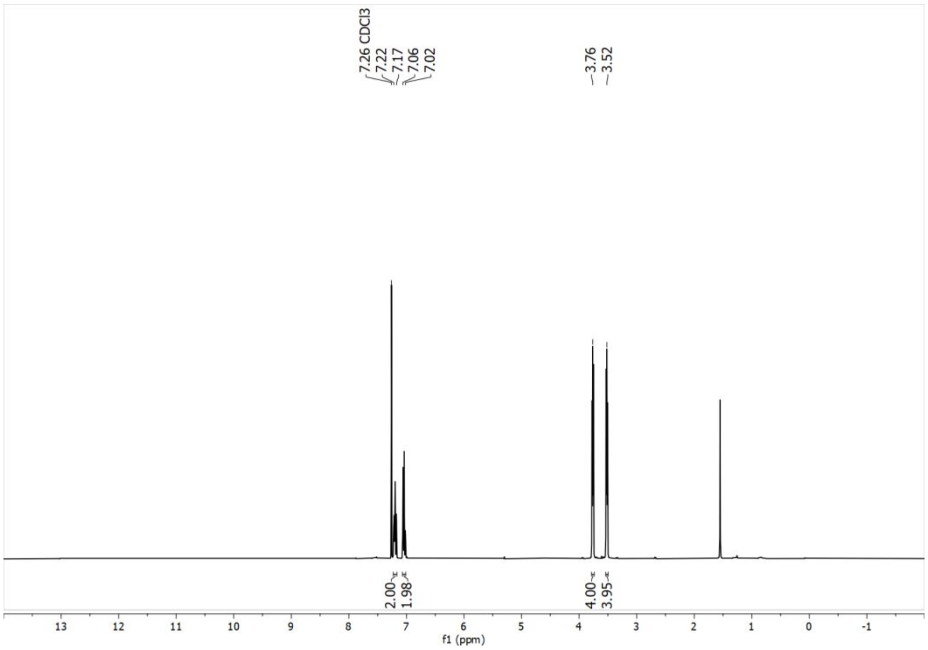

^{13}C NMR: 101 MHz in $CDCl_3$

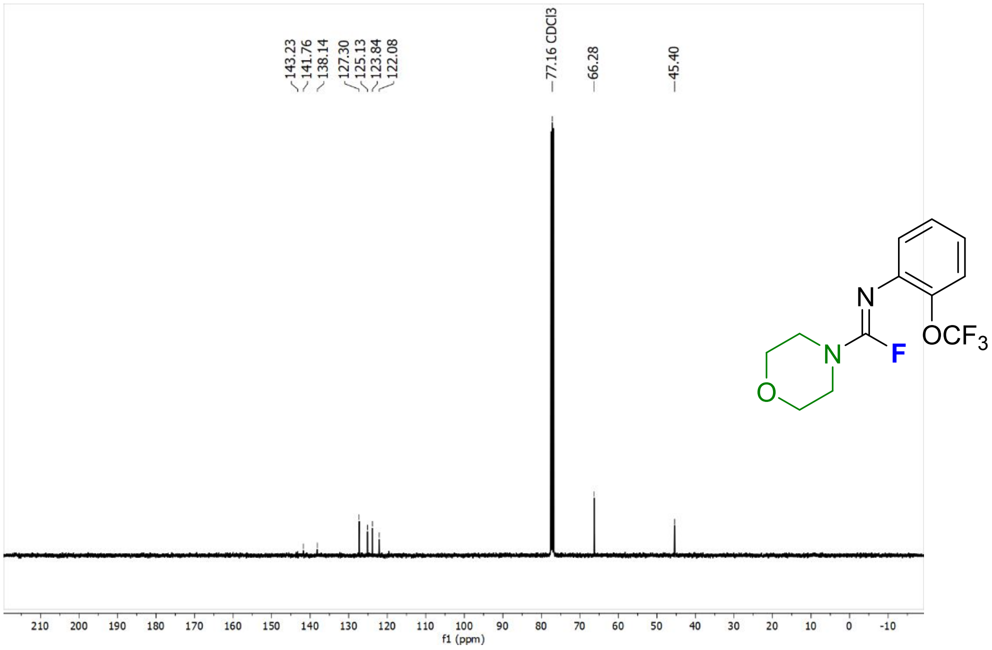

^{19}F NMR: 376 MHz in $CDCl_3$

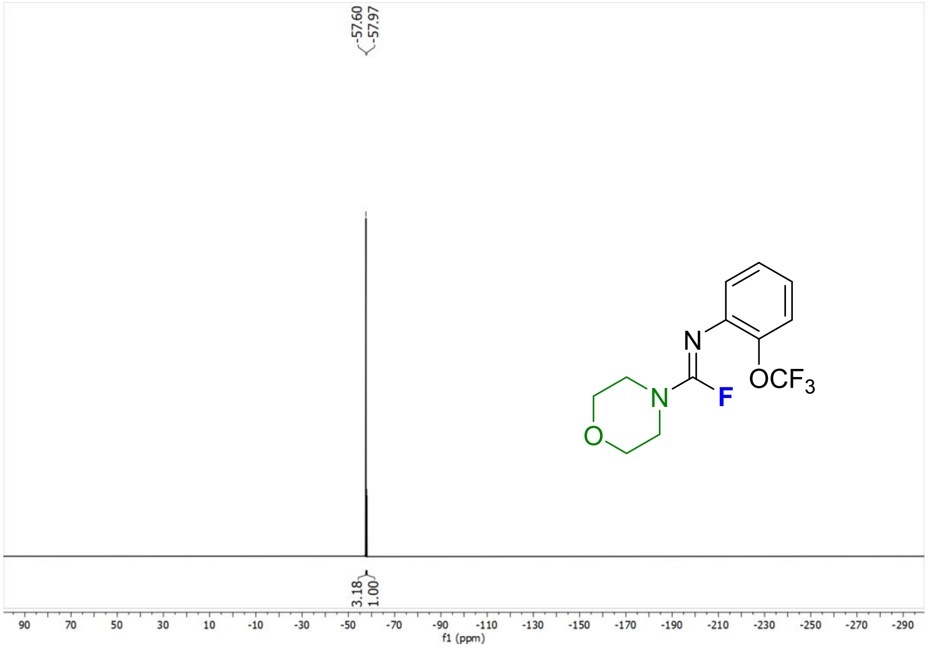

(Z)-N-(naphthalen-1-yl)morpholine-4-carbimidoyl fluoride (2k)

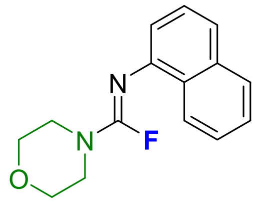

^{1}H NMR: 400 MHz in $CDCl_3$

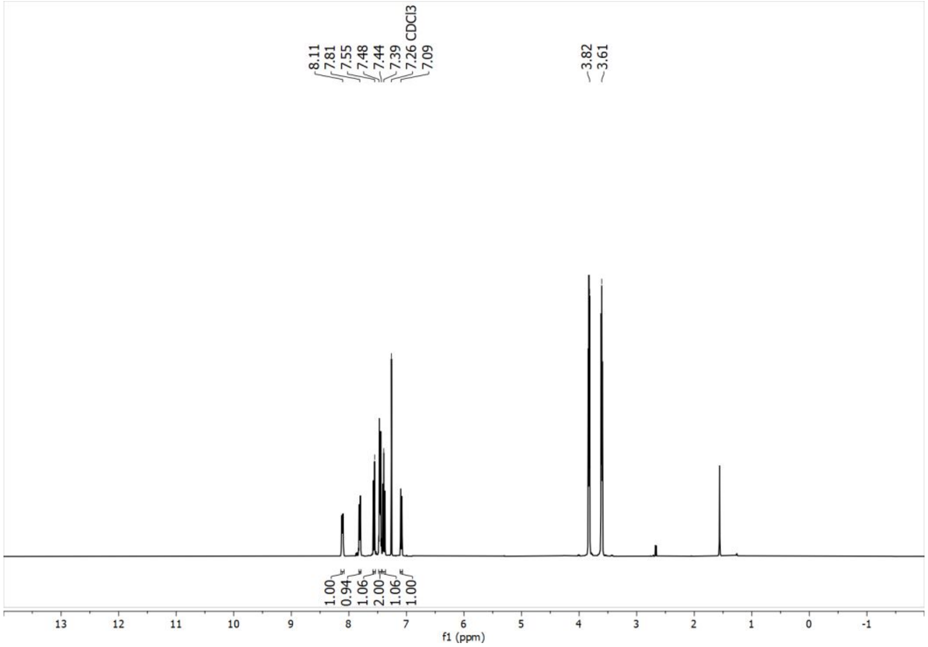

^{13}C NMR: 101 MHz in $CDCl_3$

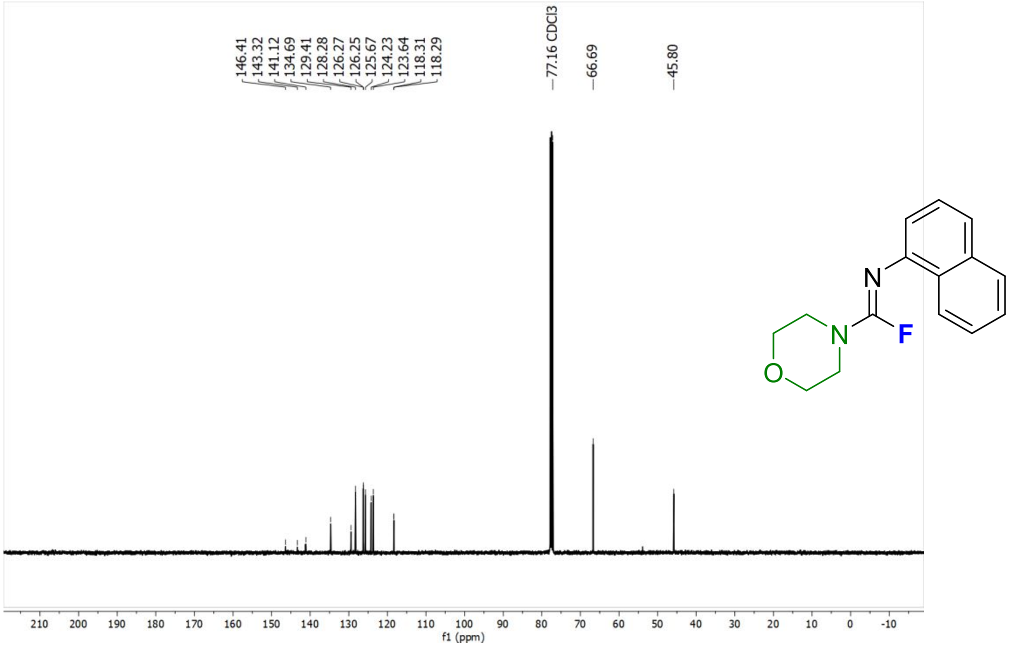

^{19}F NMR: 376 MHz in $CDCl_3$

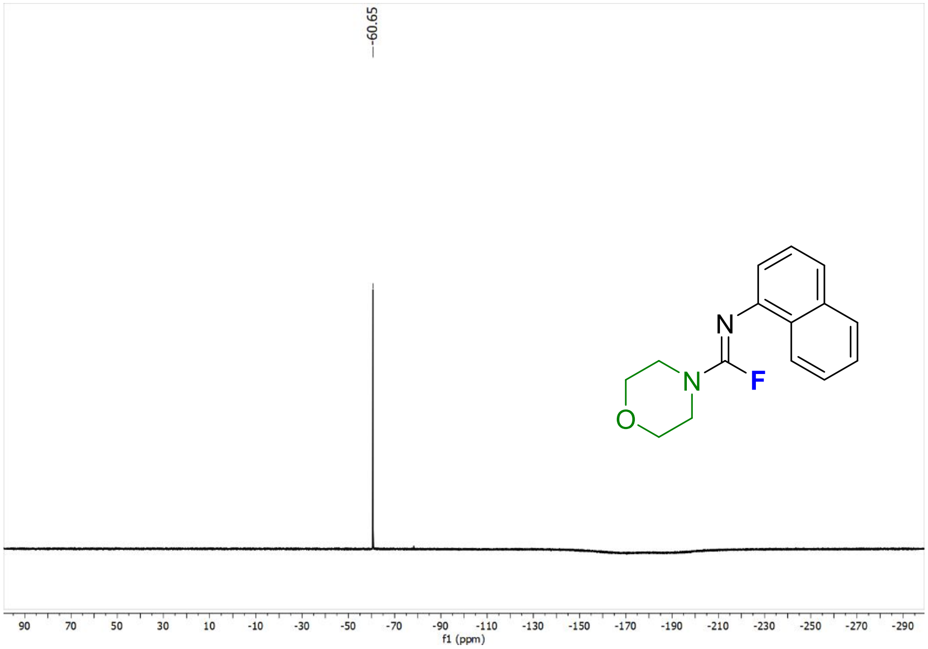

(Z)-N-(2,6-dimethylphenyl)morpholine-4-carbimidoyl fluoride (2l)

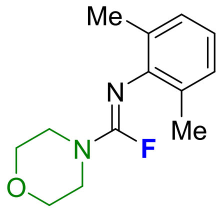

^{19}F NMR: 376 MHz in acetonitrile – crude reaction mixture with 1.0 eq. of 4-fluoroanisole (-125.35 ppm) as internal standard. Product peak is present at -55.7 ppm (9%).

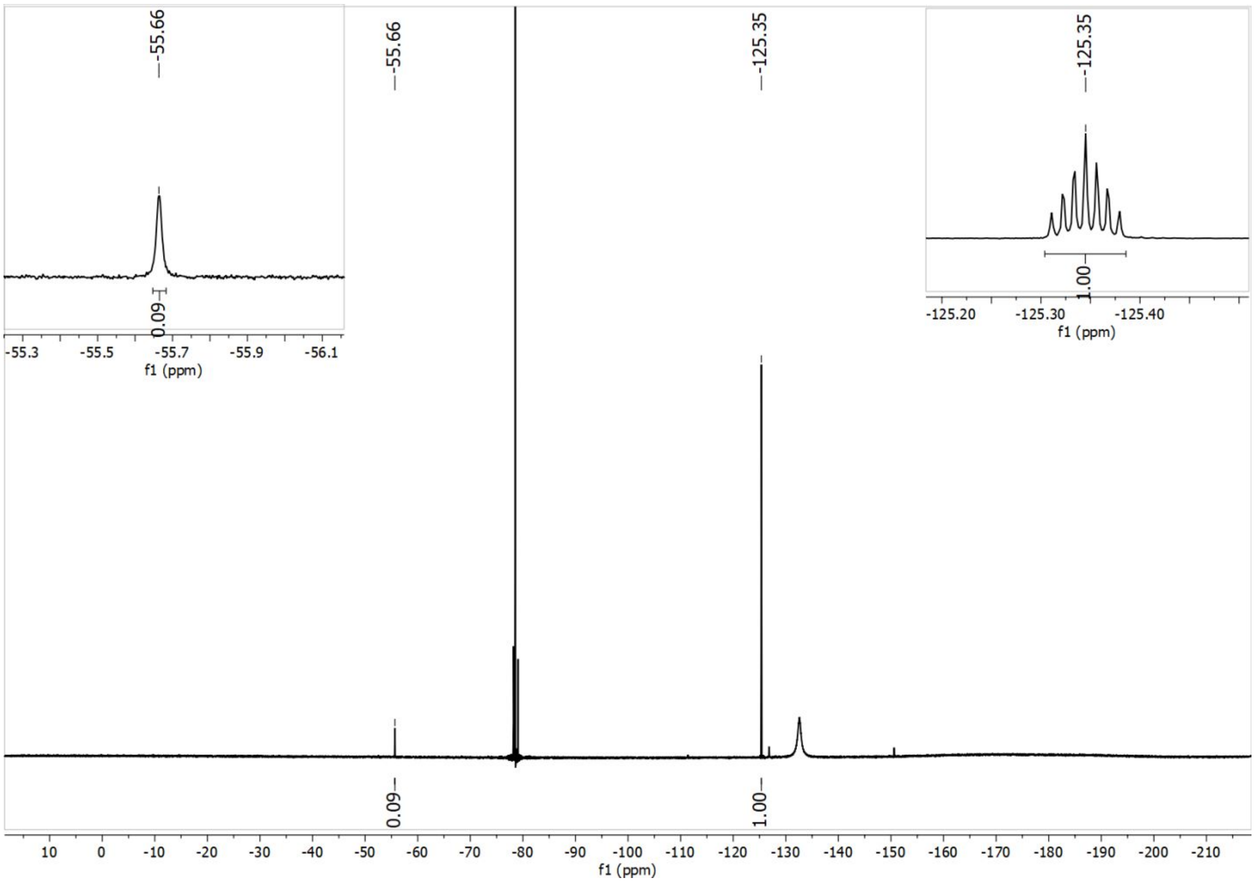

(Z)-N-(6-methoxypyridin-3-yl)morpholine-4-carbimidoyl fluoride (2m)

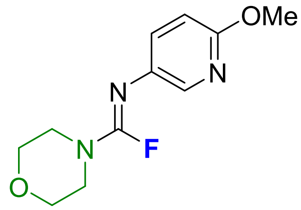

^{1}H NMR: 400 MHz in $CDCl_3$

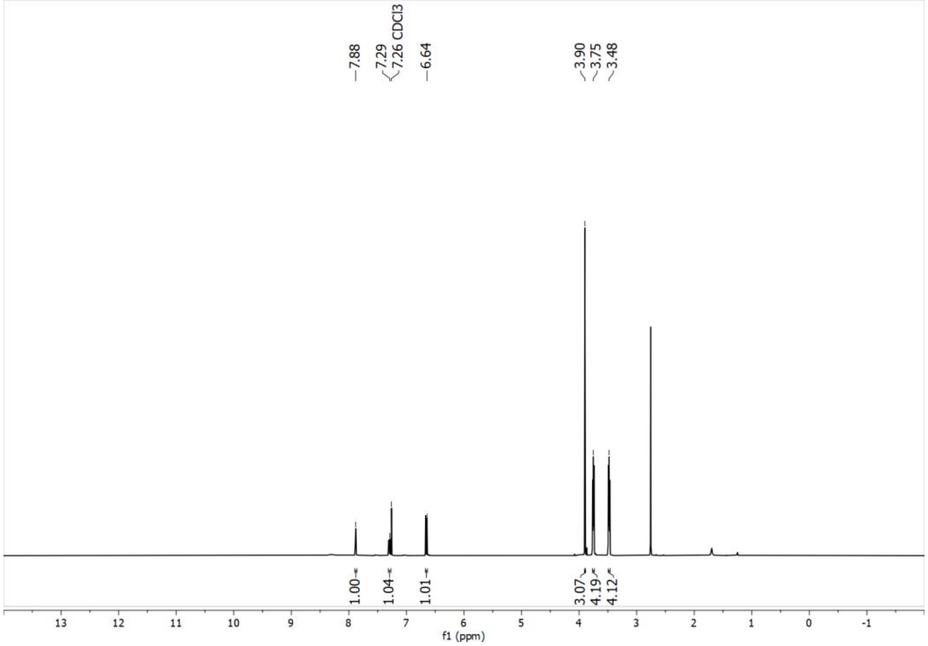

^{13}C NMR: 101 MHz in $CDCl_3$

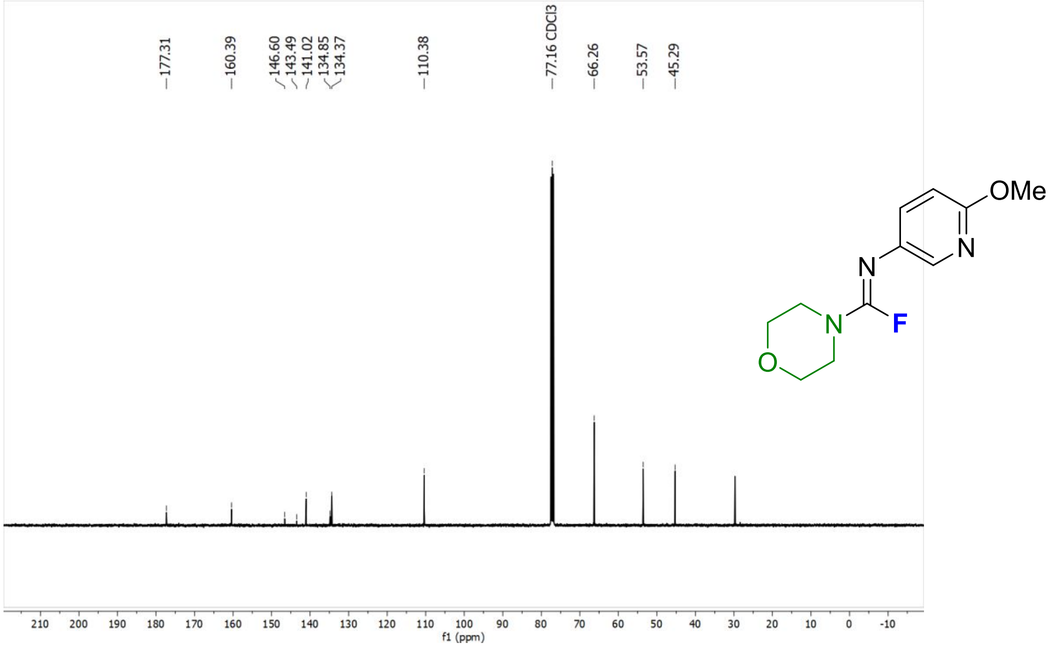

^{19}F NMR: 376 MHz in $CDCl_3$

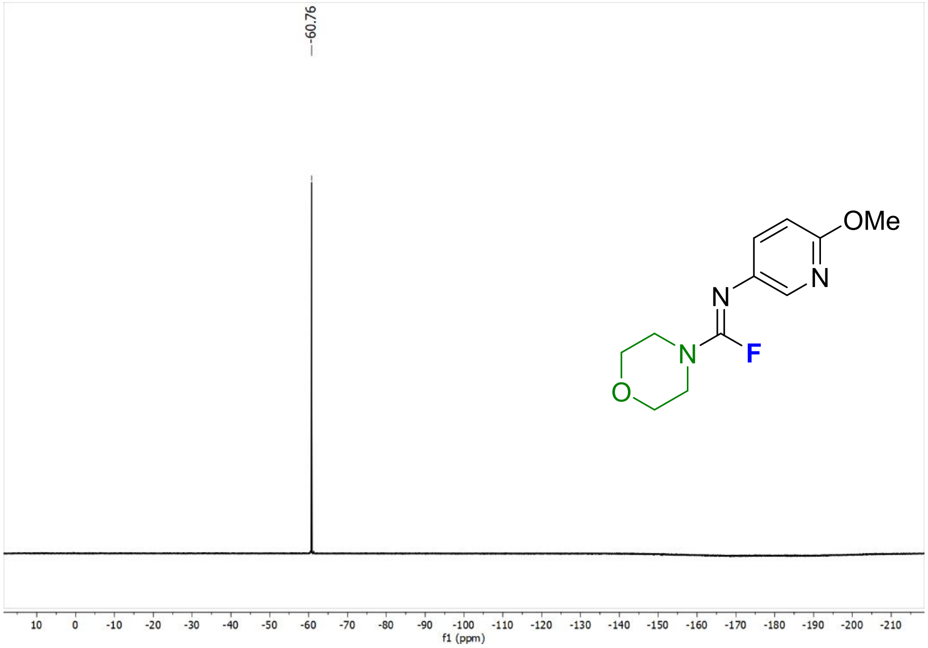

(Z)-N-(thiophen-2-yl)morpholine-4-carbimidoyl fluoride (2n)

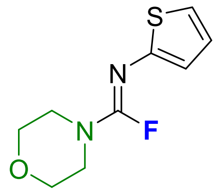

^{1}H NMR: 400 MHz in $CDCl_3$

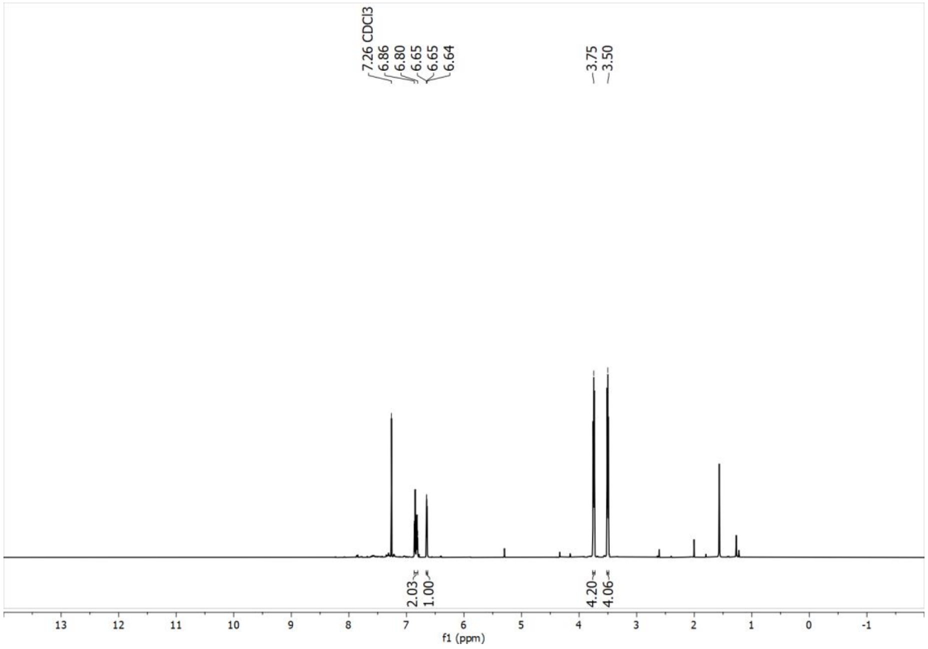

^{13}C NMR: 101 MHz in $CDCl_3$

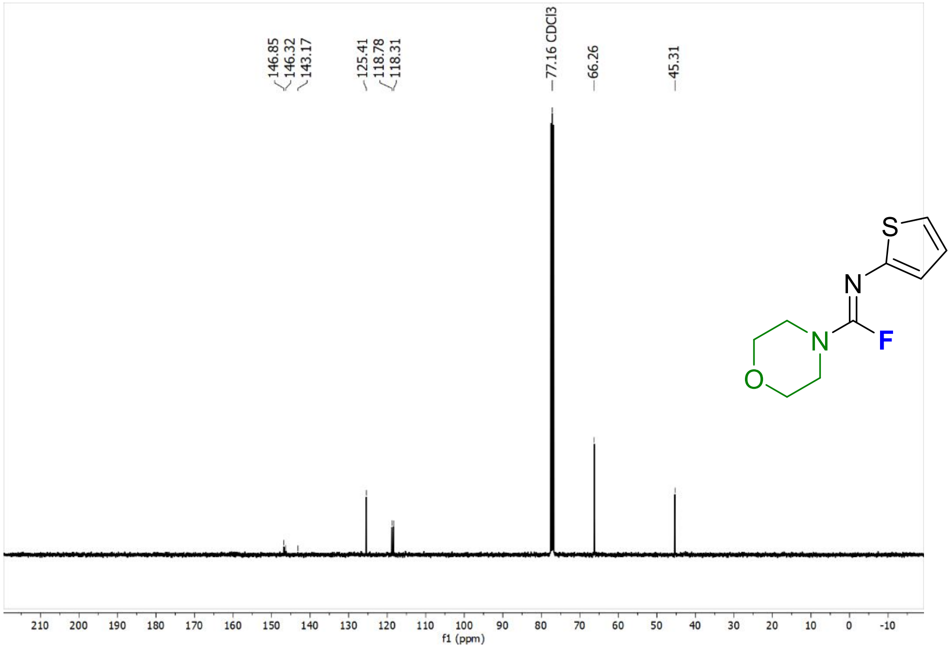

^{19}F NMR: 376 MHz in $CDCl_3$

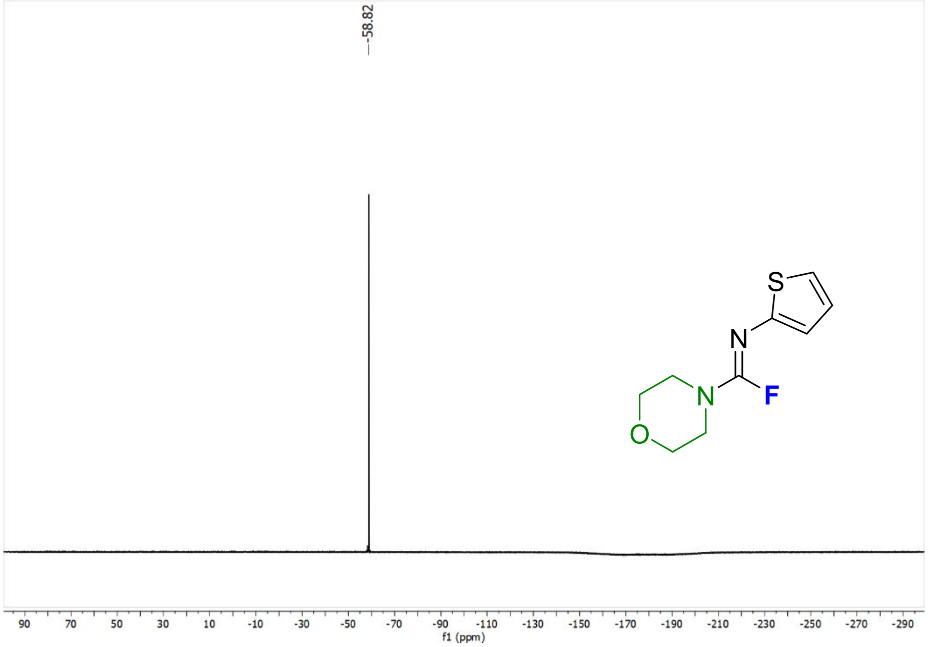

(Z)-N-(benzo[b]thiophen-3-yl)morpholine-4-carbimidoyl fluoride (2o)

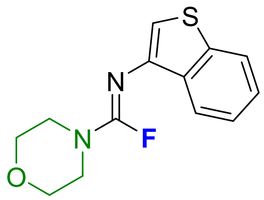

^{1}H NMR: 400 MHz in $CDCl_3$

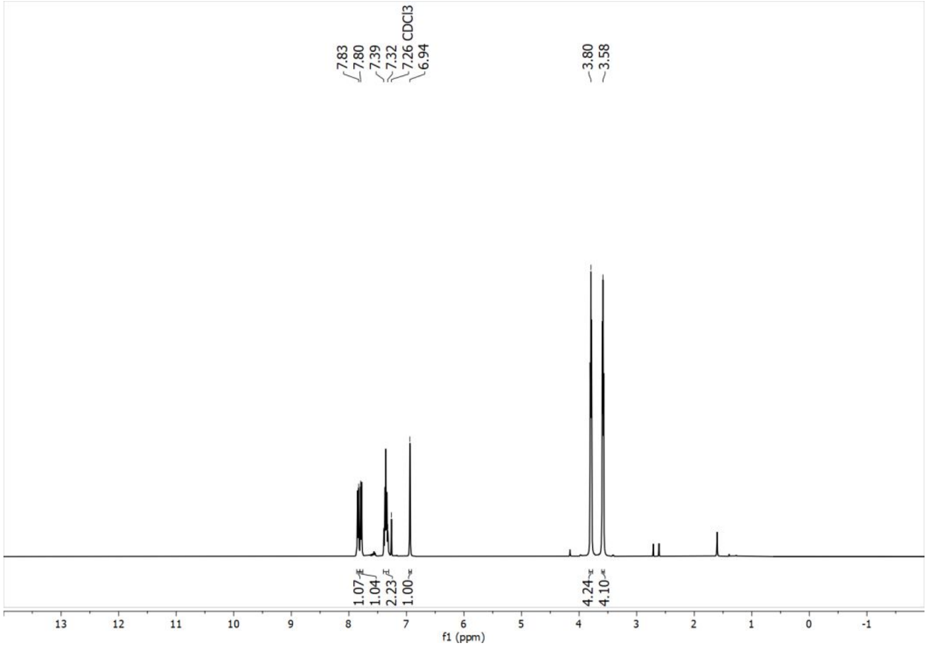

^{13}C NMR: 101 MHz in $CDCl_3$

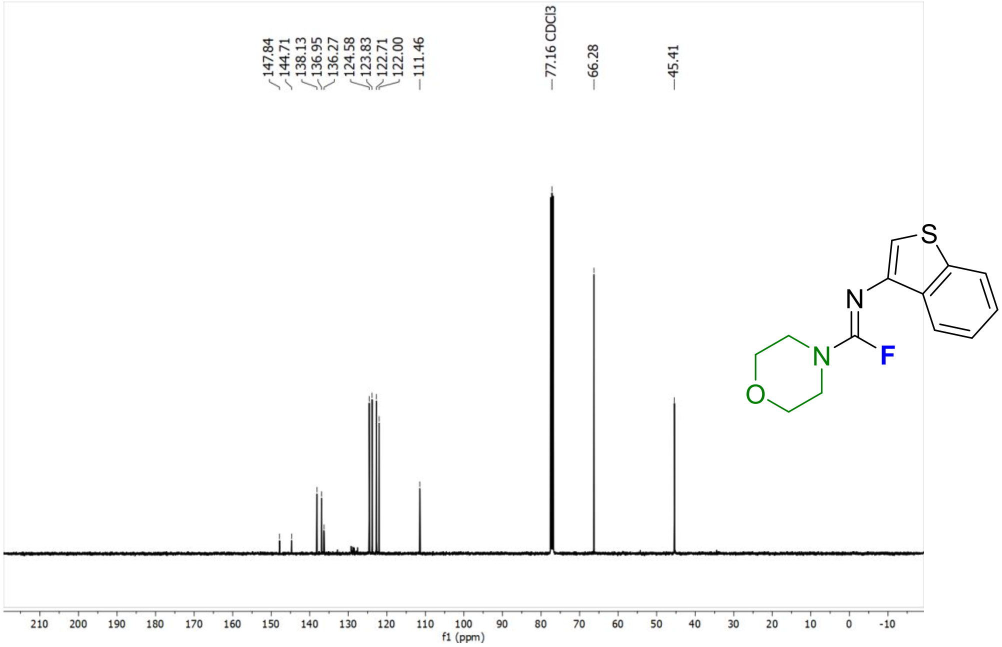

^{19}F NMR: 376 MHz in $CDCl_3$

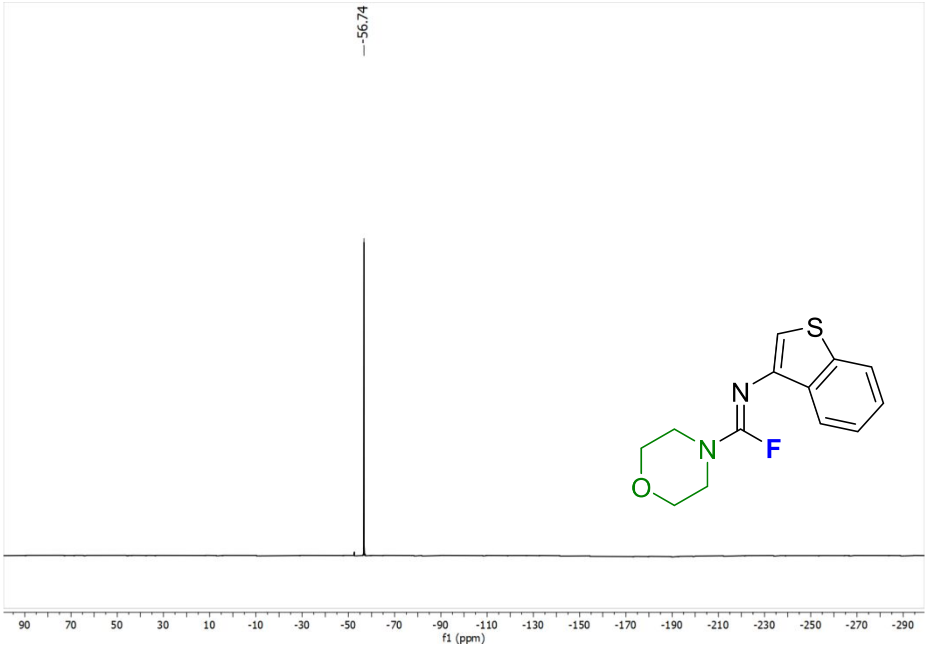

(Z)-N-((E)-styryl)morpholine-4-carbimidoyl fluoride (2p)

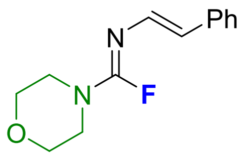

^{1}H NMR: 400 MHz in $CDCl_3$

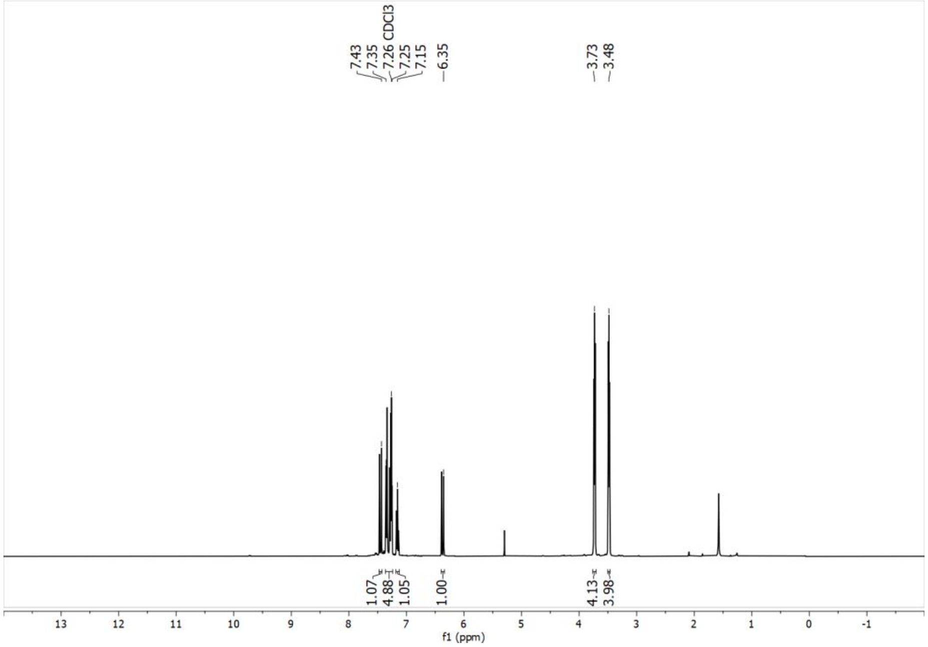

^{13}C NMR: 101 MHz in $CDCl_3$

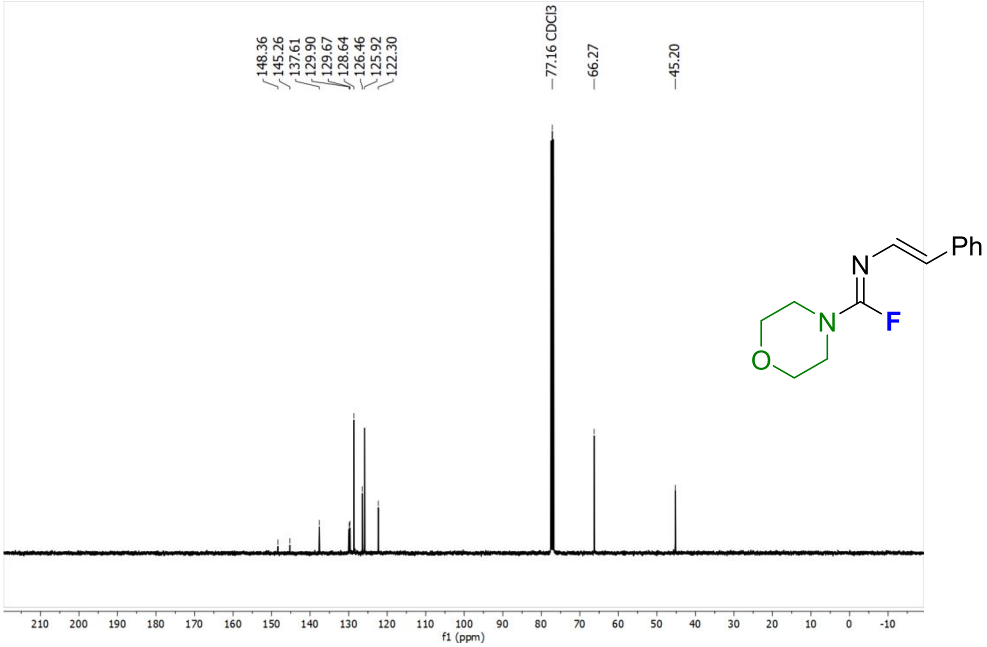

^{19}F NMR: 376 MHz in $CDCl_3$

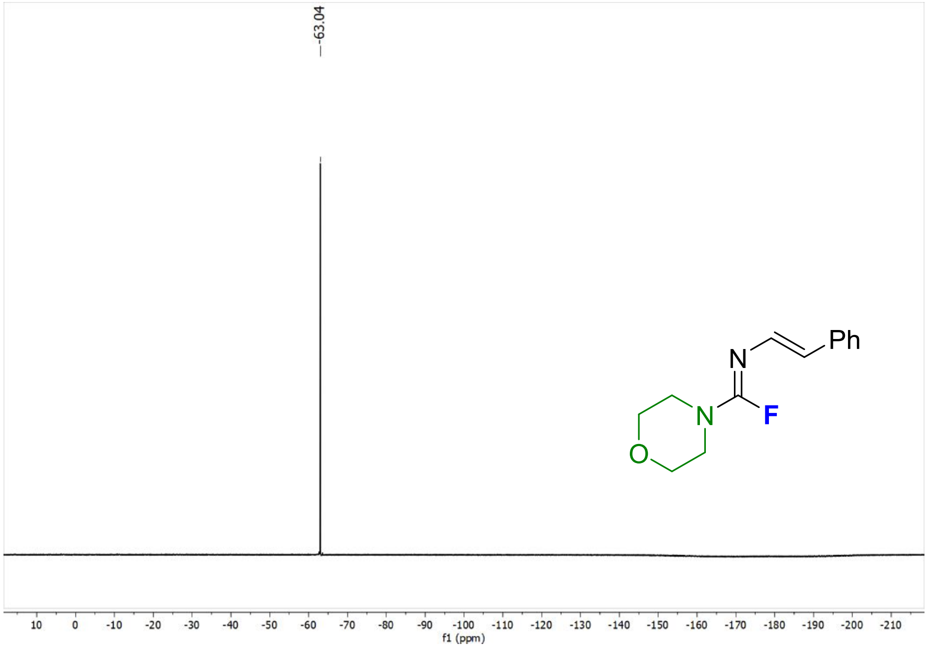

(Z)-N-phenethylmorpholine-4-carbimidoyl fluoride (2q)

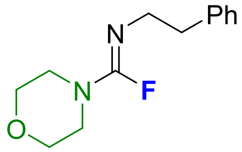

^{19}F NMR: 376 MHz in acetonitrile – crude reaction mixture with 1 eq. of 4-fluoroanisole (-125.30 ppm) as internal standard. Product peak is present at -61.2 ppm (23%).

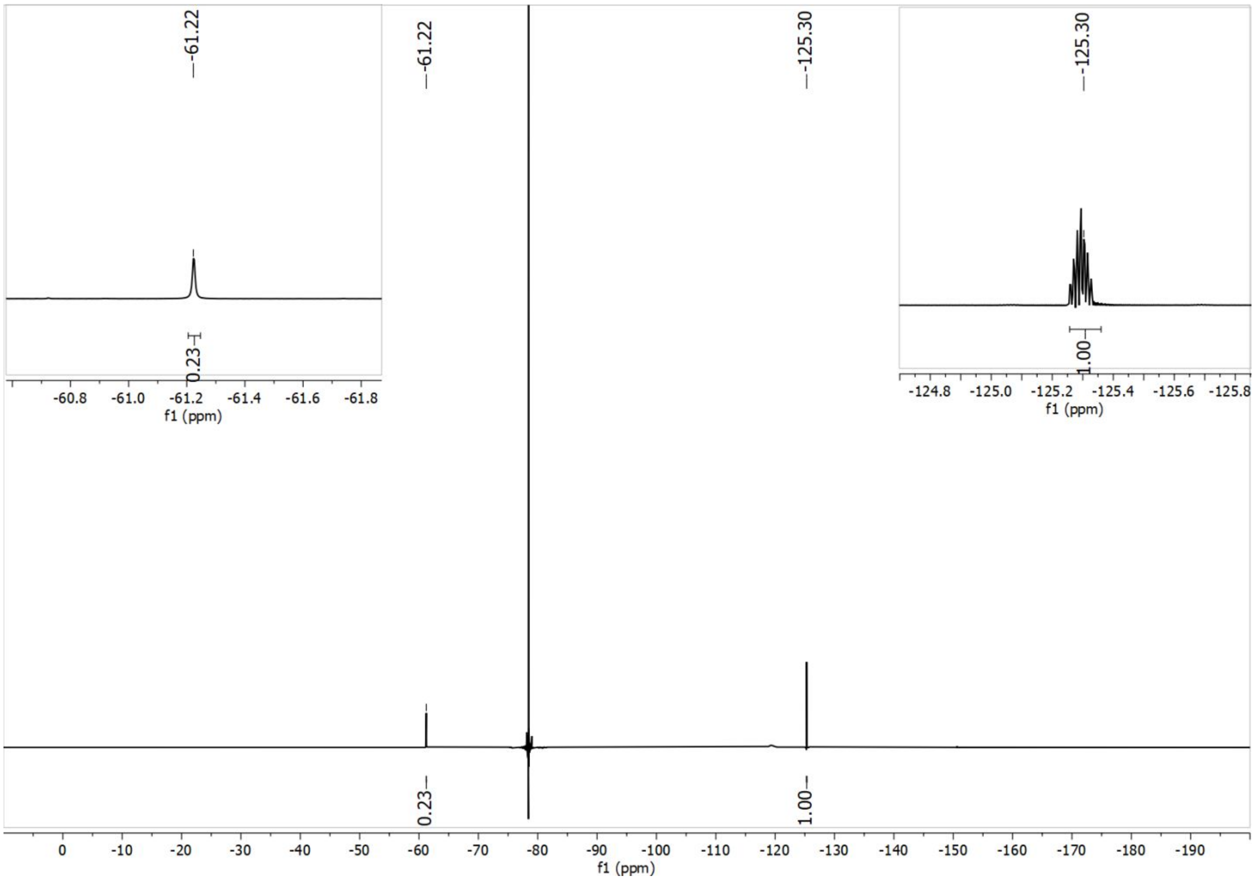

(Z)-N,4-diphenylpiperazine-1-carbimidoyl fluoride (2r)

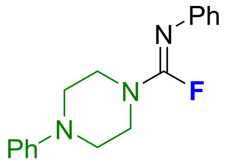

^{1}H NMR: 400 MHz in $CDCl_3$

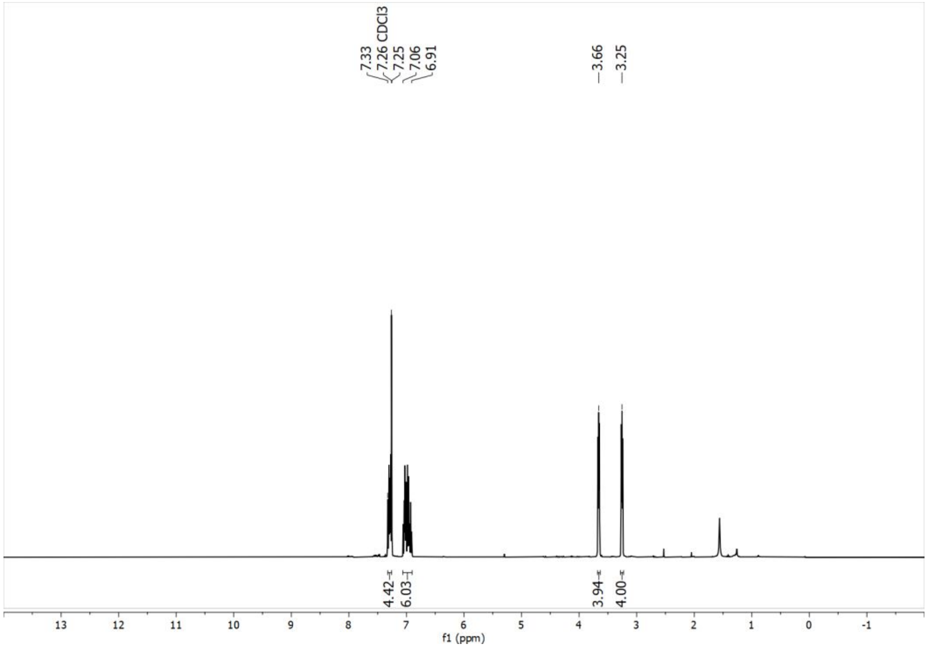

^{13}C NMR: 101 MHz in $CDCl_3$

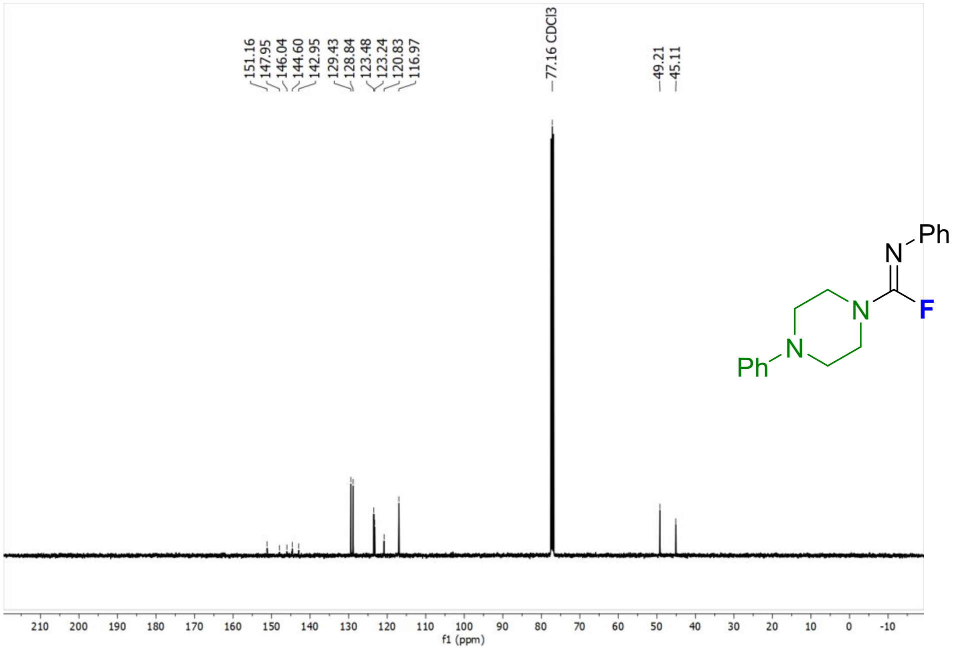

^{19}F NMR: 376 MHz in $CDCl_3$

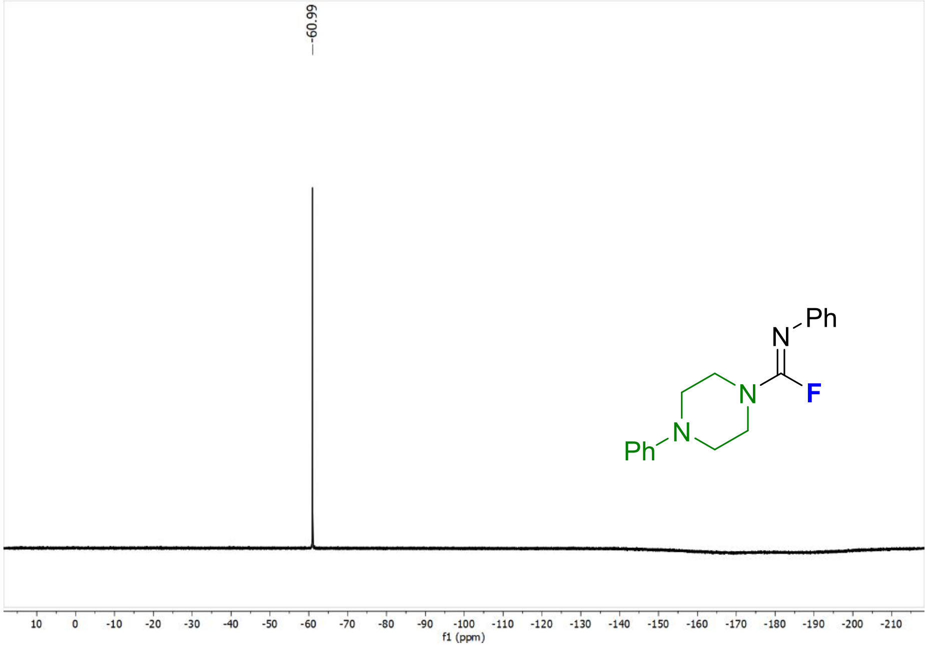

benzyl (Z)-4-(fluoro(phenylimino)methyl)-1,4-diazepane-1-carboxylate (2s)

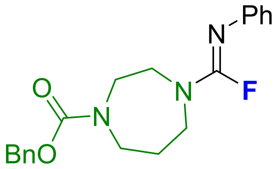

^{1}H NMR: 400 MHz in $CDCl_3$

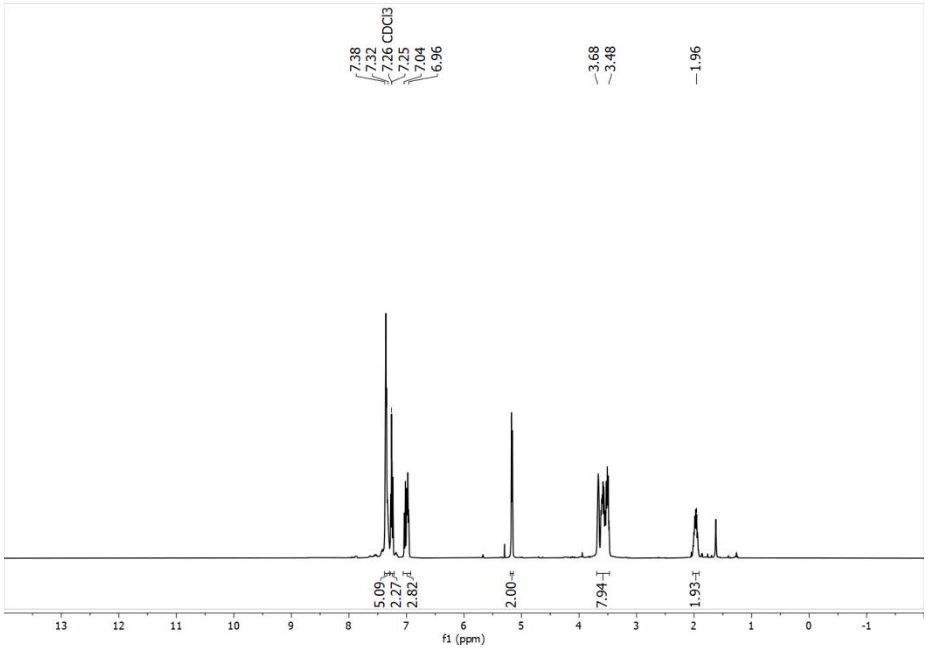

^{13}C NMR: 101 MHz in $CDCl_3$

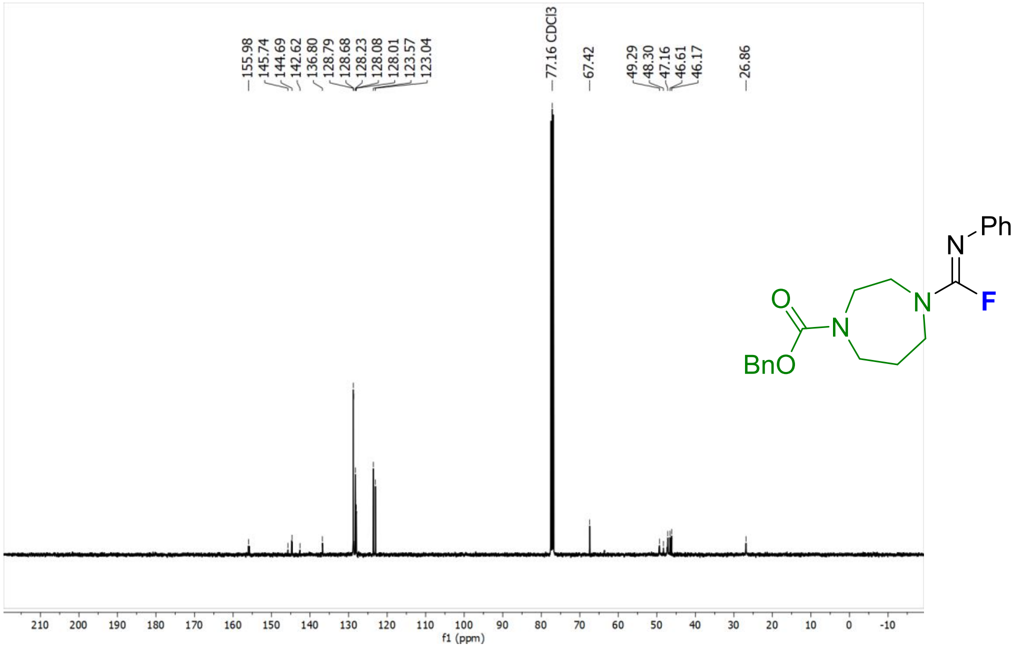

^{19}F NMR: 376 MHz in $CDCl_3$

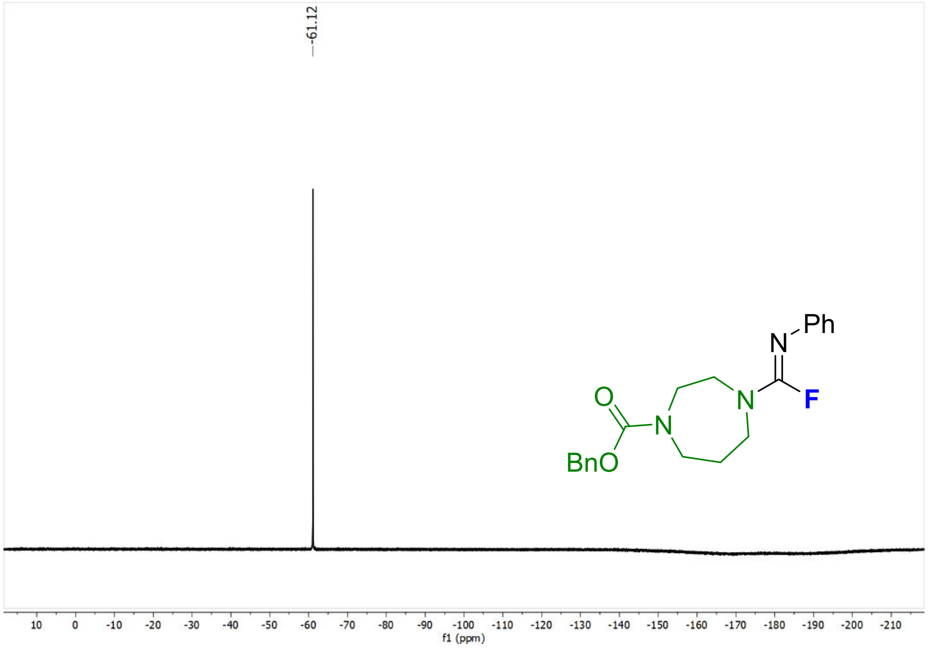

(Z)-N-phenyl-1,4-dioxa-8-azaspiro[4.5]decane-8-carbimidoyl fluoride (2t)

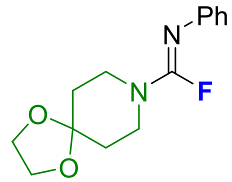

^{1}H NMR: 400 MHz in $CDCl_3$

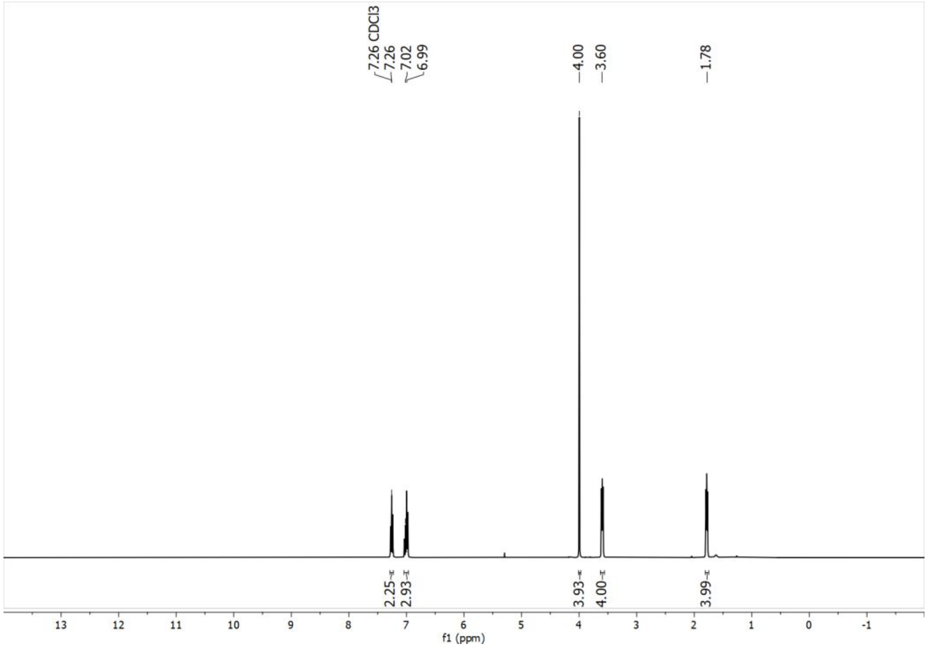

^{13}C NMR: 101 MHz in $CDCl_3$

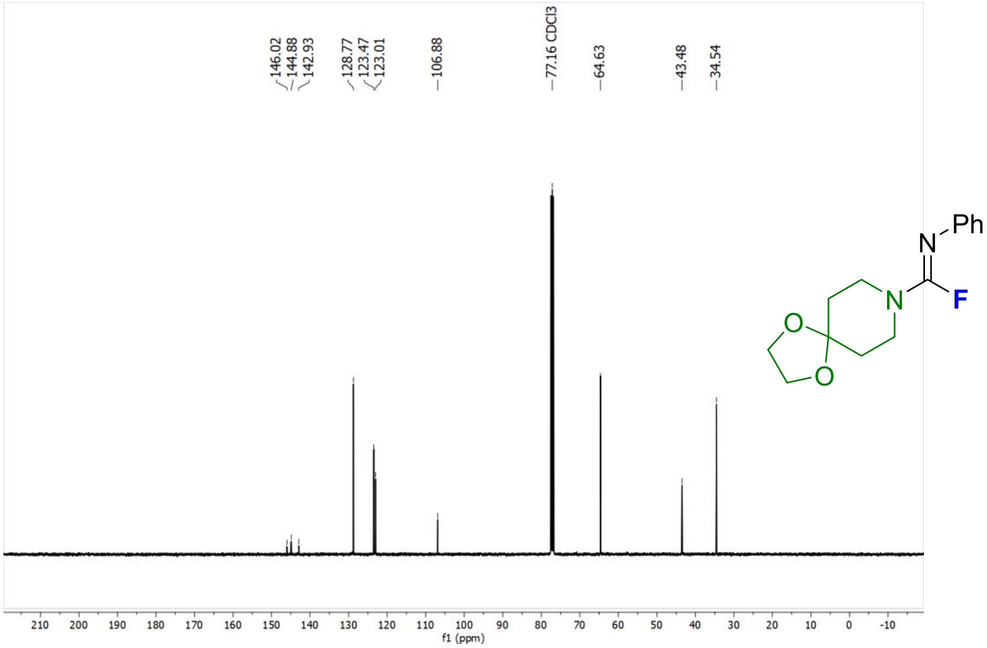

^{19}F NMR: 376 MHz in $CDCl_3$

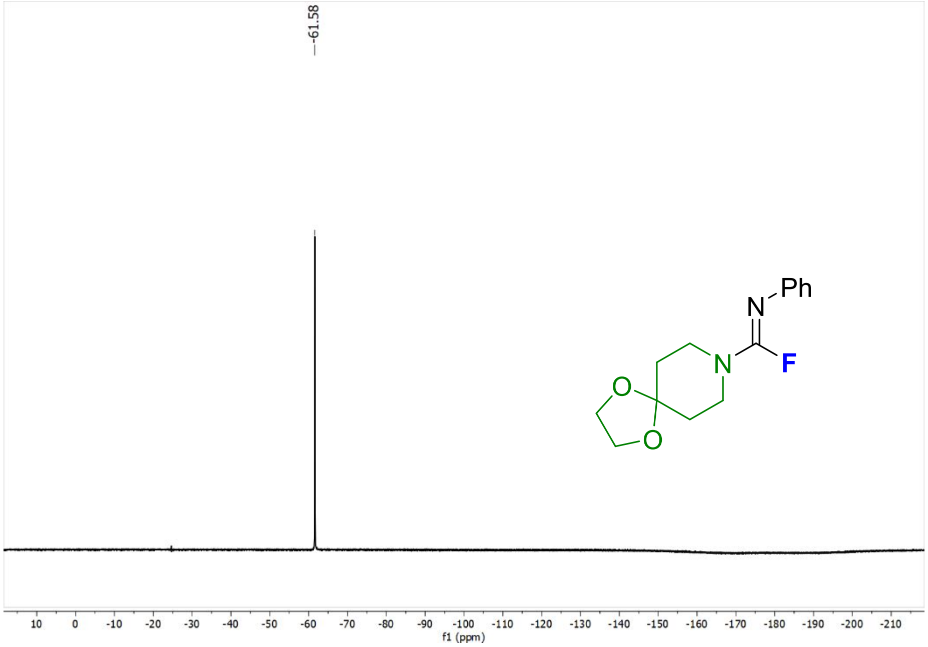

(Z)-6-bromo-N-phenyl-3,4-dihydroisoquinoline-2(1H)-carbimidoyl fluoride (2u)

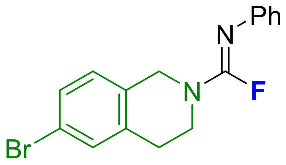

^{1}H NMR: 400 MHz in $CDCl_3$

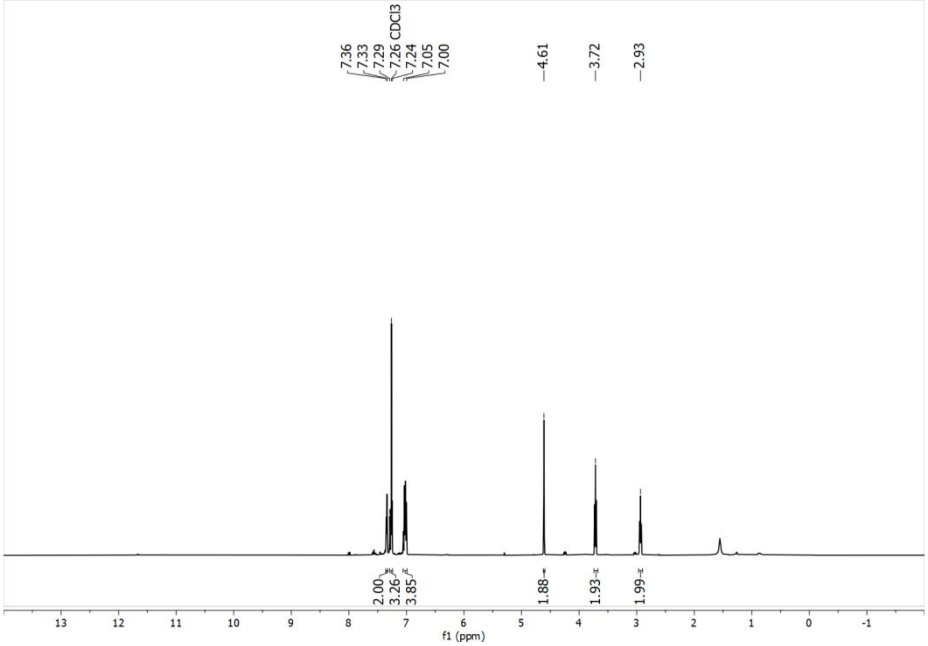

^{13}C NMR: 101 MHz in $CDCl_3$

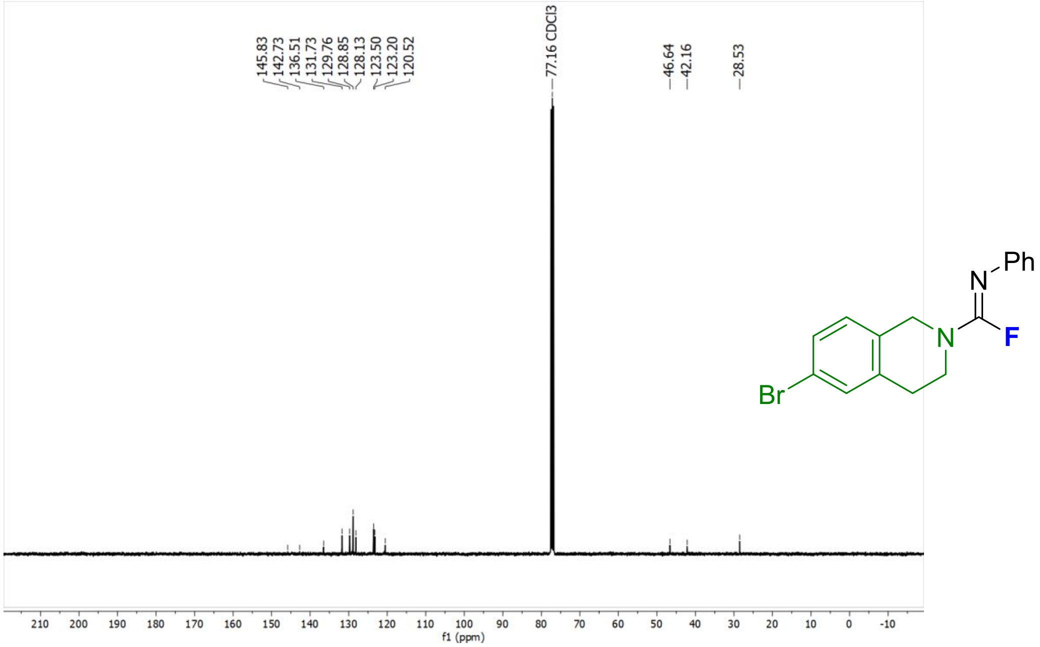

^{19}F NMR: 376 MHz in $CDCl_3$

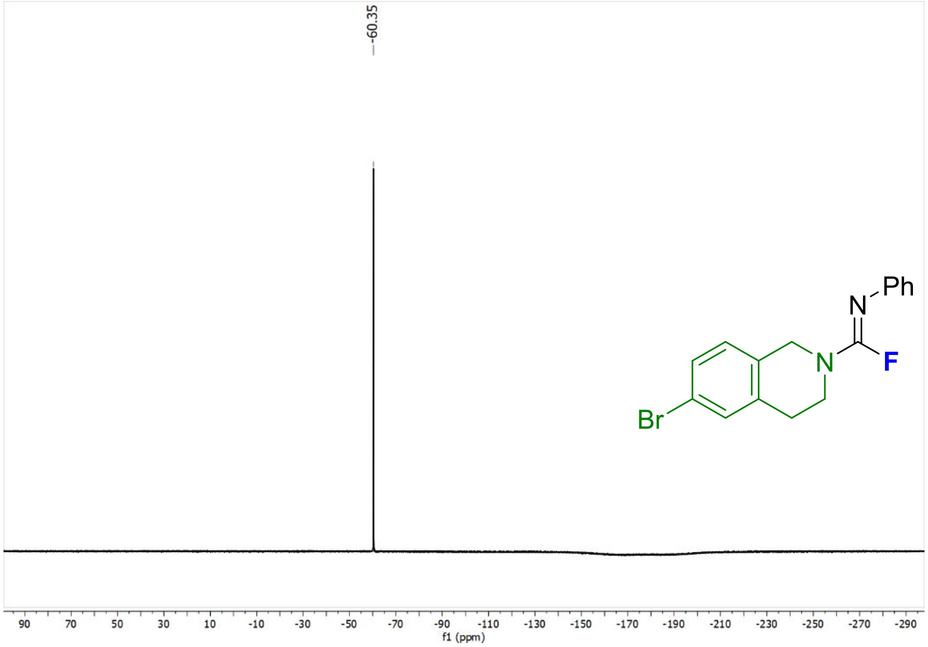

(Z)-2-methyl-N-phenyl-1H-imidazole-1-carbimidoyl fluoride (2v)

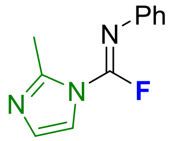

^{1}H NMR: 400 MHz in $CDCl_3$

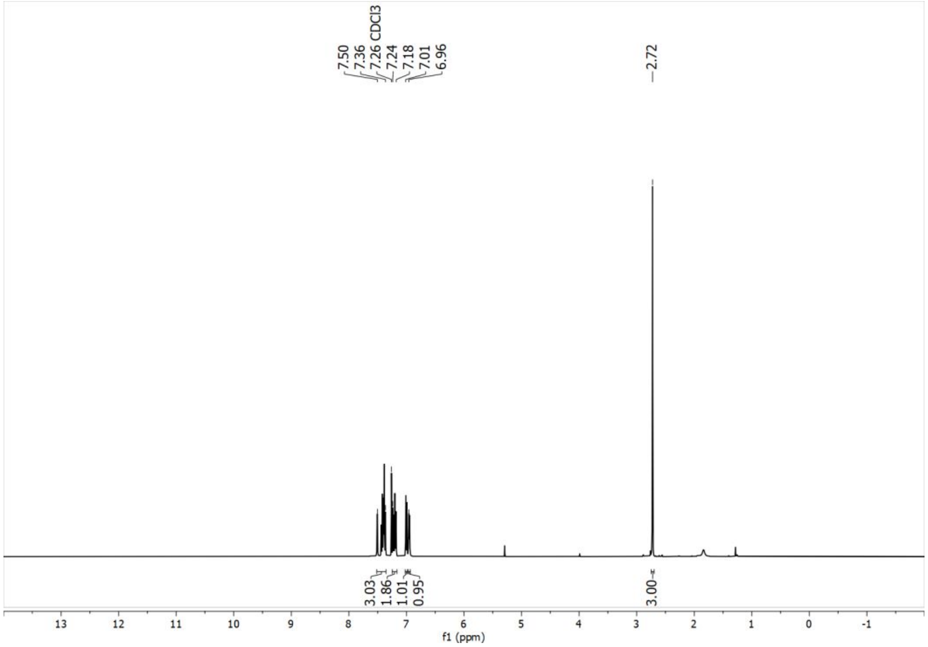

^{13}C NMR: 101 MHz in $CDCl_3$

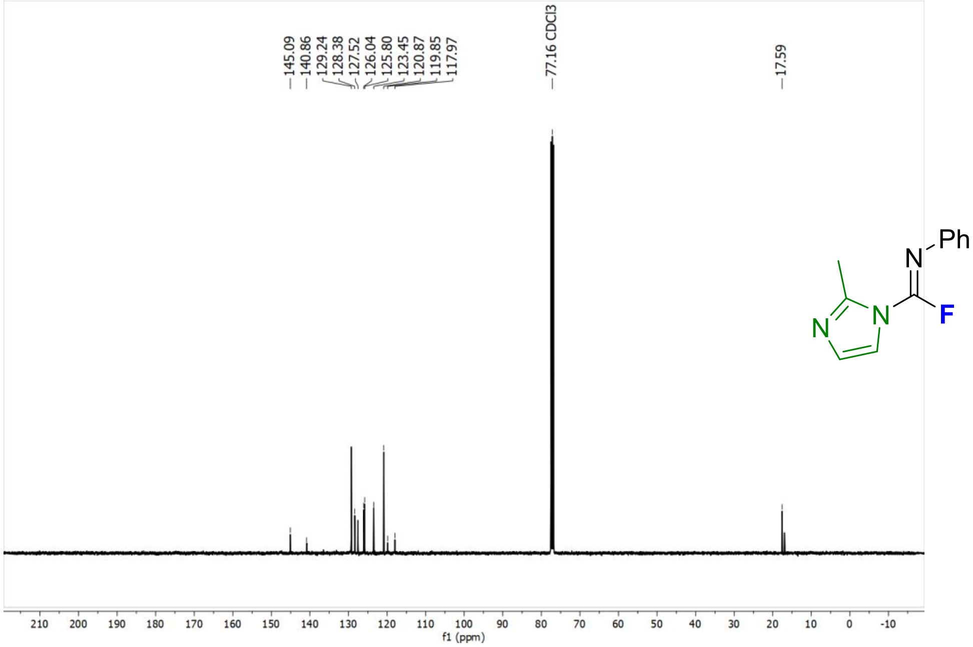

^{19}F NMR: 376 MHz in $CDCl_3$

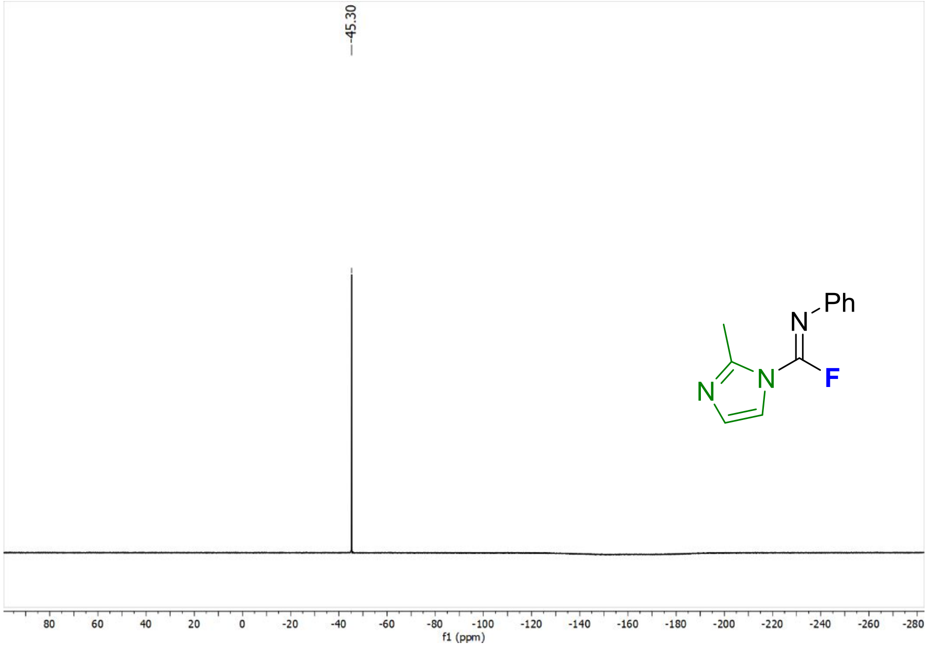

(Z)-N-(bis(dimethylamino)methylene)-N'-phenylcarbamimidic fluoride (2w)

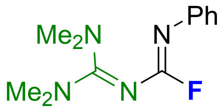

^{19}F NMR: 376 MHz in acetonitrile – crude reaction mixture with 1.0 eq. of 4-fluoroanisole (-125.33 ppm) as internal standard. Product peak is present at -61.10 ppm (33%).

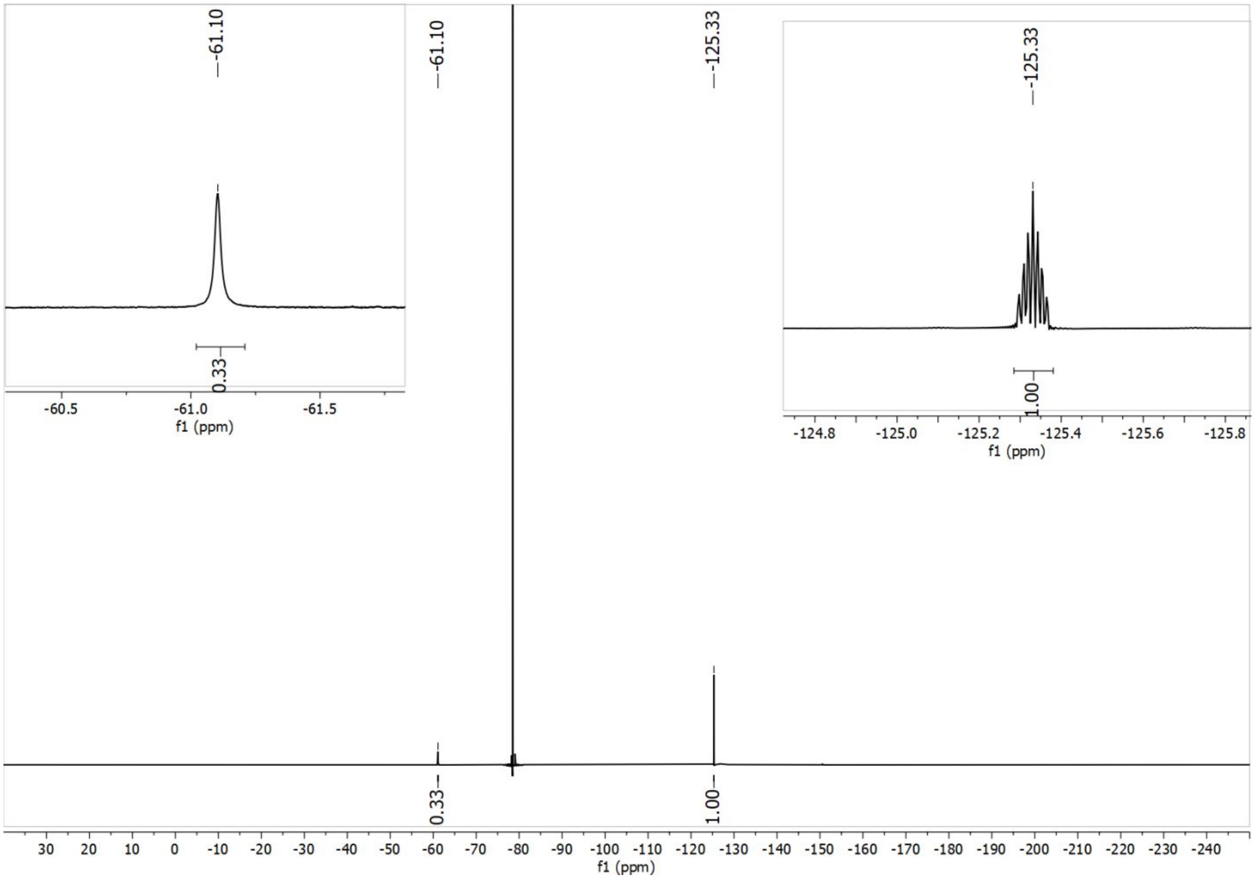

(Z)-N-benzyl-N-methyl-N'-phenylcarbamimidic fluoride (2x)

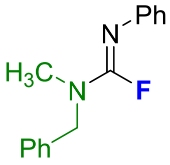

^{1}H NMR: 400 MHz in $CDCl_3$

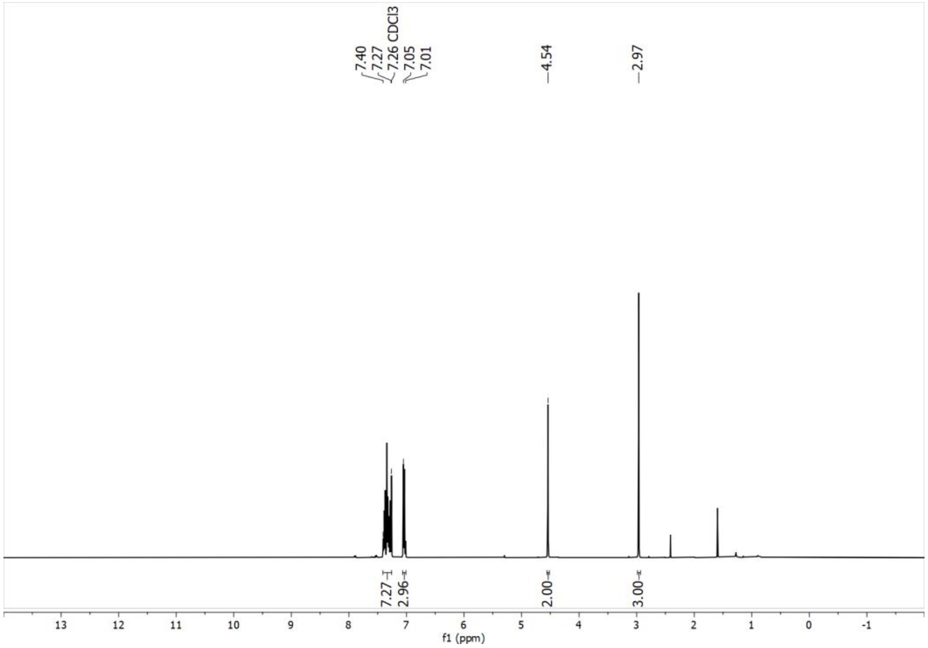

^{13}C NMR: 101 MHz in $CDCl_3$

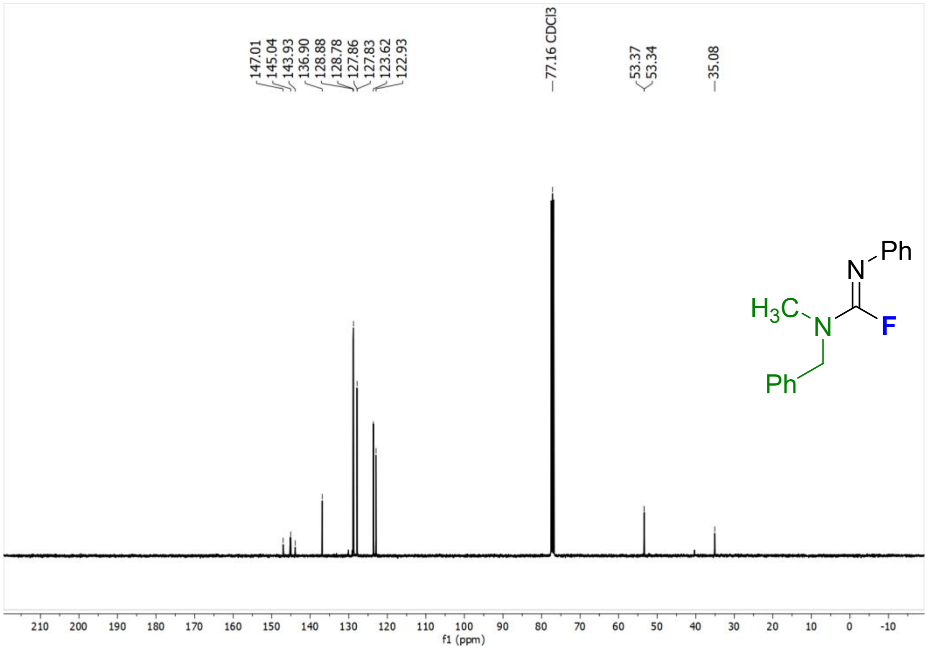

^{19}F NMR: 376 MHz in $CDCl_3$

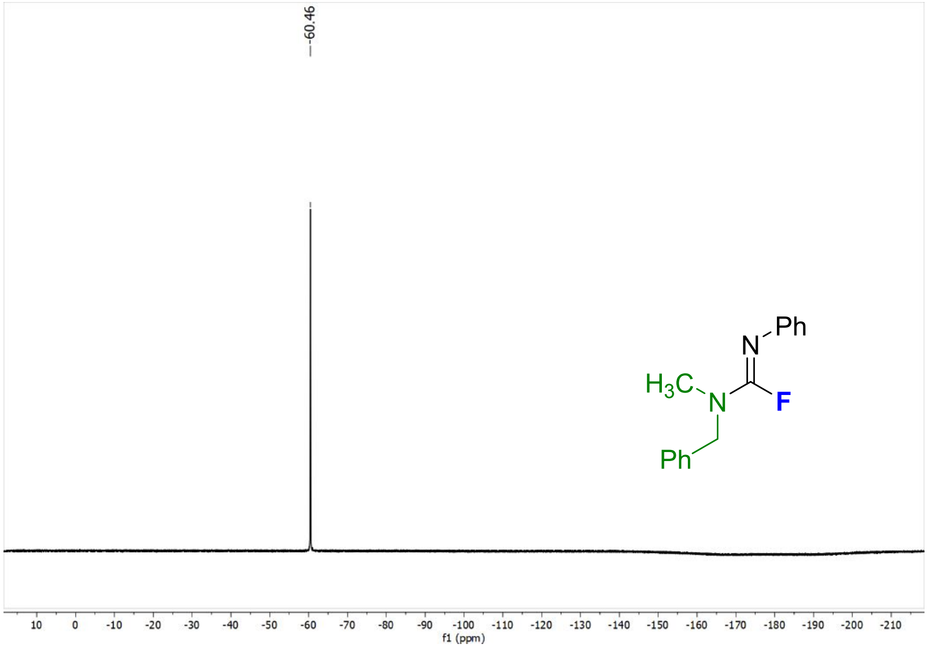

(Z)-N,N-dibenzyl-N'-phenylcarbamimidic fluoride (2y)

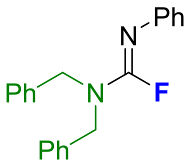

^{1}H NMR: 400 MHz in $CDCl_3$

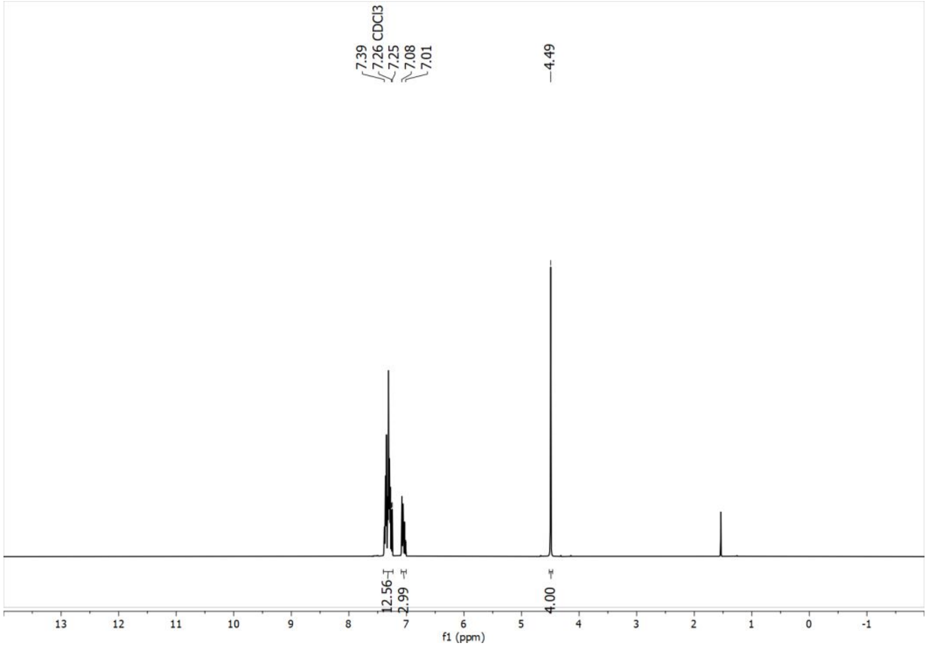

^{13}C NMR: 101 MHz in $CDCl_3$

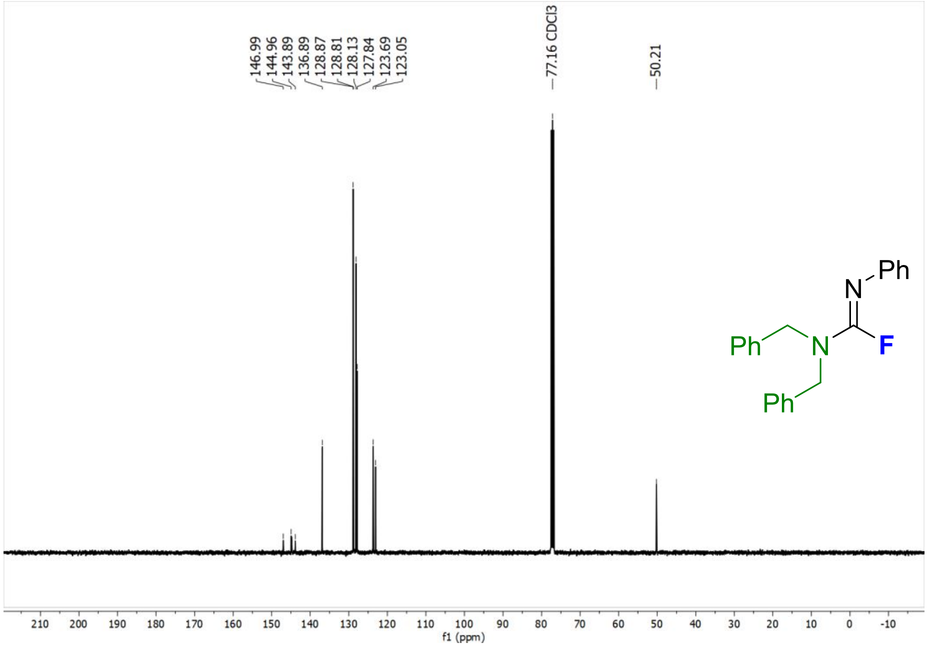

^{19}F NMR: 376 MHz in $CDCl_3$

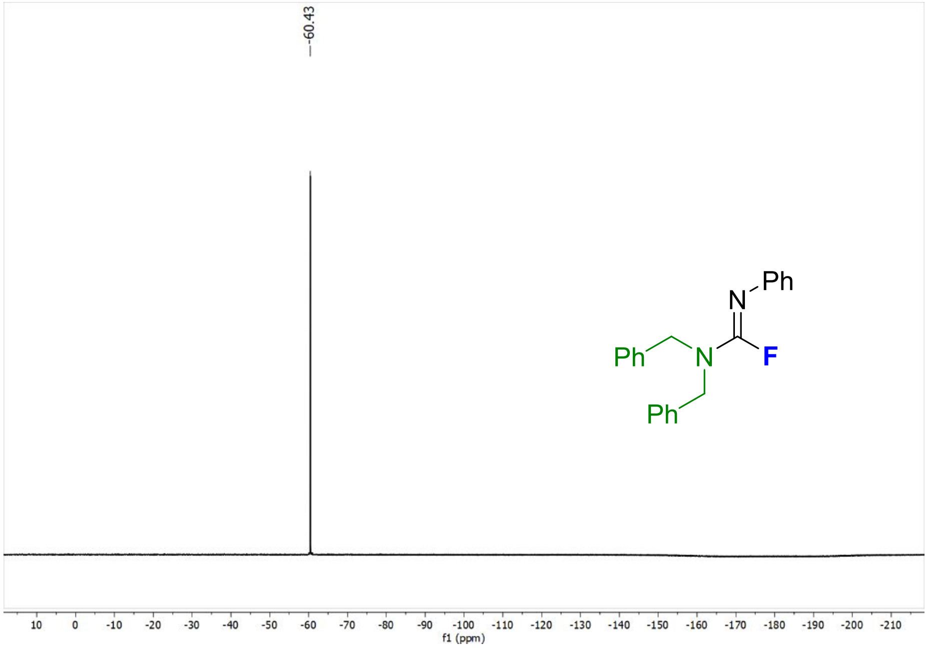

(Z)-N-benzyl-N-isopropyl-N'-phenylcarbamimidic fluoride (2z)

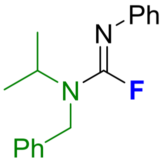

^{1}H NMR: 400 MHz in $CDCl_3$

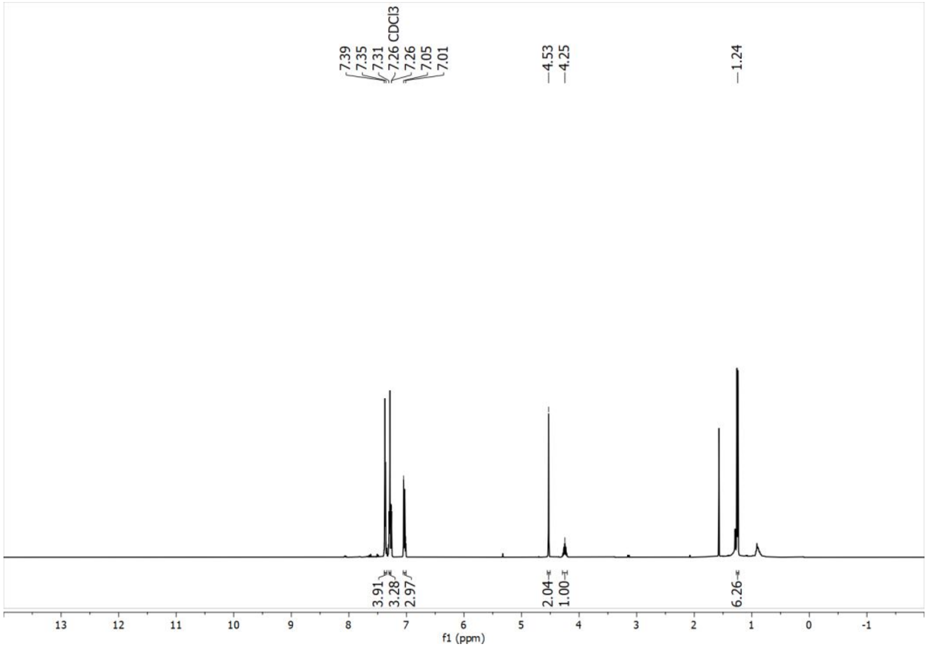

^{13}C NMR: 101 MHz in $CDCl_3$

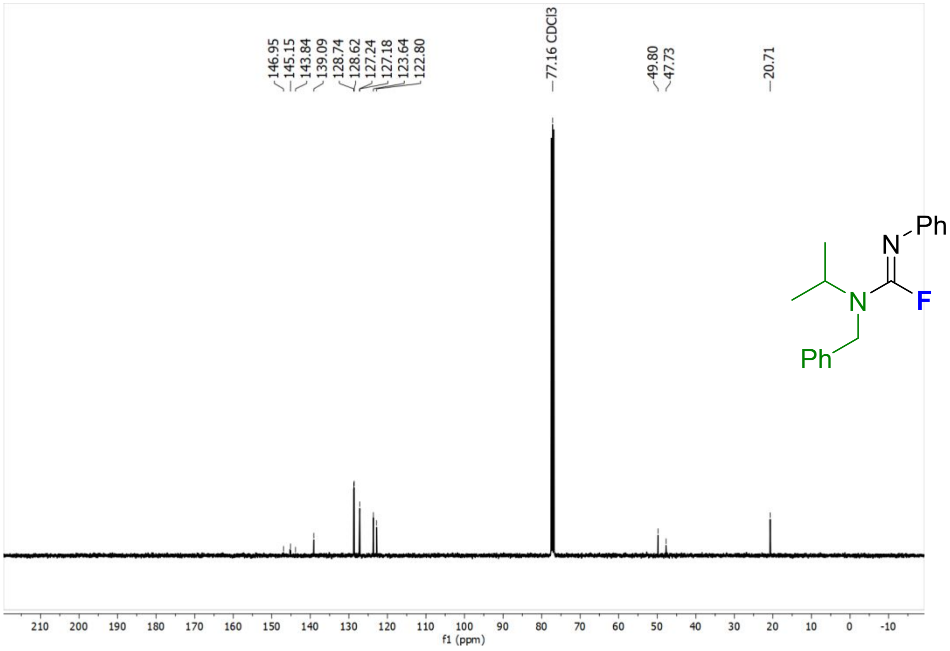

^{19}F NMR: 376 MHz in $CDCl_3$

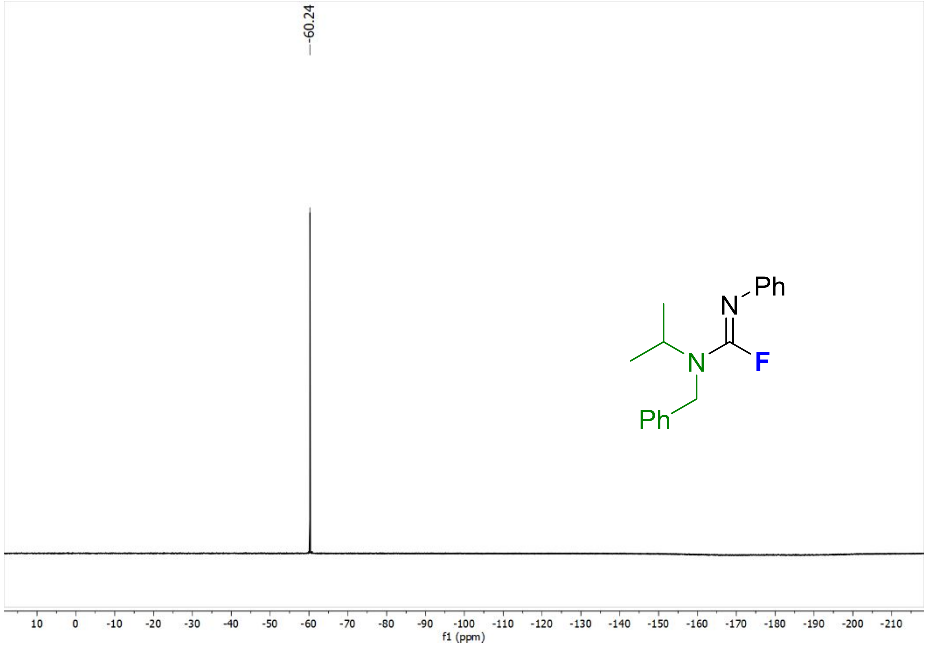

N-phenylmorpholine-4-carboxamide (3a)

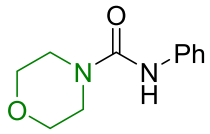

^{1}H NMR: 400 MHz in $CDCl_3$

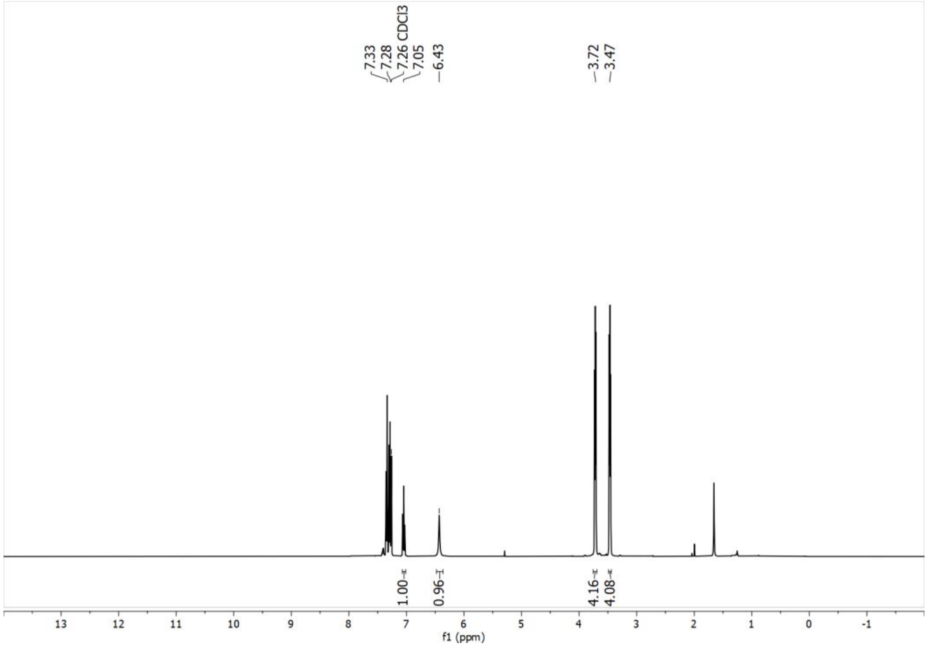

^{13}C NMR: 101 MHz in $CDCl_3$

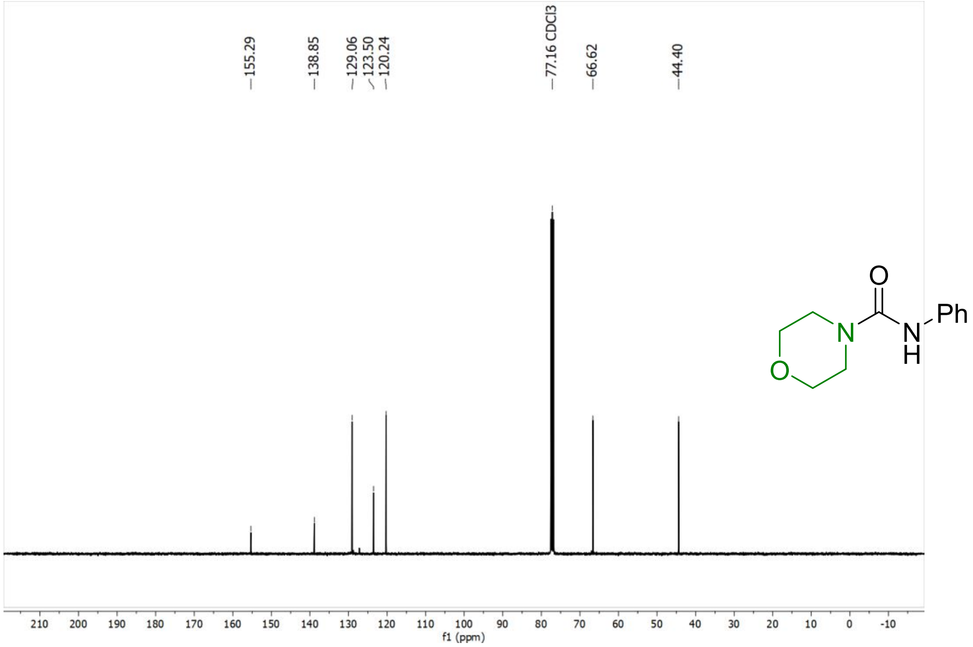

N-(4-methoxyphenyl)morpholine-4-carboxamide (3b)

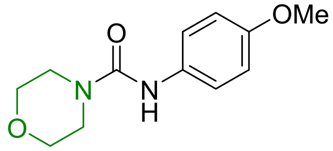

^{1}H NMR: 400 MHz in $CDCl_3$

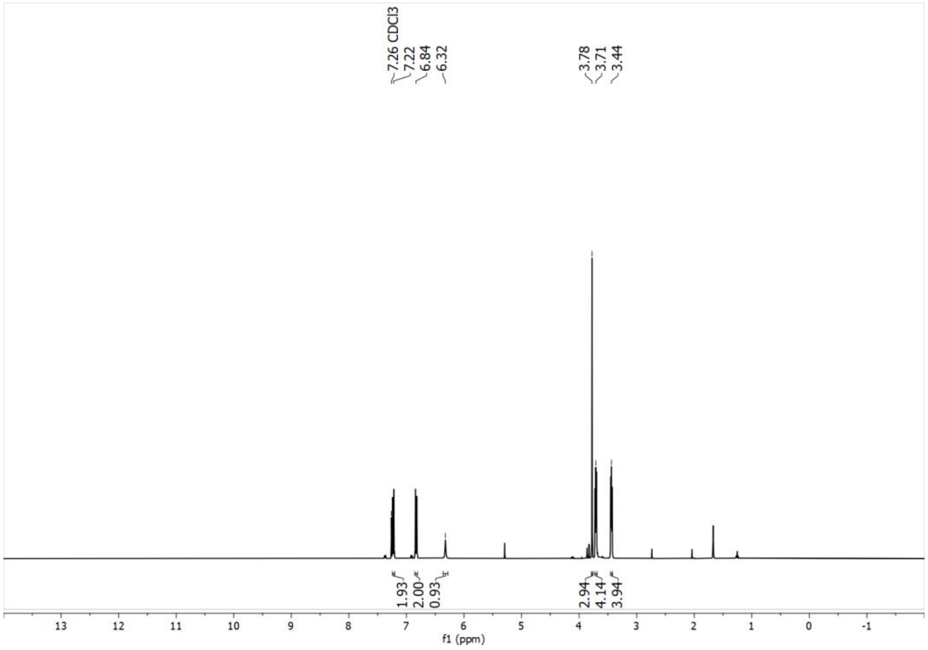

^{13}C NMR: 101 MHz in $CDCl_3$

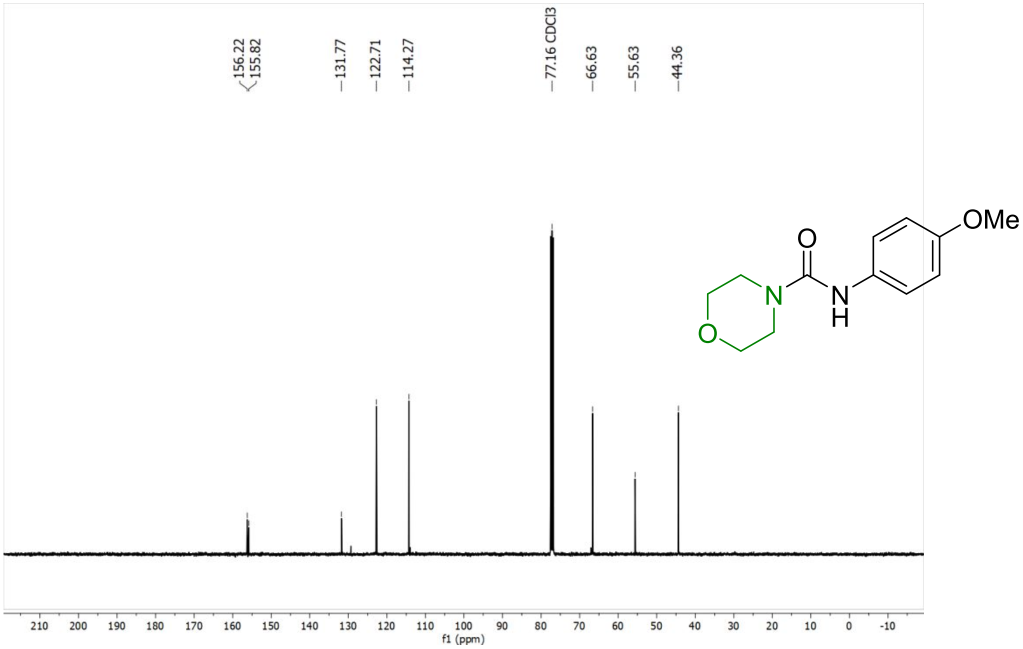

N-(4-(trifluoromethyl)phenyl)morpholine-4-carboxamide (3c)

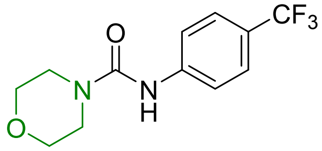

^{1}H NMR: 400 MHz in $CDCl_3$

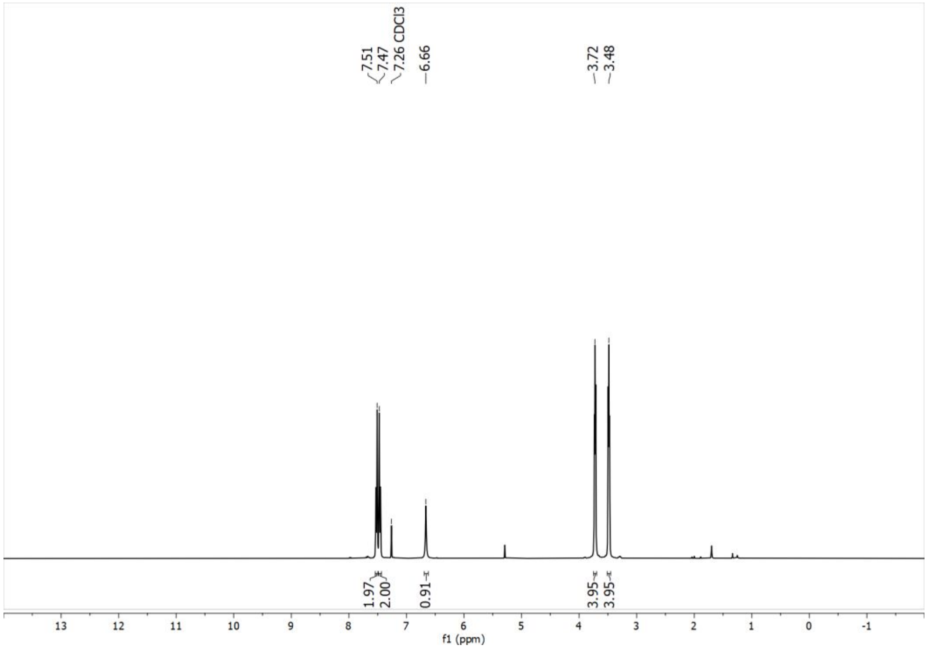

^{13}C NMR: 101 MHz in $CDCl_3$

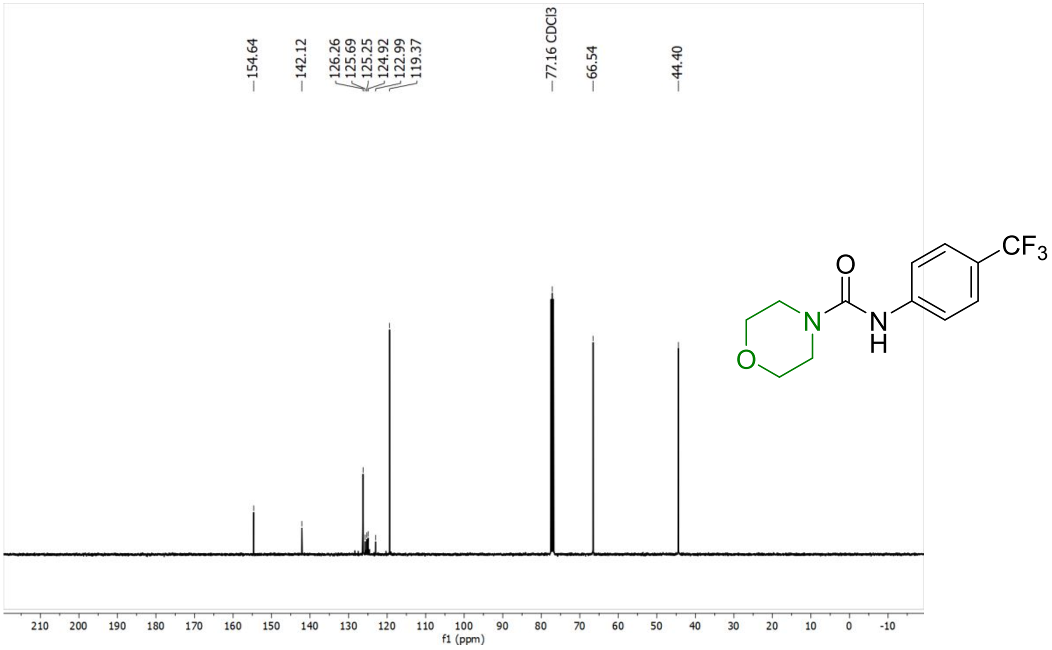

^{19}F NMR: 376 MHz in $CDCl_3$

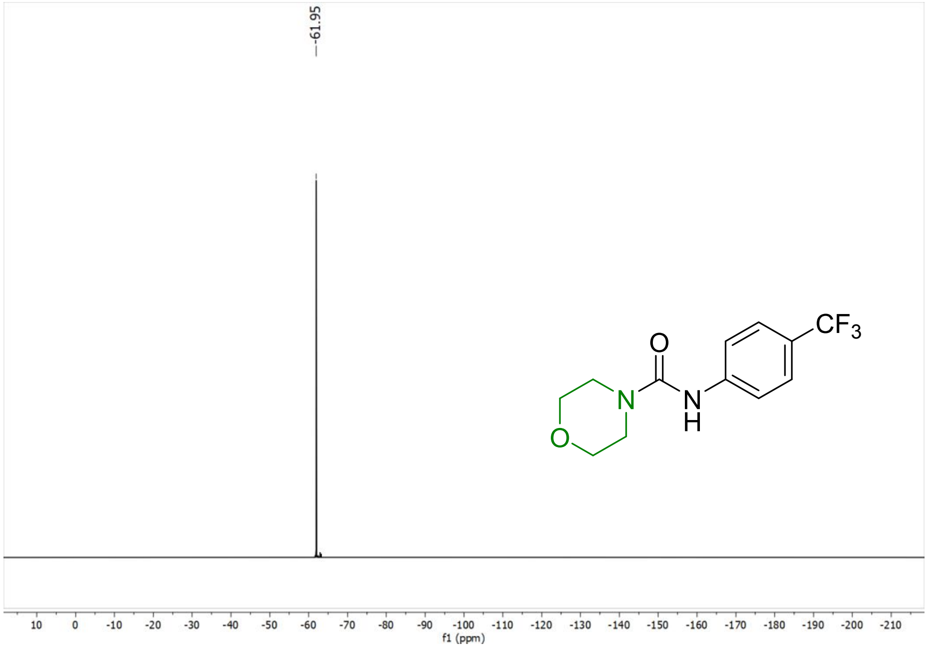

(E)-N-styrylmorpholine-4-carboxamide (3d)

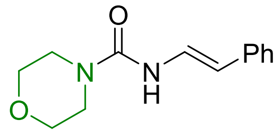

^{1}H NMR: 400 MHz in $CDCl_3$

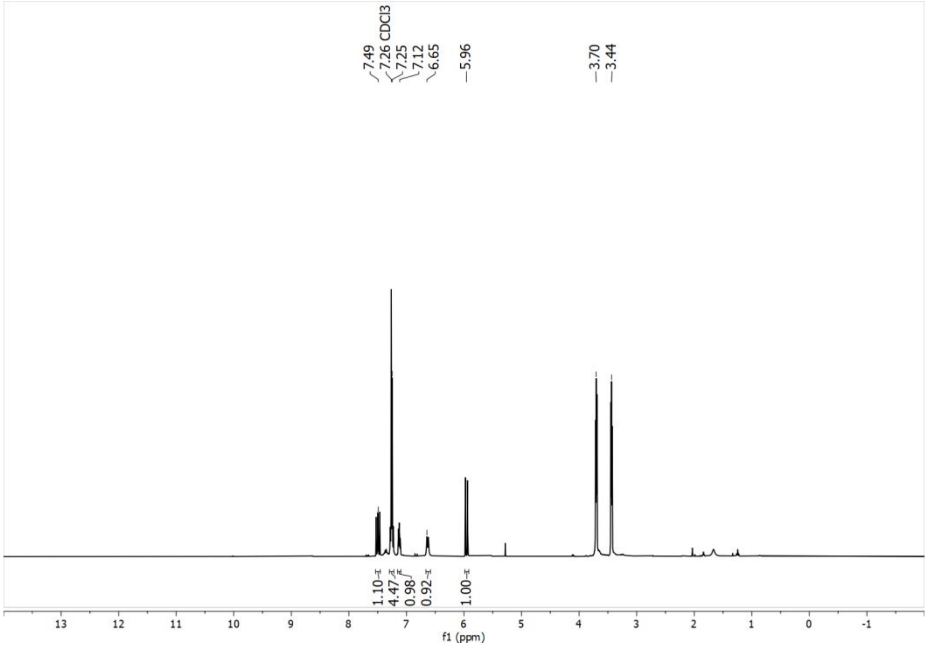

^{13}C NMR: 101 MHz in $CDCl_3$

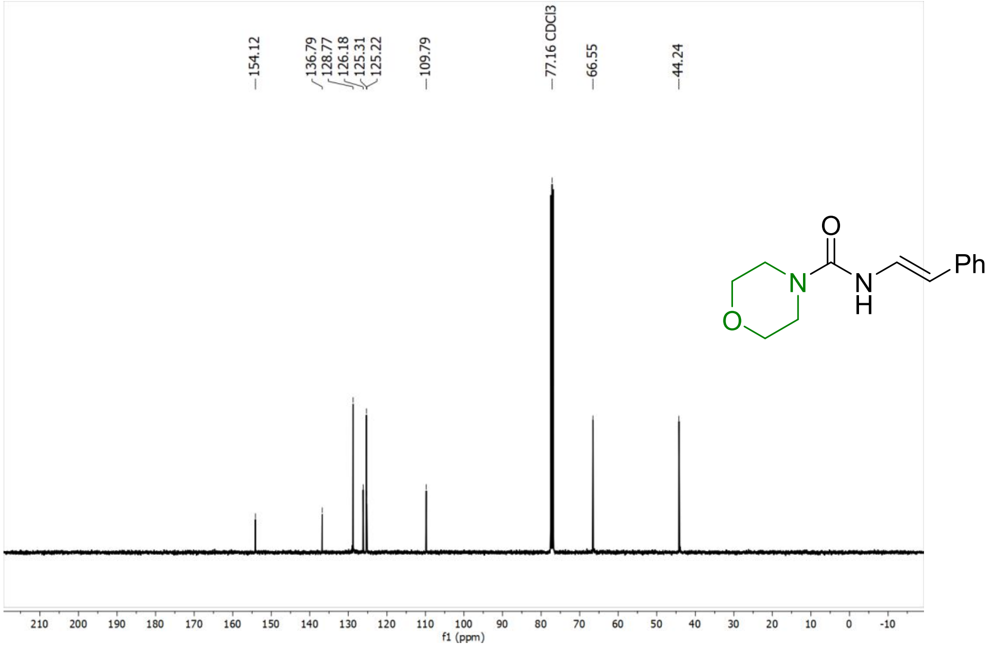

N-phenyl-1,4-dioxa-8-azaspiro[4.5]decane-8-carboxamide (3e)

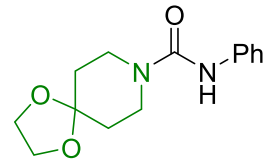

^{1}H NMR: 400 MHz in $CDCl_3$

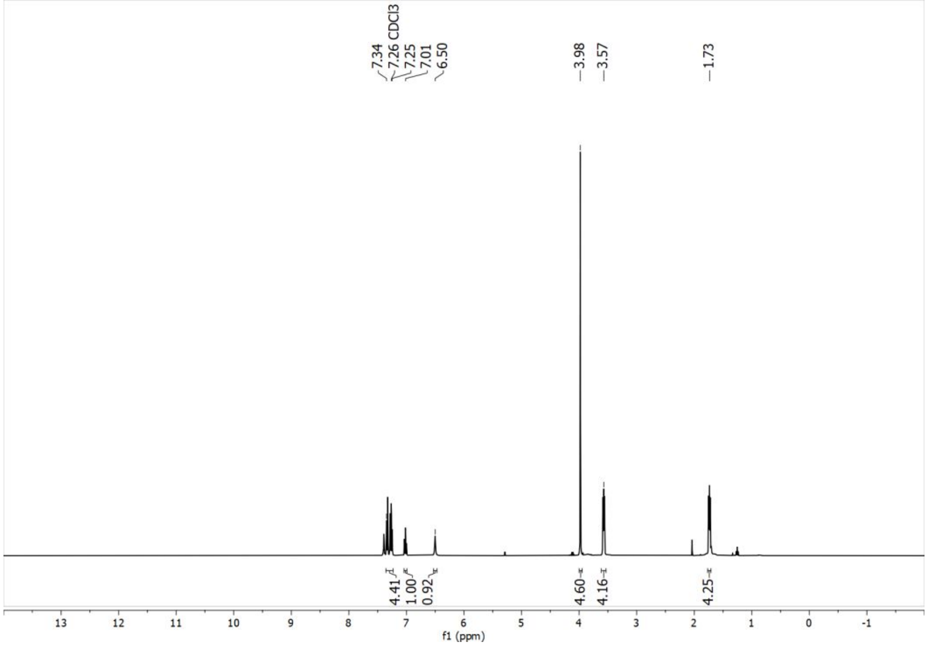

^{13}C NMR: 101 MHz in $CDCl_3$

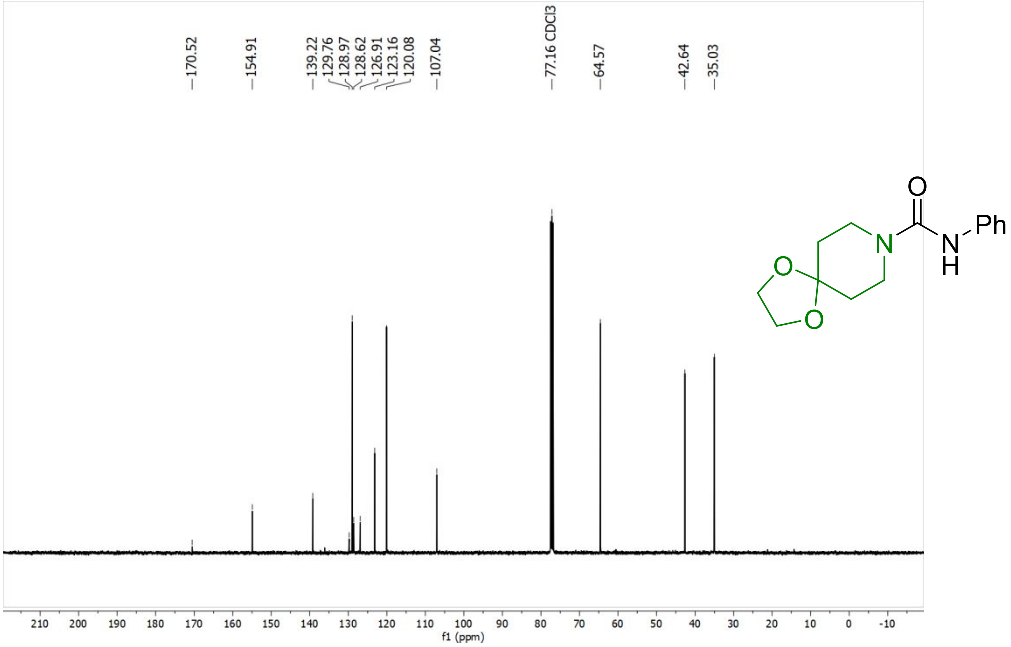

N[1]-phenylpiperidine-1,3-dicarboxamide (3f)

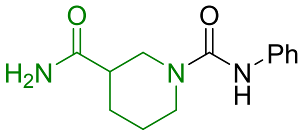

^{1}H NMR: 400 MHz in $CDCl_3$

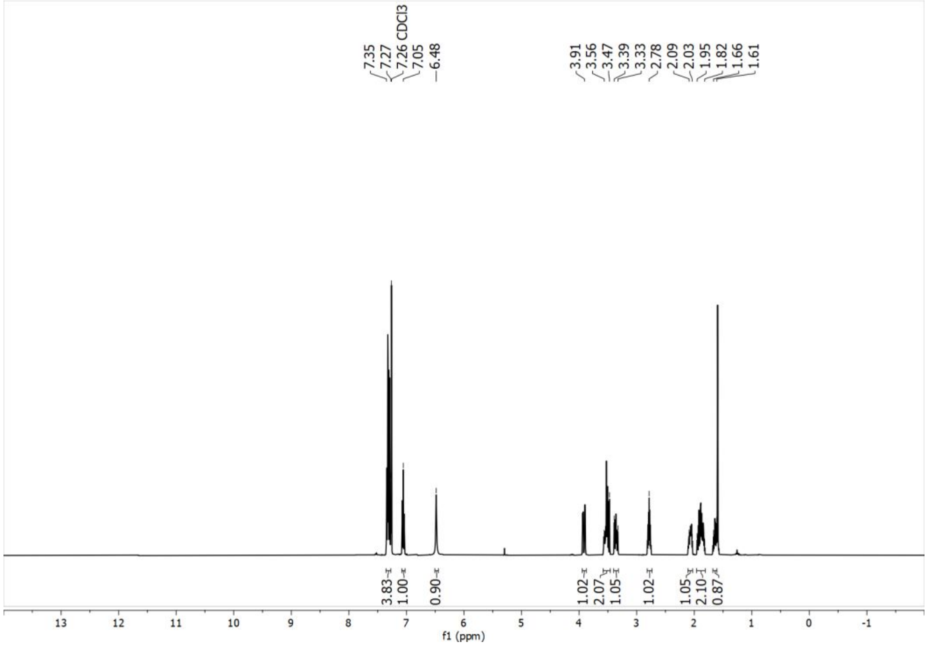

^{13}C NMR: 101 MHz in $CDCl_3$

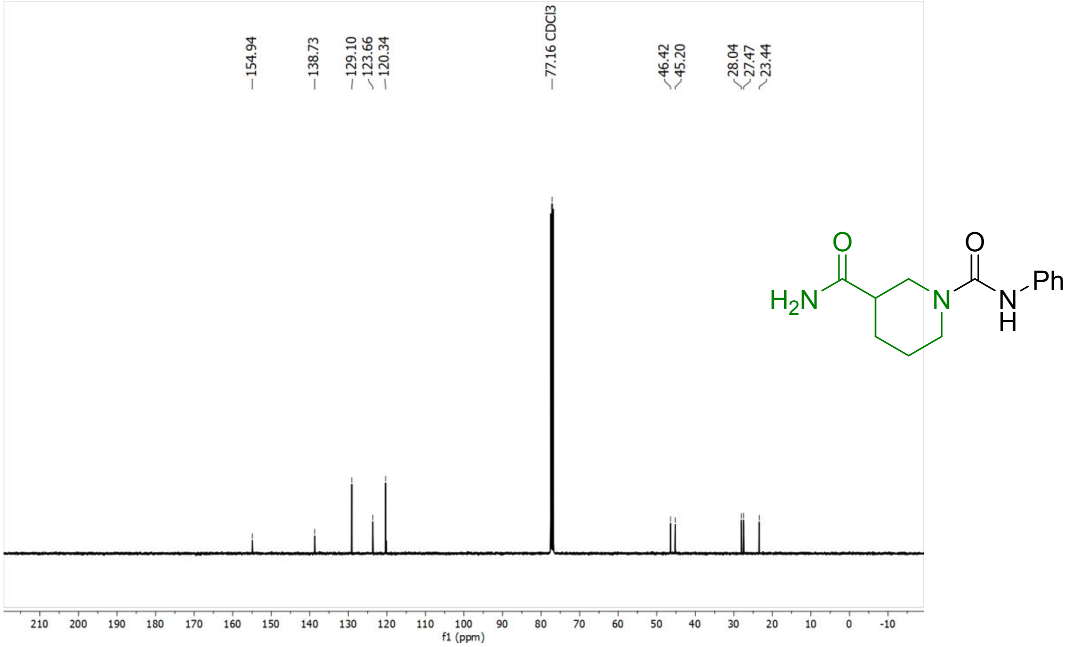

1-benzyl-1-methyl-3-phenylurea (3g)

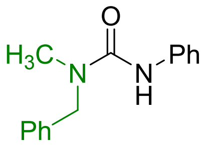

^{1}H NMR: 400 MHz in $CDCl_3$

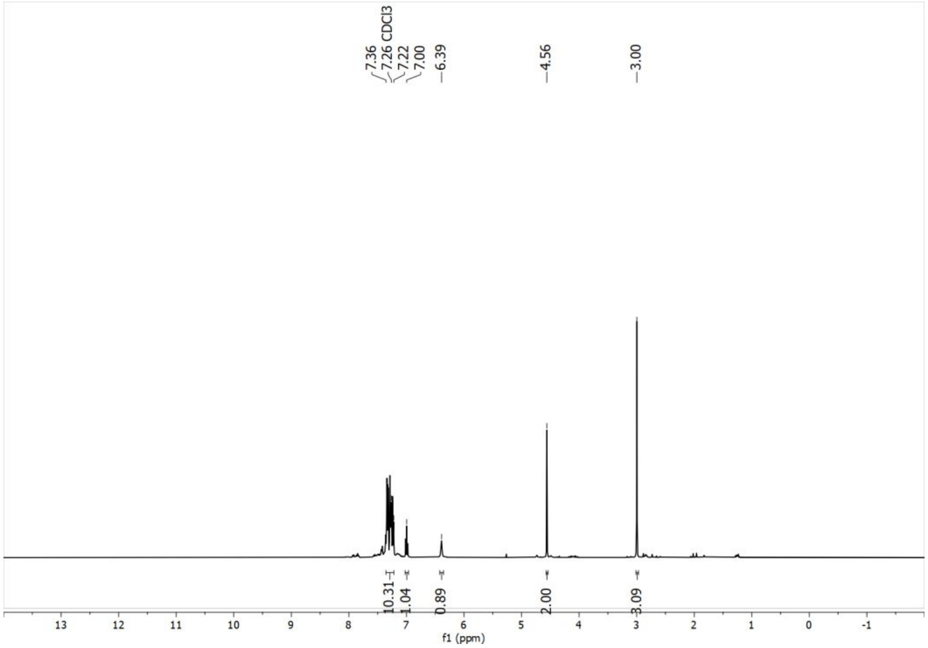

^{13}C NMR: 101 MHz in $CDCl_3$

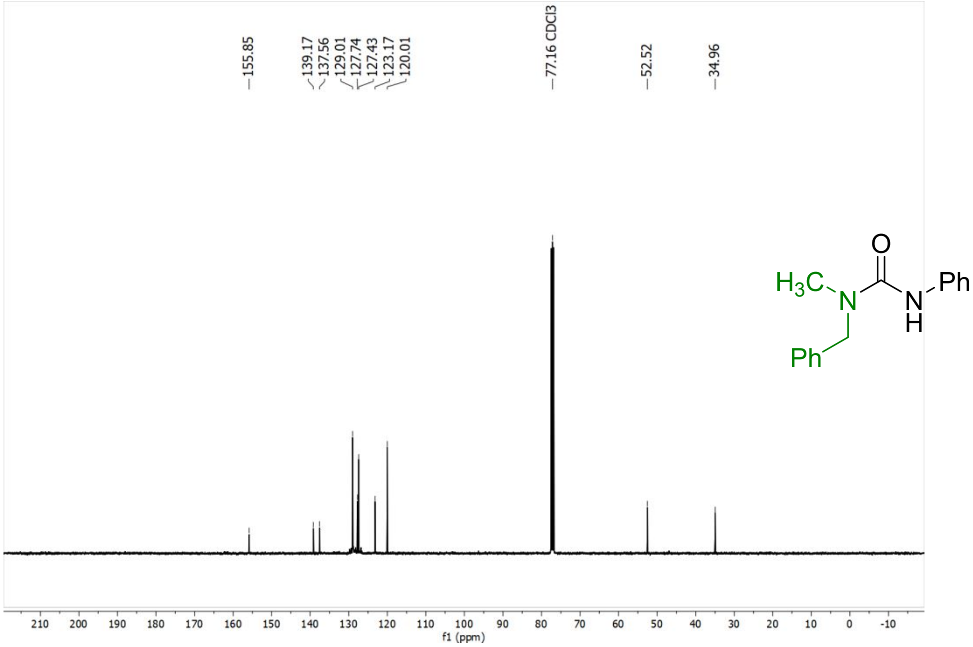

phenyl (Z)-N-phenylmorpholine-4-carbimidate (4a)

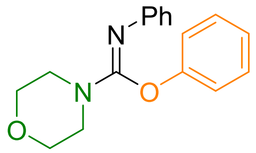

^1H NMR: 400 MHz in $CDCl_3$

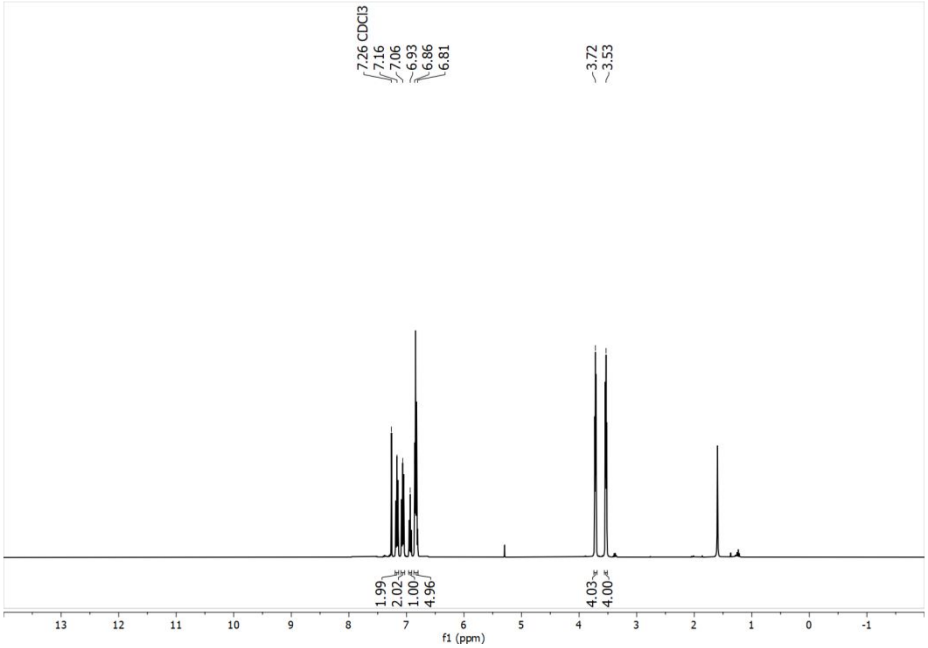

^{13}C NMR: 101 MHz in $CDCl_3$

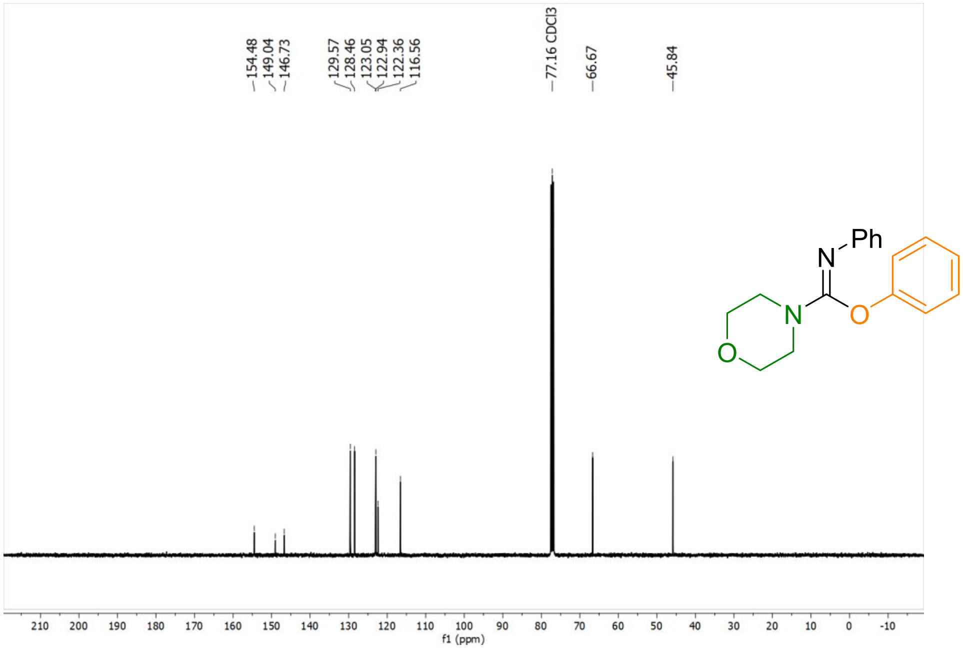

4-methoxyphenyl (Z)-N-phenylmorpholine-4-carbimidate (4b)

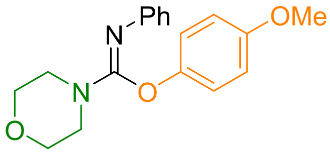

^{1}H NMR: 400 MHz in $CDCl_3$

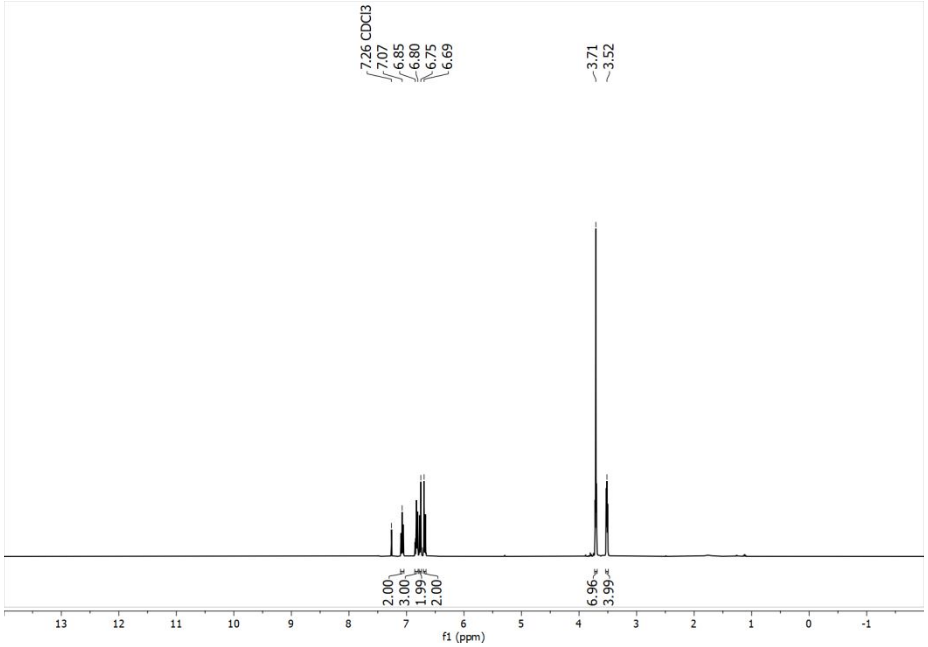

^{13}C NMR: 101 MHz in $CDCl_3$

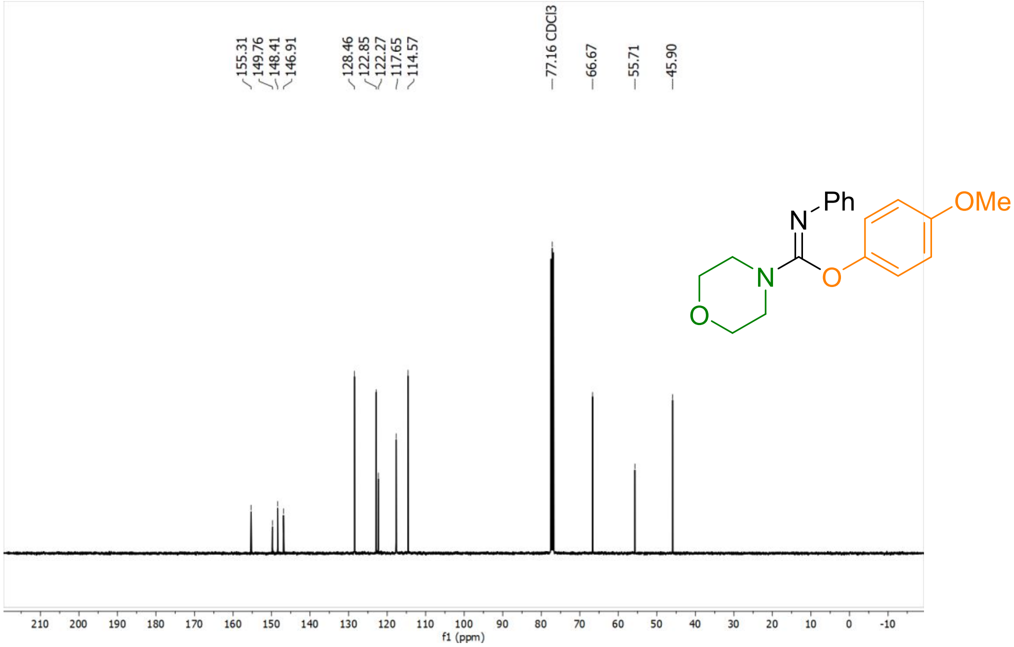

4-(trifluoromethyl)phenyl (Z)-N-phenylmorpholine-4-carbimidatecarbimidate (4c)

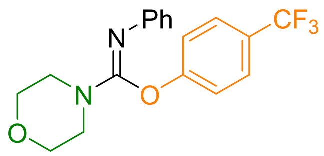

^{1}H NMR: 400 MHz in $CDCl_3$

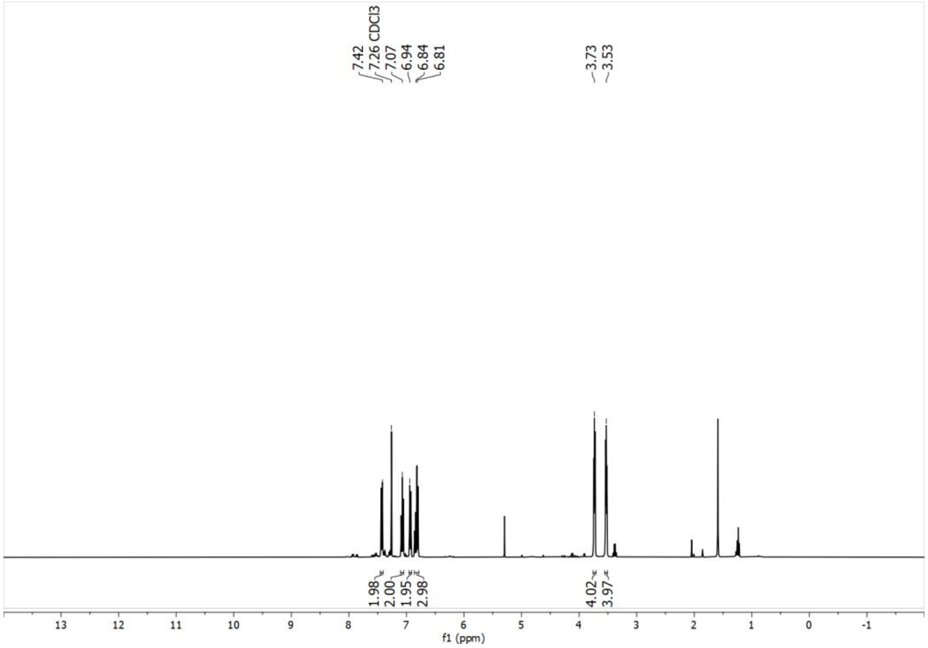

^{13}C NMR: 101 MHz in $CDCl_3$

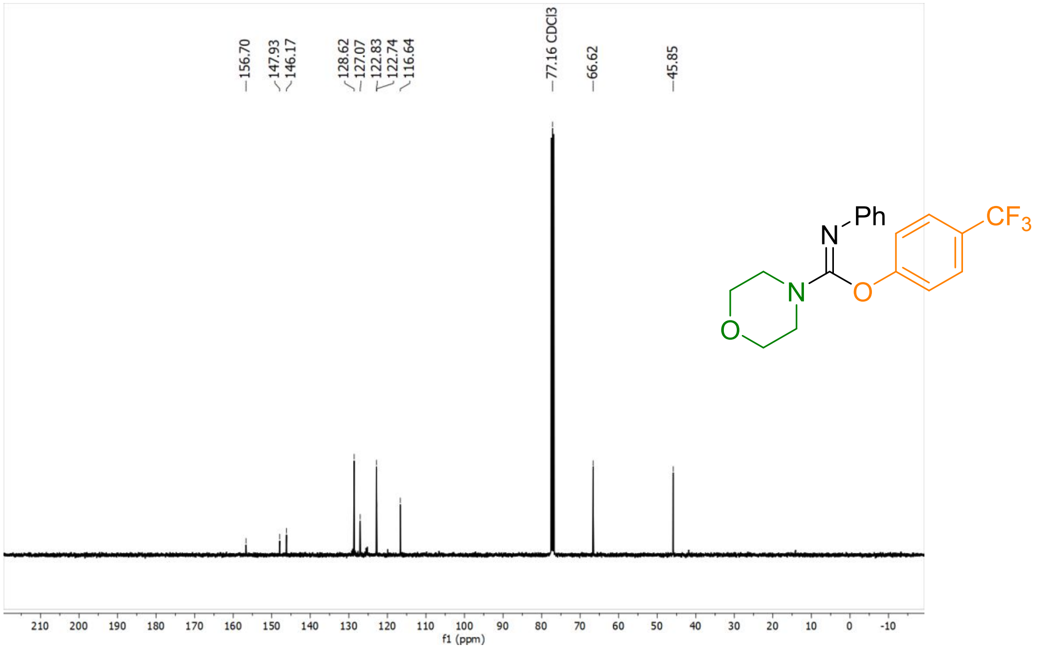

^{19}F NMR: 376 MHz in $CDCl_3$

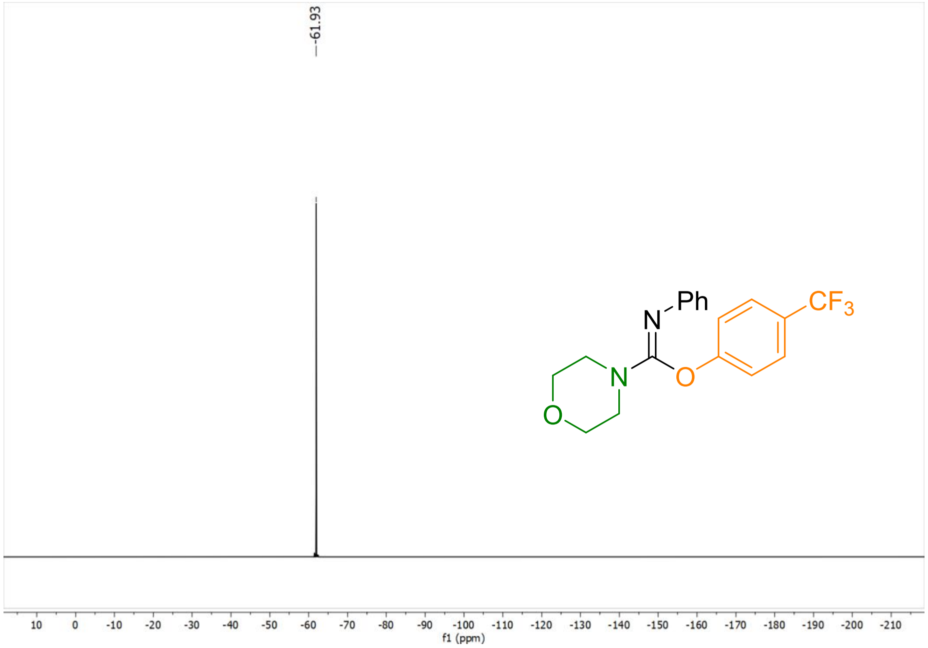

_o_-tolyl (Z)-N-phenylmorpholine-4-carbimidate (4d)

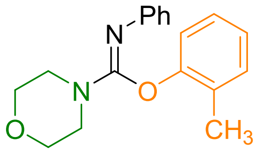

^{1}H NMR: 400 MHz in $CDCl_3$

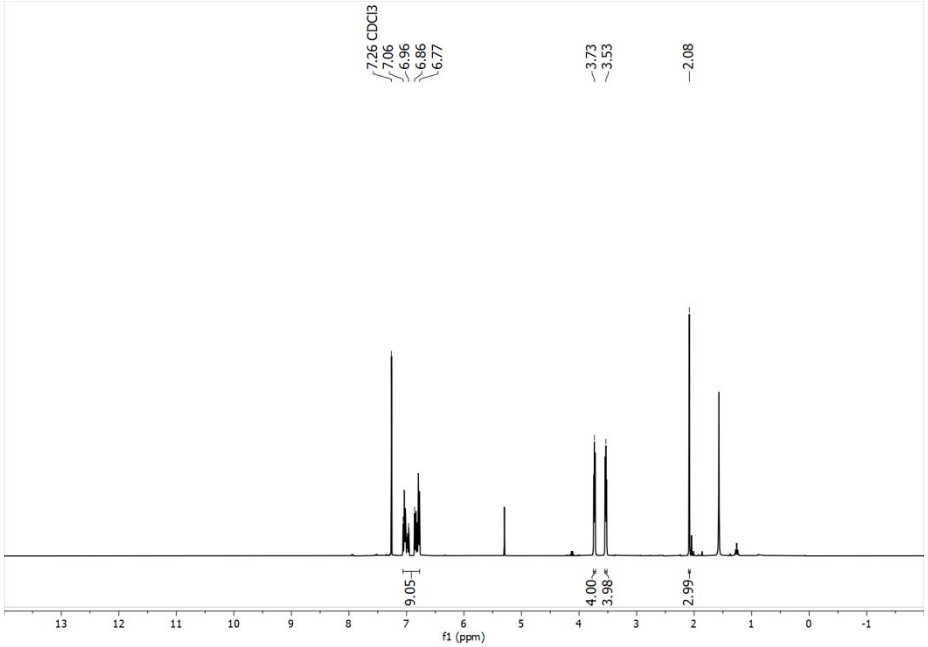

^{13}C NMR: 101 MHz in $CDCl_3$

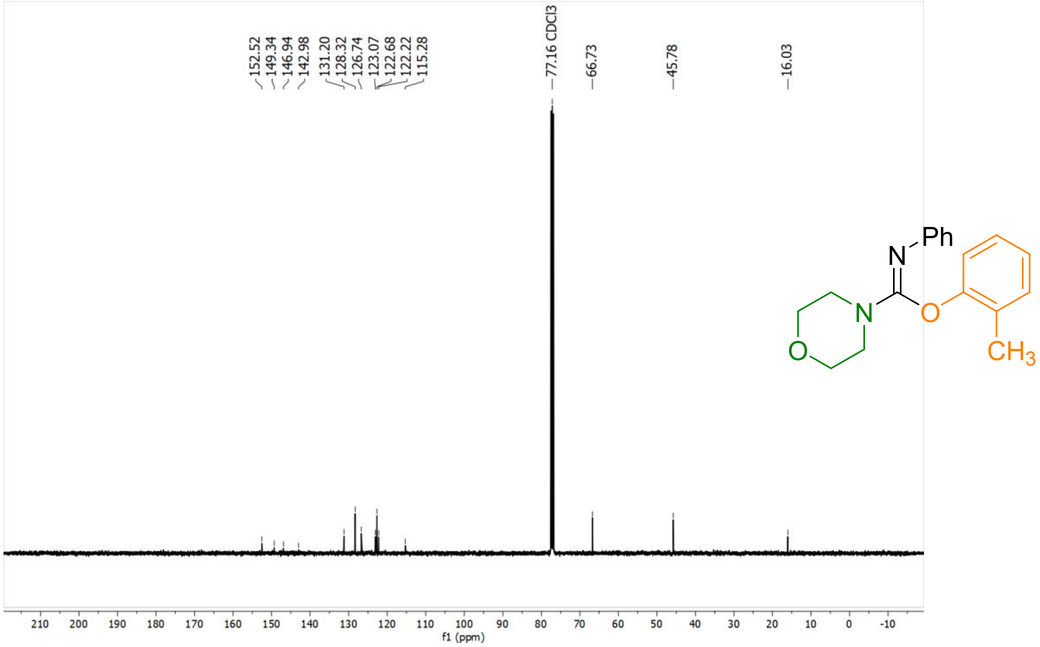

4-formyl-2-methoxyphenyl (Z)-N-phenylmorpholine-4-carbimidate (4e)

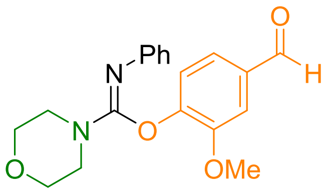

^{1}H NMR: 400 MHz in $CDCl_3$

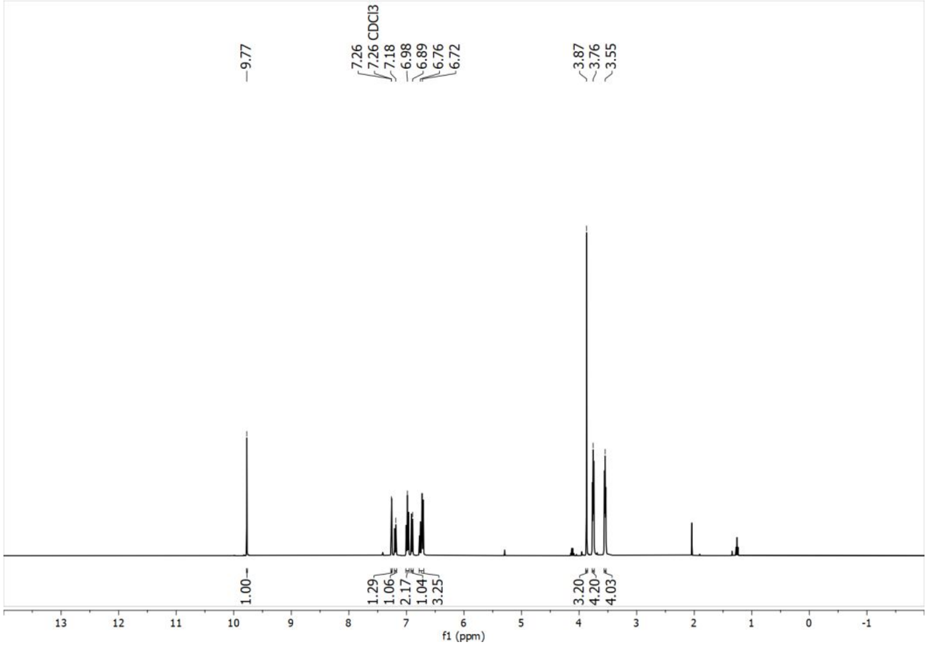

^{13}C NMR: 101 MHz in $CDCl_3$

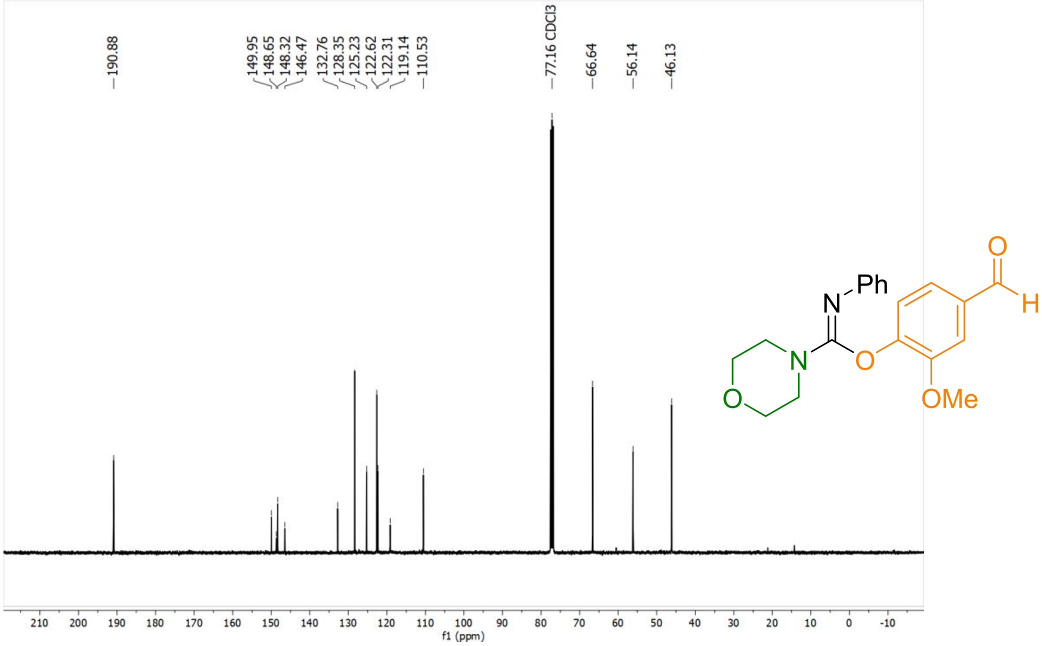

2-oxo-2*H*-chromen-7-yl (Z)-N-phenylmorpholine-4-carbimidate (4f)

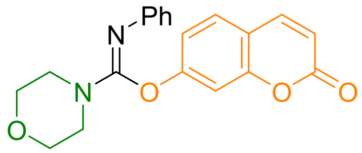

^{1}H NMR: 400 MHz in $CDCl_3$

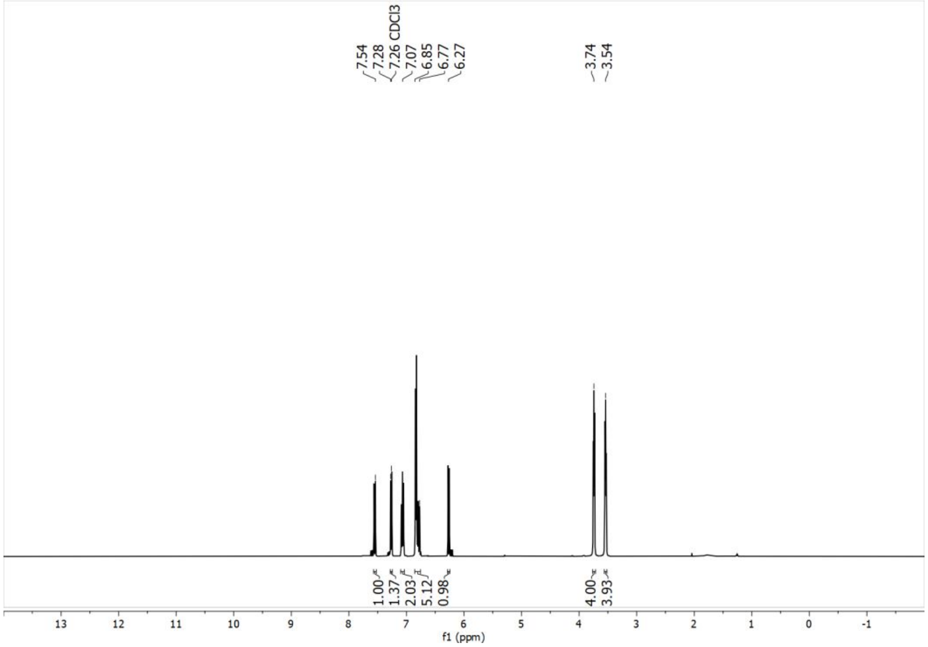

^{13}C NMR: 101 MHz in $CDCl_3$

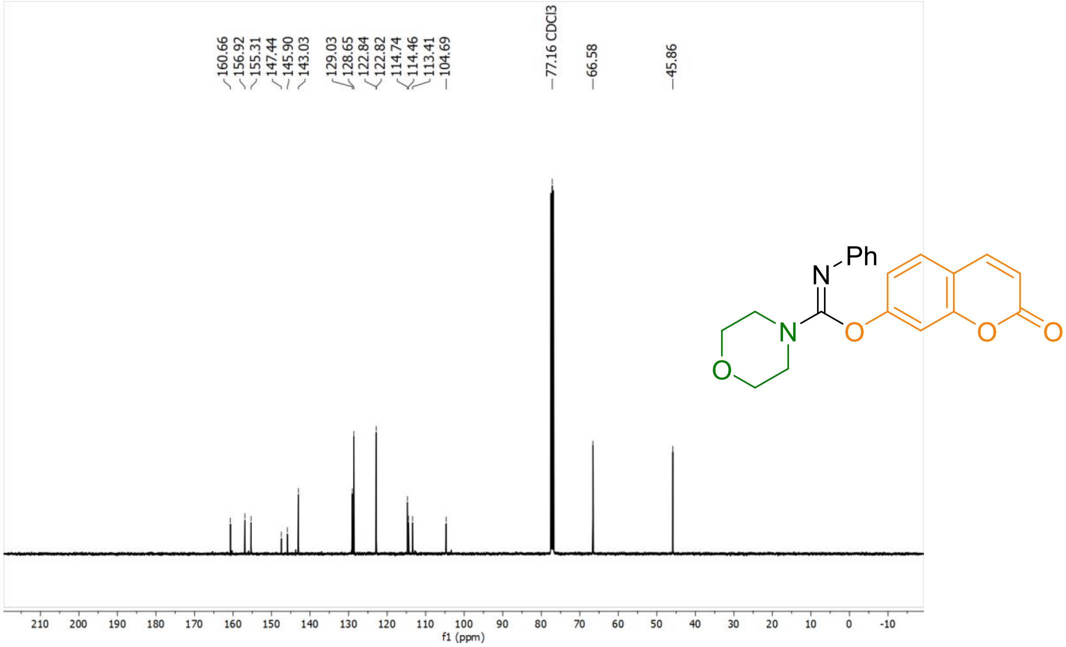

quinolin-4-yl (Z)-N-phenylmorpholine-4-carbimidate (4g)

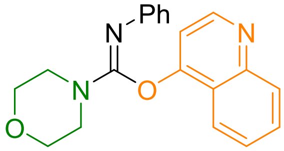

^{1}H NMR: 400 MHz in $CDCl_3$

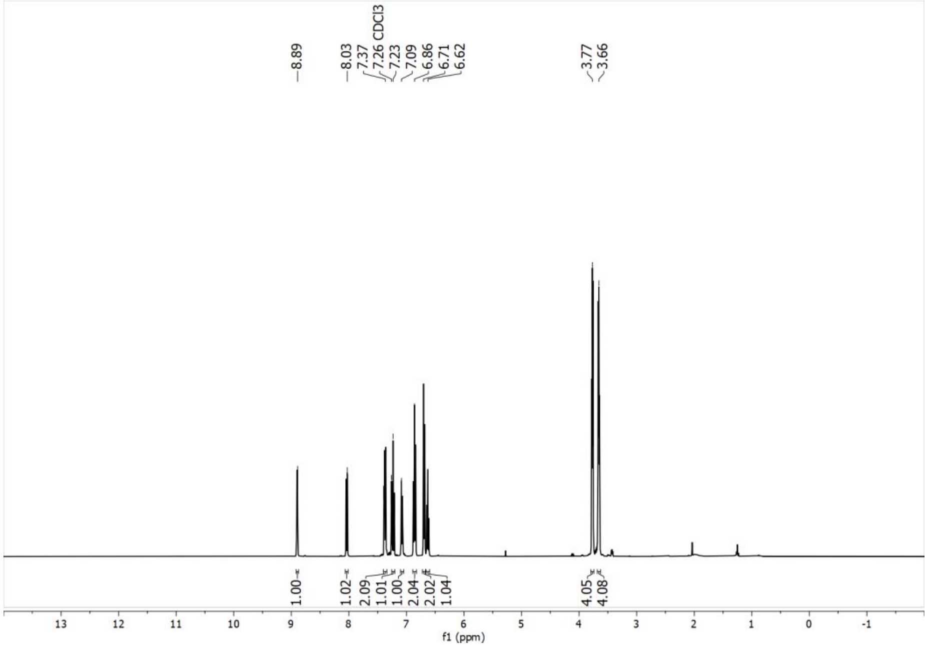

^{13}C NMR: 101 MHz in $CDCl_3$

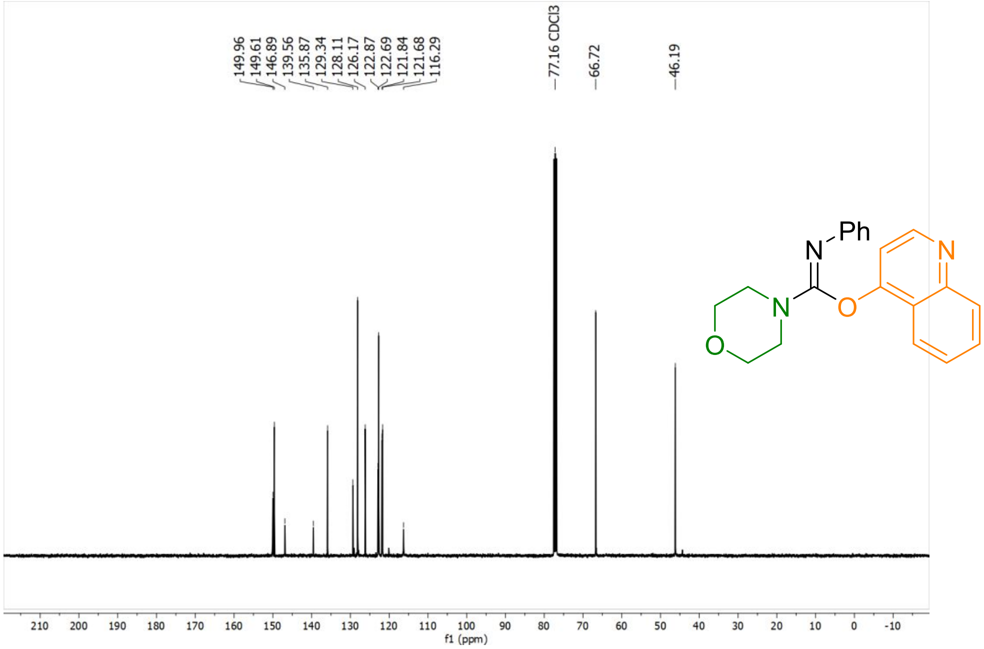

phenyl (Z)-N-phenylmorpholine-4-carbimidothioate (4h)

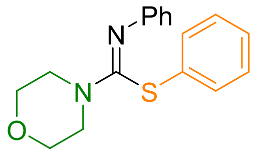

^{1}H NMR: 400 MHz in $CDCl_3$

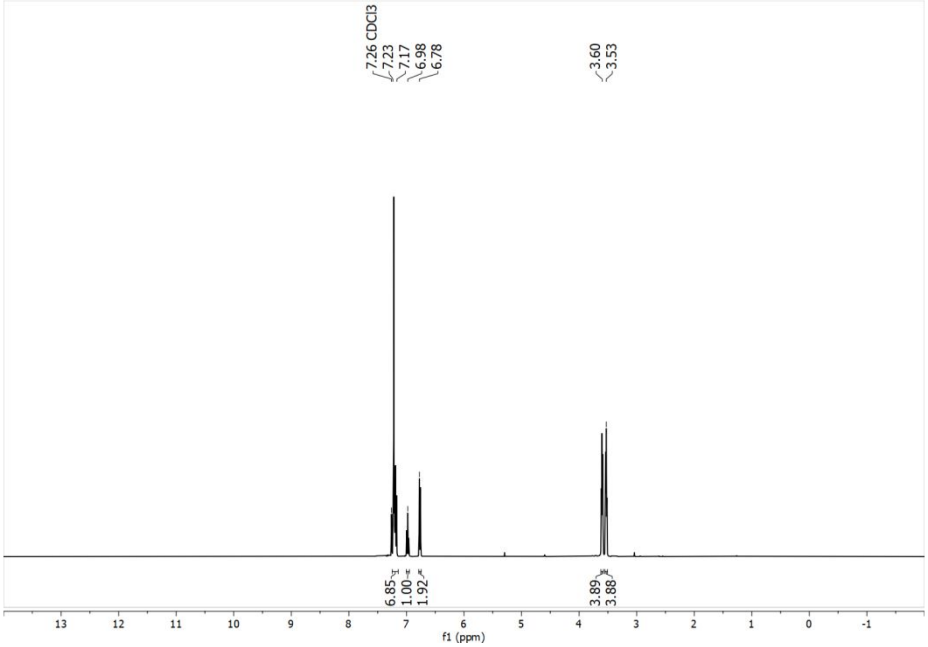

^{13}C NMR: 101 MHz in $CDCl_3$

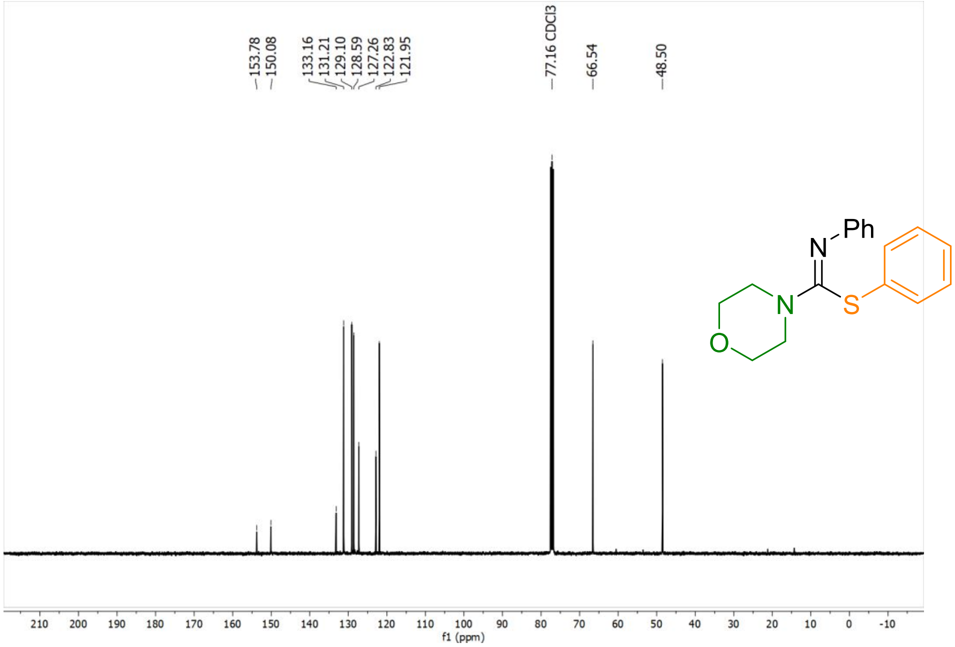

phenyl (Z)-N-(4-(trifluoromethyl)phenyl)morpholine-4-carbimidate (4i)

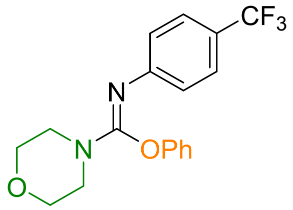

^{1}H NMR: 400 MHz in $CDCl_3$

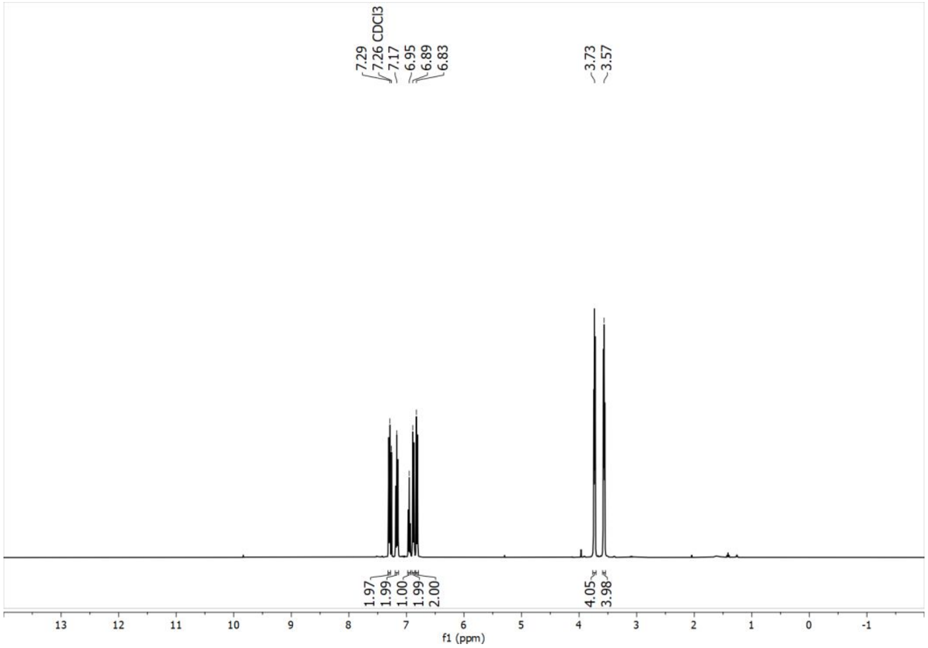

^{13}C NMR: 101 MHz in $CDCl_3$

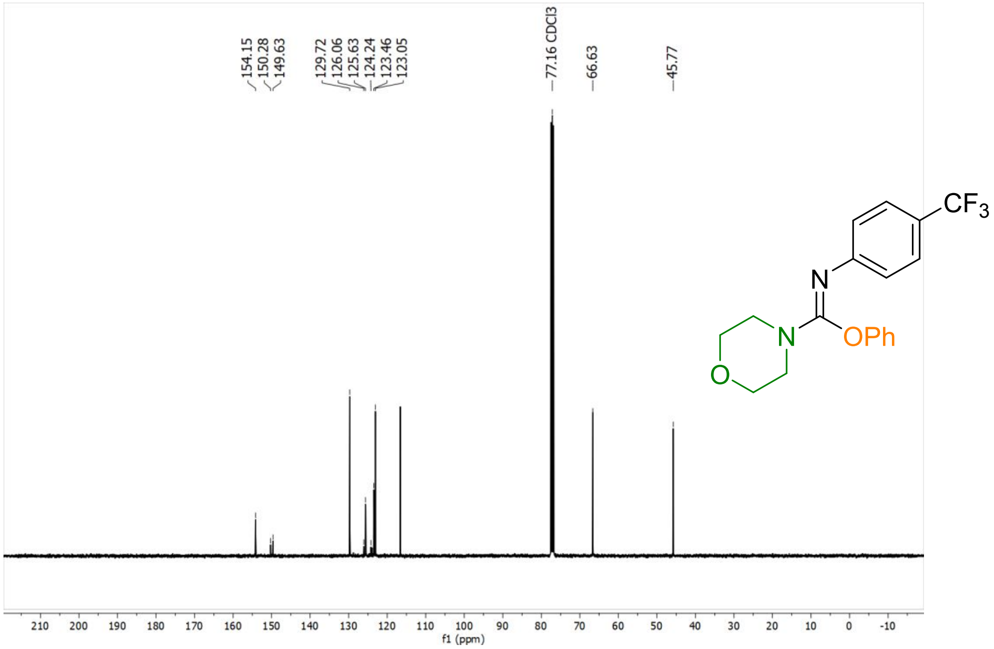

^{19}F NMR: 376 MHz in $CDCl_3$

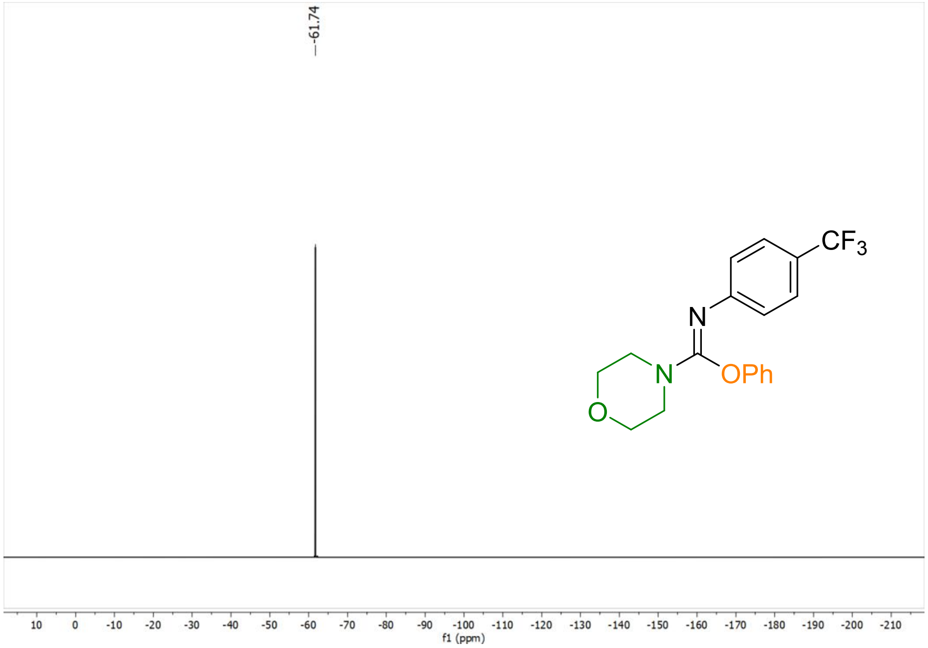

phenyl (Z)-N-(6-methoxypyridin-3-yl)morpholine-4-carbimidate (4j)

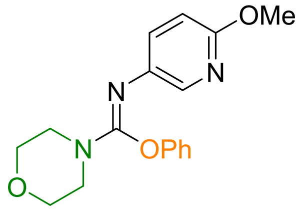

^{1}H NMR: 400 MHz in $CDCl_3$

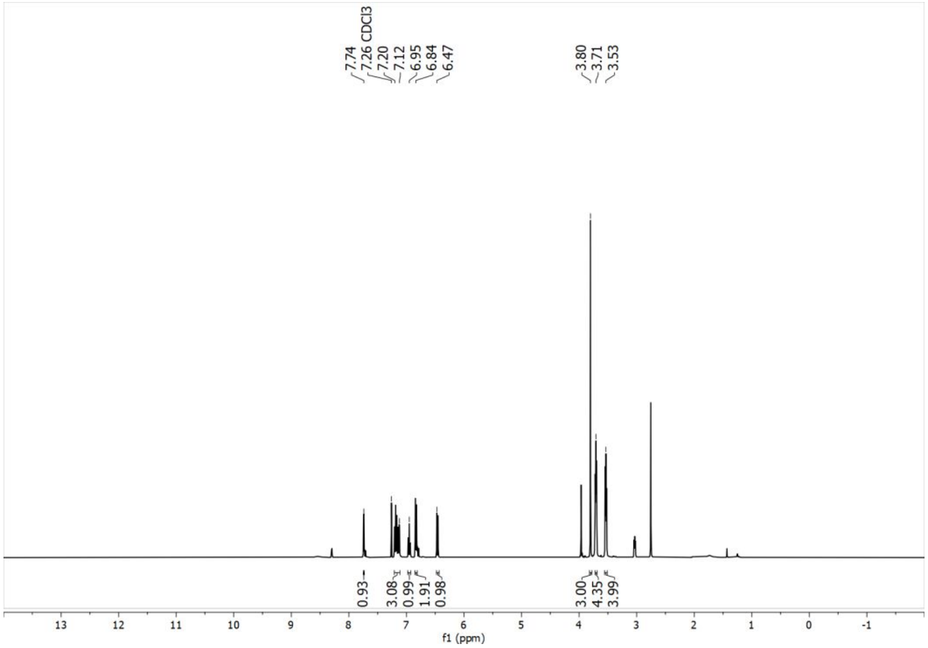

^{13}C NMR: 101 MHz in $CDCl_3$

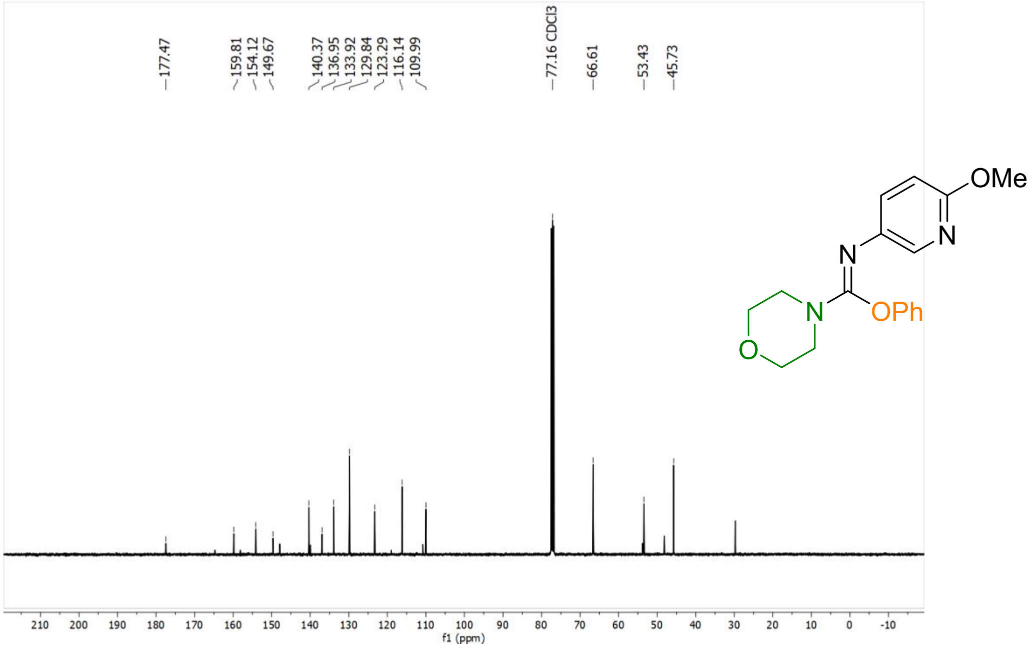

phenyl (Z)-N-((E)-styryl)morpholine-4-carbimidate (4k)

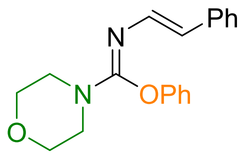

^{1}H NMR: 400 MHz in $CDCl_3$

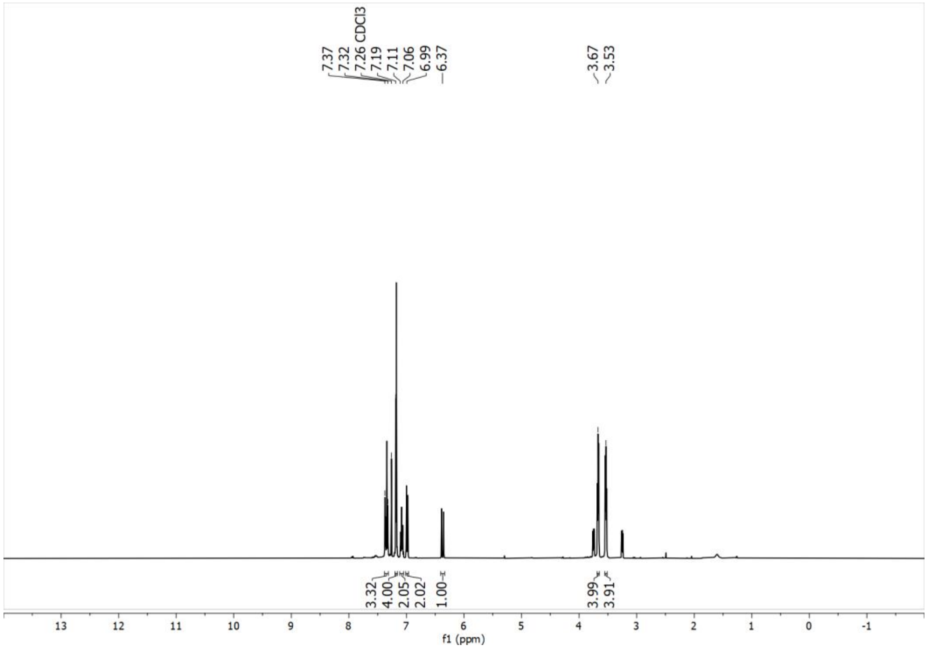

^{13}C NMR: 101 MHz in $CDCl_3$

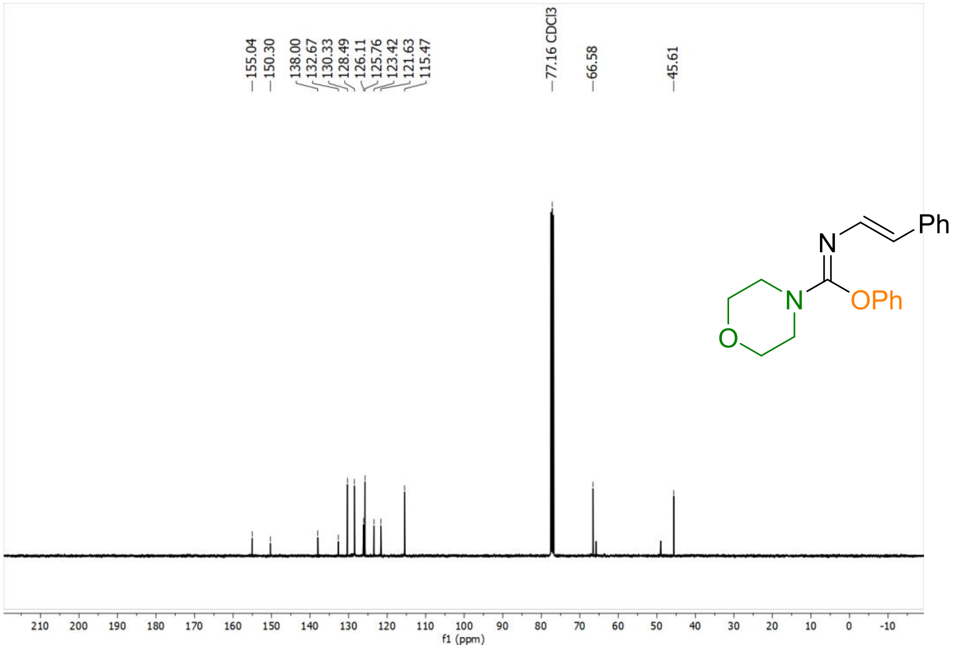

phenyl (Z)-N-phenyl-1,4-dioxa-8-azaspiro[4.5]decane-8-carbimidate (4l)

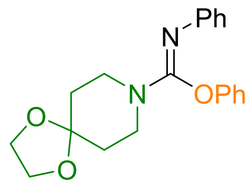

^{1}H NMR: 400 MHz in $CDCl_3$

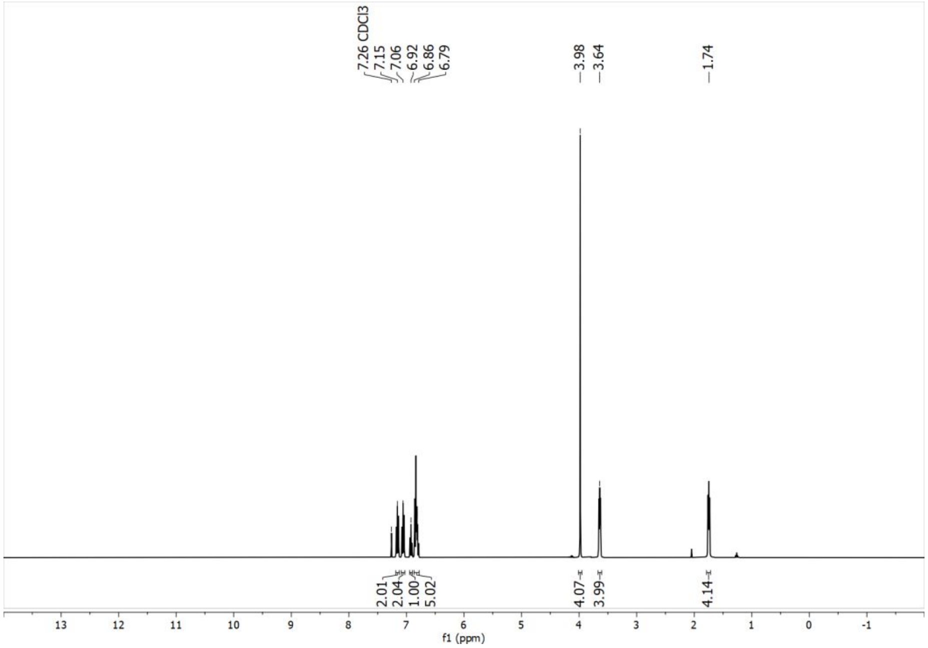

^{13}C NMR: 101 MHz in $CDCl_3$

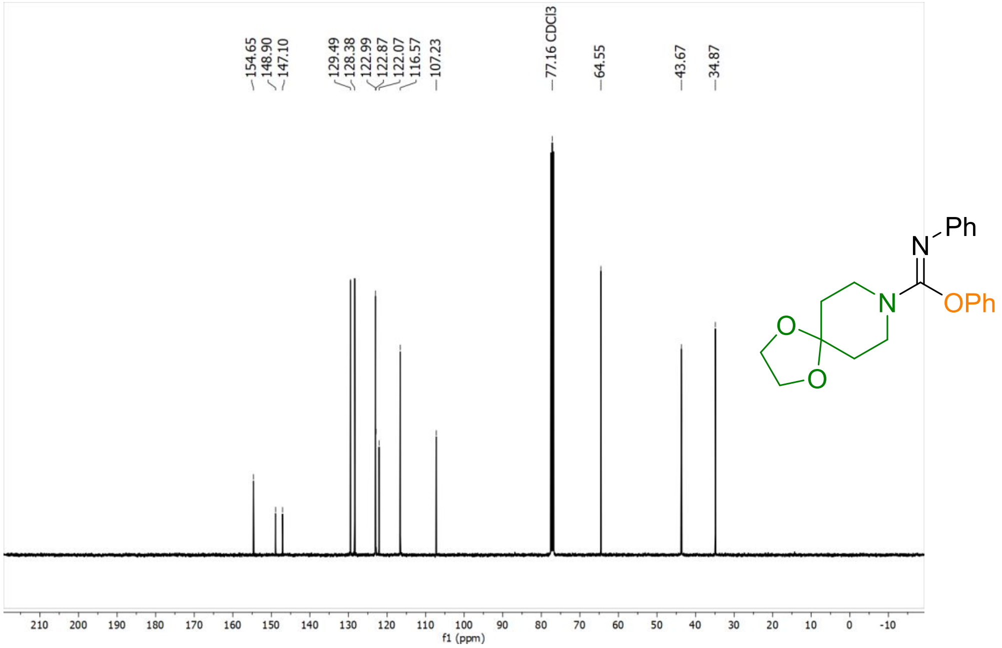

benzyl (Z)-4-(phenoxy(phenylimino)methyl)-1,4-diazepane-1-carboxylate (4m)

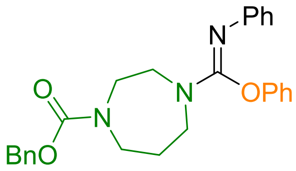

^{1}H NMR: 400 MHz in $CDCl_3$

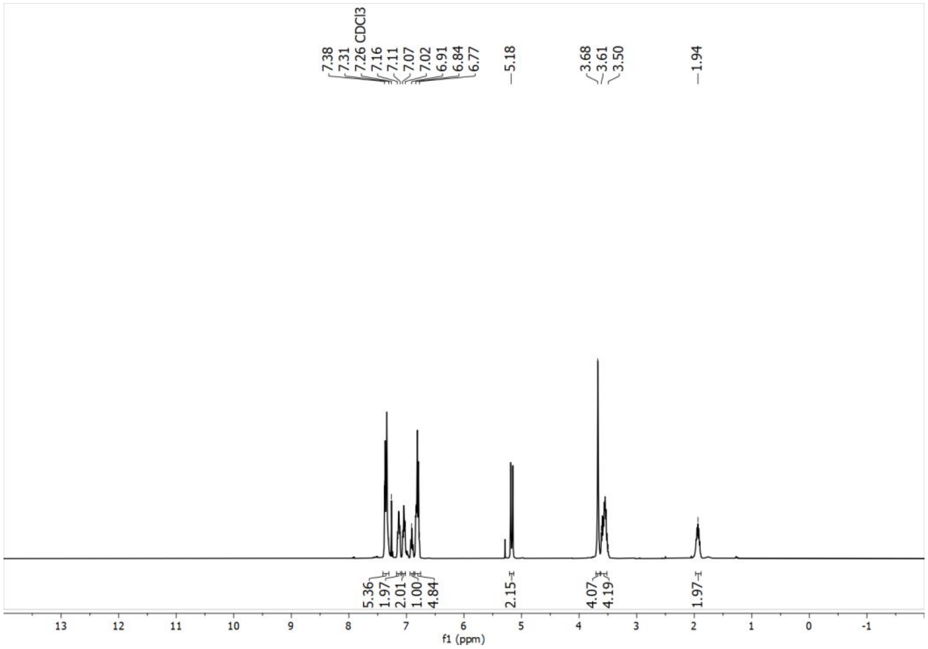

^{13}C NMR: 101 MHz in $CDCl_3$

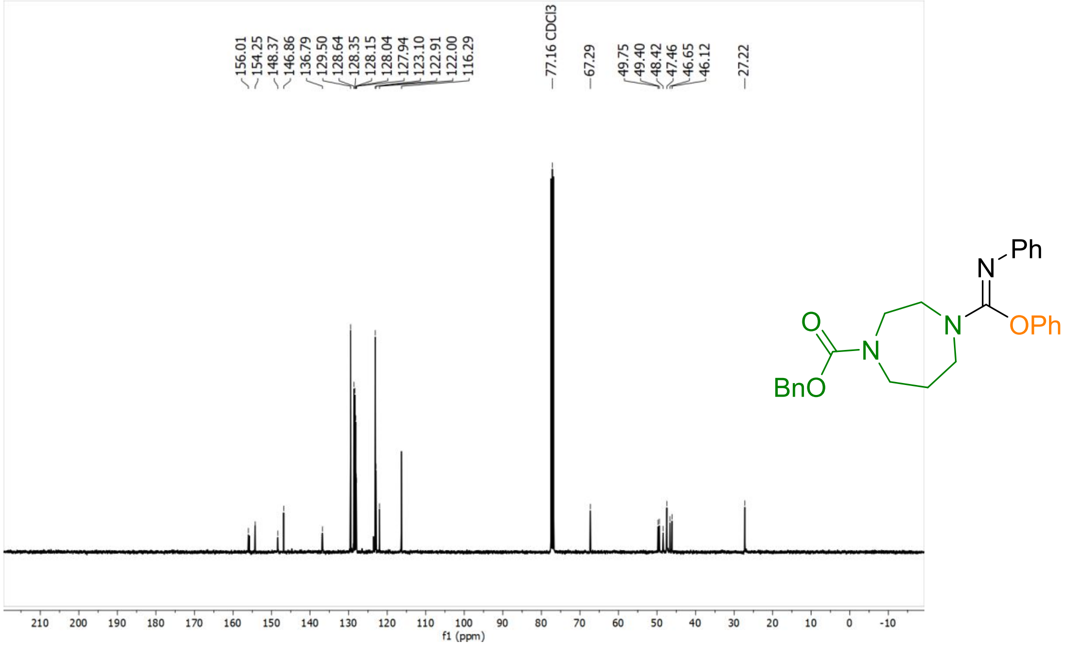

12. References

(1) Vogel, J. A.; Hammami, R.; Ko, A.; Datta, H.; Eiben, Y. N.; Labenne, K. J.; McCarver, E. C.; Yilmaz, E. Z.; Melvin, P. R. Synthesis of Highly Reactive Sulfone Iminium Fluorides and Their Use in Deoxyfluorination and Sulfur Fluoride Exchange Chemistry. *Org. Lett.* **2022**, *24* (32), 5962-5966. DOI: 10.1021/acs.orglett.2c02232.

(2) Dalby, K. N.; Jencks, W. P. Lifetimes of Imidinium Ions in Aqueous Solution. *J. Am. Chem. Soc.* **1997**, *119* (31), 7271-7280. DOI: 10.1021/ja970628i.

(3) Iwasaki, T.; Tsuge, K.; Naito, N.; Nozaki, K. Chemoselectivity change in catalytic hydrogenolysis enabling urea-reduction to formamide/amine over more reactive carbonyl compounds. *Nature Communications* **2023**, *14* (1), 3279. DOI: 10.1038/s41467-023-38997-2.

(4) Matsutani, T.; Aoyama, K.; Moriuchi, T. Oxovanadium(V)-Catalyzed Synthesis of Unsymmetrical Ureas by Activation of Carbon Dioxide under Ambient Pressure. *Organometallics* **2023**, *42* (12), 1310-1316. DOI: 10.1021/acs.organomet.2c00623.

(5) Grzyb, J. A.; Shen, M.; Yoshina-Ishii, C.; Chi, W.; Brown, R. S.; Batey, R. A. Carbamoylimidazolium and thiocarbamoylimidazolium salts: novel reagents for the synthesis of ureas, thioureas, carbamates, thiocarbamates and amides. *Tetrahedron* **2005**, *61* (30), 7153-7175. DOI: https://doi.org/10.1016/j.tet.2005.05.056.

(6) Peterson, S. L.; Stucka, S. M.; Dinsmore, C. J. Parallel Synthesis of Ureas and Carbamates from Amines and CO2 under Mild Conditions. *Org. Lett.* **2010**, *12* (6), 1340-1343. DOI: 10.1021/ol100259j.
